# Movement Timing and Invariance Arise from Several Geometries

**DOI:** 10.1371/journal.pcbi.1000426

**Published:** 2009-07-10

**Authors:** Daniel Bennequin, Ronit Fuchs, Alain Berthoz, Tamar Flash

**Affiliations:** 1 Équipe Géométrie et Dynamique, Institut de Mathématiques de Jussieu, UMR 7586, Paris, France; 2 Department of Computer Science and Applied Mathematics, Weizmann Institute of Science, Rehovot, Israel; 3 Laboratoire de Physiologie de la Perception et de l'Action, Collège de France, Paris, France; Indiana University, United States of America

## Abstract

Human movements show several prominent features; movement duration is nearly independent of movement size (the isochrony principle), instantaneous speed depends on movement curvature (captured by the 2/3 power law), and complex movements are composed of simpler elements (movement compositionality). No existing theory can successfully account for all of these features, and the nature of the underlying motion primitives is still unknown. Also unknown is how the brain selects movement duration. Here we present a new theory of movement timing based on geometrical invariance. We propose that movement duration and compositionality arise from cooperation among Euclidian, equi-affine and full affine geometries. Each geometry posses a canonical measure of distance along curves, an invariant arc-length parameter. We suggest that for continuous movements, the actual movement duration reflects a particular tensorial mixture of these canonical parameters. Near geometrical singularities, specific combinations are selected to compensate for time expansion or compression in individual parameters. The theory was mathematically formulated using Cartan's moving frame method. Its predictions were tested on three data sets: drawings of elliptical curves, locomotion and drawing trajectories of complex figural forms (cloverleaves, lemniscates and limaçons, with varying ratios between the sizes of the large versus the small loops). Our theory accounted well for the kinematic and temporal features of these movements, in most cases better than the constrained Minimum Jerk model, even when taking into account the number of estimated free parameters. During both drawing and locomotion equi-affine geometry was the most dominant geometry, with affine geometry second most important during drawing; Euclidian geometry was second most important during locomotion. We further discuss the implications of this theory: the origin of the dominance of equi-affine geometry, the possibility that the brain uses different mixtures of these geometries to encode movement duration and speed, and the ontogeny of such representations.

## Author Summary

No existing theory successfully accounts for several amazing properties of biological movements: dependence of movement speed on path curvature, isochrony (movement duration is nearly independent of its size) and the construction of more complex movements from simpler building blocks. Here we present a new theory of movement generation, based on movement invariance with respect to geometrical transformations. Several types of transformations are considered. Euclidian transformations preserve lengths and angles; affine transformations, which are less restricted, preserve parallelisms between lines, while equi-affine transformations preserve both parallelism and area. Each geometry is associated with a different measure of distance along curves. Movement timing is continuously prescribed by the brain by combining different “geometrical times” each assumed to be proportional to the measure of distance of the corresponding geometry. Movements are constructed by using a series of instantaneous (Cartan) coordinate frames. The predictions of the theory compared well with experimental observations of human drawing and walking. Equi-affine geometry was found to play a dominant role in both tasks and is complemented by affine geometry during drawing and by Euclidian geometry during locomotion. The proposed theory has far reaching implications with respect to brain representations of motion for both action production and perception.

## Introduction

### Affine geometry and motion

As a first approximation, perceived physical space is assumed to be Euclidian. Yet, psychophysical studies of visual perception, drawing movements and locomotion indicate important departures from Euclidian geometry [1]–[4]. In these cases, space and movements seem to be perceived in terms of affine geometrical properties [2], [5]–[8]. Affine geometry is the geometry which retains from Euclidian geometry only the existence of points, lines and planes with their geometrical properties of incidence (i.e., the existence of only one straight line between two points) and parallelism (i.e. the existence of a unique line parallel to a given line that passes through a given point, Thales' theorem, etc.). The study of affine geometry can also be based on displacement of points by vectors, (see section A in Text S1). There is no preferred absolute distance in affine geometry.

Most important in affine geometry is the set of affine transformations, which are transformations of space or of a plane transforming straight lines into straight lines and parallel lines into parallel lines. Concretely, affine transformations are obtained by composing together translations and linear mappings which include rotations, stretching and dilatations. A property of a geometrical shape is said to be affine invariant when it is preserved under all possible affine transformations. For instance, being a closed curve is an affine invariant property but enclosing an area equal to 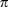 is not. Being an ellipse is an affine property, but being a circle is not, since any given circle and any elongated ellipse can be transformed one into the other using at least one affine transformation.

Since we are interested in motion timing, it is important to understand the concept of invariant duration with respect to a given set of transformations. For instance, a timing rule for a given set of curve segments is affine invariant if the duration spent moving along any arc of any one curve is equal to that spent moving along the image of this segment obtained by using any affine transformation. Thus, if timing were a totally affine invariant for all possible planar movements, all elliptical trajectories, for example, would have had the same total duration, generating a complete isochrony. We will show how such an invariance follows from a particular dependence of motor timing on the curvature of trajectories.

The influence of path curvature on movement velocity is well known [9]–[11]. Originally, Viviani and Terzuolo [1] claimed that movement velocity 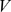 is roughly proportional to the radius of curvature 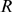 of a curved movement, i.e. 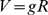, and that movement segmentation is determined by the presence of inflection points. Modifying this earlier suggestion and based on empirical observations, Lacquaniti et al. [12] formulated the two-thirds power law, stating that the instantaneous angular velocity 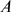 is proportional to the instantaneous curvature raised to the power 2/3*rd*. Equivalently, since 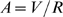, an alternative formulation of this law is: 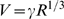, where 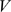 is the ordinary tangential velocity and 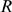 is the radius of curvature. The coefficient 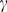 was termed the velocity gain factor and was shown to be piecewise constant.

Examining the dependence of 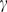 on the perimeter 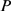 during periodic drawings of different figural forms (circles, ellipses, figure eights, double ellipses etc.), Viviani and McCollum [13] obtained a relationship consisting of multiplying two power laws, such that 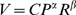 where 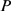 is the Euclidean perimeter of the figural form and 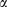 and 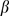 are empirically determined exponents. These observations, where the value of the velocity gain factor 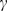 depends on the perimeter of the curve being drawn, were thought to account for the isochrony principle. This principle captures the empirical observation that the durations of movements involved in the generation of motion paths with similar geometrical forms but with different lengths are nearly equal [4], [9], [13]–[15].

The 2/3 power law was extended to human locomotion [16],[17] with a somewhat different set of exponents and was also linked to the perception of visual motion [18],[19]. Pollick and Sapiro [5] and Handzel and Flash [6],[20] have further suggested that the 2/3 power law is equivalent to movements being performed at a “constant equi-affine speed”, defined as the time derivative of 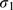, the equi-affine arc-length. Using the Euclidian radius of curvature and arc-length, 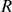 and 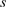, respectively, the equi-affine arc-length 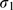 is defined as:
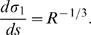
(1)According to this definition, the equi-affine length 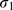 corresponds to the integral of the infinitesimal regular Euclidian arc-length 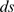 weighted by the Euclidian curvature raised to the power of 1/3. Thus, among equally long segments, those with greater curvature have longer equi-affine length. Based on this approach, Flash and Handzel [8] further developed a framework using group theory to describe and analyze human movements. However, a significant empirical observation which was not accounted for by the equi-affine description, nor by any other model, is the observed tendency towards global isochrony of human movement, mentioned above. Moreover, neither the equi-affine description, nor any other model has made any explicit suggestion as to how the values of the velocity gain factor are selected for in any movement segment.

As part of our new approach, we treat movement generation as being based on full affine geometry, without making specific choices of units of length or area. This allows us to compare the influences of affine, equi-affine, and Euclidian geometries on the temporal properties of the movements. We suggest that in each of these geometries time is proportional to a specific “measure of distance along the curve” in that particular geometry. In addition, we deduce the variation of the velocity gain factor from the need for full-affine invariance. This results in a local form of isochrony. For instance, for movements along ellipses, a full affine invariance predicts the same 2/3 principle as equi-affine invariance. But, in addition, it predicts that through appropriate adaptation of the “velocity gain factor” movement duration will be the same for all ellipses in the plane.

However, total isochrony may sometimes lead to paradoxical behavior, and we therefore hypothesize that the brain takes advantage of the existence of several possible geometries rather than using a single geometry. Hence, we suggest that movement timing is continuously prescribed and realized according to an equilibrium between affine and Euclidian geometries with Equi-affine transformations, which are the area-preserving affine transformations, playing an essential, if not dominant, role [5],[6],[8]. Combining geometries is a totally new approach; it has not been previously considered in mathematics nor in biology, let alone in motor control or vision research. Here, using this new approach to the timing of motion, we derive new guidelines for motion segmentation and for the identification of motion primitives, while treating both hand trajectories and locomotion within the same framework.

The idea that geometric invariance is of great importance in prescribing the principles underlying perception and action is quite old [21]–[23]. Since that time many psychophysical studies have discussed the importance of invariance theory for perception [24]–[27]. Summarizing informally (see [26]), an invariant entity came to mean “anything which is left unaltered by selected transformations” [28]. However, the concept of invariance has benefited from mathematical formulation as initiated by Galois [29] (see [23], [30]–[32]), and it is this concept of invariance that serves as a conceptual basis for our theory. Galois has stated that in solving any given equation, it is more important to understand the structure of the ambiguity among all the possible solutions of this equation, rather than trying to directly derive them. Moreover, this roundabout approach frequently offers better means for computing such solutions. Different levels of analysis of the given equation are characterized by the sets of transformations of the solutions which are equivalent at those particular levels of analysis.

It is natural to propose that, in the same way, the brain uses several levels of representation and processing in planning any particular motion. At each level the computation is organized by respecting certain symmetries. Approaching the level of motor execution fewer and fewer possibilities are allowed, thus reducing the initial larger group of symmetry of all possible movements into smaller groups. This hierarchy of decisions in motion planning and execution is reflected in the representation of space through the performed movement.

In the following mathematical section, which discusses full affine, equi-affine and Euclidian geometries, we use Cartan's moving frame method [33]–[35], whose main theme is the relation between a specific curve and the action of a group of transformations of frames defined along that curve. For instance, given a specific group of transformations we look for a parameterization of the curve which is invariant under such transformations. When curve segments are similar under transformations belonging to that group, the parameterization of these segments will also be similar. In particular, we show how Cartan's moving frame method is well suited for our problem of trajectory planning and segmentation.

### Mathematical preliminaries

From the work of Galois [29], Cayley, Jordan, Lie, [36],[37], Poincare [22] and Klein (cf. [38],[39]), we see that a particular geometry is captured by a particular group of transformations 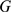 of the points of a space or of a plane 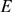, such that every point or every direction in 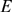 can be transformed by an element of 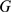 to every other point or direction. Euclidian geometry corresponds to 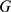 being the group of rigid displacements consisting of translations and rotations but we can also choose 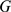 to be the group of all possible affine transformations, generated by *translations*, *rotations*, *reflections*, but also by *dilatations*, *stretching* and *shearing*. Or we can choose the *special* subgroup of the full-affine group, namely the area-preserving equi-affine group, which includes all the above transformations except for dilatations. The last two groups correspond to the full affine and to the equi-affine geometries, respectively. These three geometries - Euclidean, full affine and equi-affine - are the most important geometries for the two-dimensional (2D) plane 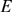, in our present investigation.

*A priori* the largest possible group of invariance to be considered is that containing all continuous smooth transformations of geometrical objects in the plane. This group, however, does not provide sufficient constraints, since all possible trajectories are equivalent under such transformations and are expected to have the same duration. Another possibility is using the group of projective transformations (consisting of compositions of several pairs of perspective projections and describing changes in the perceived positions of observed objects when the point of view of the observer changes). However, some projective transformations paradoxically send points at a finite distance to infinity. The affine group 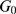 is formed by projective transformations for which this does not happen. Thus, it describes the largest possible reasonable invariance. At the other extreme is the group of isometries, i.e. the Euclidian group 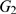 of length-preserving rigid transformations. The sub-group 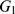 of equi-affine transformations lies between 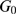 and 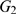.

Curves can be analyzed differently in different geometries (see Monge [39], Lie and Cartan [33]). Cartan's method generalizes the *moving frame method* originally developed by Darboux [39]. Note that, for the 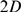 case, any frame is formed by a point and by two attached basis vectors (see section A in Text S1).

The essence of Cartan's moving frame method is that it creates a correspondence between the different orders of description of trajectories and the possible coordinate frames on the plane. This method specifies which geometrical transformations of frames allow identification of the trajectories that are indistinguishable at a given order (see section A in Text S1).

At each point in time, locations within the plane are represented by coordinates in a moving frame. The motion along any curve is described by the equations representing the new infinitesimal coordinate frame (the new location and the new basis vectors) within the instantaneous current frame. When the moving frame is the canonical moving frame, the only remaining varying coefficient is the *instantaneous curvature*. This is an invariant quantity of the geometrical curve in the geometry defined by the group of transformation 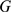. Thus, when using the moving frame description all along the curve, the representation of the infinitesimal next frame uses only invariant quantities and, in this sense, is the simplest possible one.

The choice of parametrization of the curve necessary for deriving the canonical form of the moving frame gives a unique parameter, which is also the only parameter invariant under any transformation belonging to the group 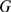 of the chosen geometry. In full affine geometry, this unique parameter is called the *full affine arc-length* and it is denoted by 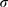 (see section A.2 in Text S1). With the same kind of analysis in Euclidian geometry, we obtain a canonical parametrization by using the ordinary Euclidian distance (arc-length) 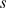 instead of 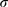, while in equi-affine geometry we obtain canonical parametrization by using the equi-affine arc length 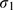.

To connect this description with known kinematic notions, choosing time to be proportional to the Euclidian arc length 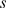 gives rise to an ordinary uniform Euclidian motion, i.e., to a motion with a constant tangential Euclidian velocity 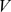. Setting time to be proportional to 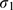 gives rise to a motion with a constant equi-affine speed. This is a motion whose tangential velocity 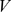 obeys 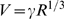, where 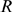 is the Euclidian radius of curvature and 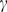 which is a constant, is the so called *velocity gain factor* defined by Lacquaniti et al. (1983).

Given a point 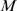 on a curve, there exists a unique equi-affine frame, centered at 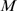 with coordinates 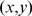, whereby the curve near the point 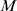 takes the following simple form, called the reduced equation of the curve:
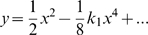
(2)Cf. [33]. This frame is the *equi-affine canonical frame*. The coefficient 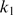, which depends on the point 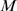 appearing in this equations, is the so called *equi-affine curvature*.

The *equi-affine moving frame equations* are the only infinitesimal equations for which the motion is expressed as follows:
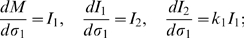
(3)where 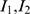 denote the basis unit vectors of the equi-affine canonical moving frame (see section A.2 in Text S1).

The mathematical expression for the equi-affine curvature when expressed as a function of Euclidian radius of curvature 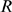 is:
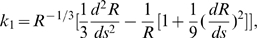
(4)where 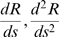 are, respectively, the first and second order derivatives of 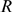 with respect to the Euclidian arc-length 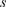. Equi-affine curvature is the quantity defined on a planar curve which remains invariant (unchanged) under equi-affine transformations. The curves of constant equi-affine curvature 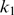 are all plane conics. Those with 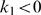 are ellipses, those with 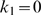 are parabolas, and those with 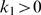 are hyperbolas.

Focusing now on the canonical full affine moving frame, the derivative of the full affine arc-length 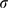 with respect to the Euclidian arc-length 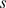 is expressed by:
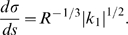
(5)


When time is set proportional to the full affine arc-length 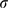, the velocity gain factor varies according to the equation: 
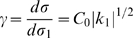
.

The canonical *full-affine moving frame* is simply obtained by scaling the vectors in (3): it is formed by 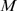 and by 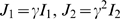 (see section A.2 in Text S1).

Another kind of curvature appears for the full affine frame. This full-affine curvature remains unchanged under all full affine transformations. It determines the relative variation of the velocity gain factor 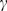 with the full affine arc-length 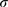:
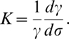
(6)


Two kinds of special points generically arise along a path immersed within the affine plane: ordinary inflection points (at which the Euclidean curvature equals zero) and ordinary parabolic points (at which the equi-affine curvature equals zero). Near ordinary inflection points, when the distance 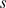 measured from the inflection point, tends to zero, 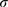 diverges like 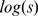 and 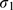 shrinks like 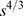 (see section A.3 in Text S1). Thus, as neither 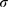 nor 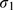 are defined at ordinary inflection points, neither affine nor equi-affine parameterizations can be used at or near such points. Similarly, near a parabolic point, 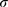 degenerates like 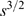, and only 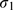 or 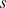 can be used.

However, as shown in section A in Text S1, by attributing a weight of 1/4 to the full affine arc-length and a weight of 3/4 to the equi-affine arc-length we get a parameter 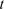 such that 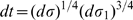, which offers a unique, convenient strategy for moving through an inflection point while keeping equi-affine invariance of time.

Summarizing the main results: at each point along a parameterized curve, locations within the plane are represented by coordinates in a moving frame. The motion along the curve is then expressed by equations representing the new location and the new basis vectors within the present frame. Given a group 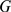 of plane transformations, there is a canonical moving frame along any curve. For this canonical moving frame, with its intrinsic arc-length parameter, the movement equations have the simplest possible form. The only changing coefficient is the *instantaneous curvature*, which is the only geometrical invariant of the curve in the geometry defined by 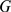. This results in the unique parametrization of the curves which is invariant under 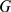. This parametrization is the only one for which the motion of the frames is of minimal complexity, in the sense that changing variables from the current to the next frame is represented by an invariant quantity.

## Results

### From geometry to time

We propose here that the brain selects movement timing and duration according to a principle of geometrical invariance. A few principles are necessary to derive timing and kinematics of motion from geometry. As mentioned above, all the geometries considered here define canonical coordinates along curves: 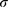, 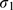 and 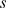 are the invariant arc-length parameters of the affine, equi-affine and Euclidian geometries, respectively. Using these parameters and assuming some specific relation between time and the corresponding parameter, the principle of the invariance of time becomes concrete.

By definition, we will call ***monotonic*** any movement or any part of a movement during which duration is proportional to one of these invariant parameters. That is, in the case of planar movements, the affine invariance selects time 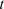 such that Δ*t = C*_0_Δσ. For movements with equi-affine invariance 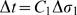, and for movements with Euclidian invariance 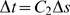. The constants 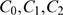 fix the scales of the corresponding durations but may also depend on the context, the subject and his/her intention to move quickly or slowly, etc.

Let us remark that monotony necessarily neglects the fact that the motions start and end at rest with zero velocities, accelerations, and higher order derivatives of position (i.e., that we also make discrete movements). Because of the presence of such boundary conditions, the model must be generalized by considering the canonical parameters 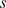, 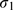 or 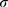 in the corresponding geometries (Euclidian, equi-affine or full affine, respectively) to be polynomials of some order of 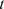. The first or second derivatives of these polynomials are zero at the movement end-points. This gives perfectly invariant timing for discrete trajectories which start and end at rest. Thus, any rule such that 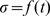 is affine invariant in the sense that if one applies an affine transformation to a given curve, then the time 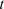 for the transformed curve follows the same rule. Similarly, a function 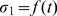 gives timing which is equi-affine invariant, and a function 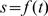 timing which is Euclidian invariant. This defines a clear general notion of “geometrical time” for all the three geometries, in which monotony is only the simplest possible case. However, in our initial presentation, all our tests of the validity of the new theory will be limited to periodic movements.

In many cases, only one geometrical parameter is insufficient for deriving movement kinematics and it is both necessary and useful to use a combination of several geometrical parameters.

Even for periodic motions the presence of singularities implies that monotony cannot be obeyed. For instance, the full affine parameter expands at an ordinary inflection point. Thus, if 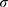 is the chosen time parametrization, it would take an infinite amount of time to reach an inflection point. In contrast, at an ordinary inflection point 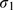 shrinks, requiring an infinite speed of movement when passing though such a point. The latter phenomenon also holds for parabolic points for the parameter 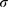, which shrinks near such points. *A priori* it would have been natural to expect that Euclidian geometry dominates near inflection points, because the curvature is zero at these points. However, our preliminary observations have indicated that this is not the case. To the contrary, it seems that at inflection points, Euclidian velocity is eliminated and a stereotypical mixture of affine and equi-affine velocities are used. This was the first case where we saw the advantage of having a mixture of several geometries. This conforms with mathematical analysis: as recalled above in the section sec∶math_pre, we showed (in section A in Text S1) that a unique combination of affine and equi-affine parameters offers a convenient strategy to passing through inflection points.

Combining several geometrical timing parameters offers greater flexibility and adaptivity to the motion planning strategy. Consequently, our general hypothesis is that ***movement duration results from the combined use of several geometries***.

That is, during some portion of any given movement the velocity will be more affine, while during another portion it will be more equi-affine or more Euclidian. This is formulated by expressing 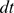 as a multiplication of some power functions of the canonical geometrical differential parameters 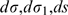. For simplicity we assume that there are intervals of time during which these combinations are stationary. Between these special intervals the time parameter is chosen by smooth interpolation.

The consequence of this hypothesis is the existence of a succession of segments belonging more or less to different fixed combinations of geometries. It is natural to expect that the existence of singular points such as inflection and parabolic points implies the presence of extended segments in their vicinity, during which a mixture of geometries are employed. Note that the global shape of a figural form often forces the presence of such singularities, thus we predict that the global shape of a trajectory will influence its local kinematics. When moving from one movement segment to the next, the transition between such segments should be smooth. Hence, all of the above additional guidelines can be summarized as giving rise to ***a tendency for expanding singularities, motion segmentation and smoothness***.

### Quantitative timing laws

To quantify the combination of geometries in selecting movement timing and total duration, we need to understand the different and modifiable weights attributed by the motor system to the various possible purely geometrical rules. For this purpose we propose an equation having an exponential form.

Let 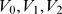 denote the expected Euclidian velocity under constant affine, equi-affine and Euclidian velocities, respectively, where time is proportional to the respective geometric parameters 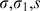. If 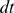 is proportional to 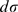, the Euclidian velocity is proportional to 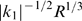 and we mark it by 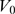. If 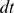 is proportional to 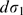, the Euclidian velocity is proportional to 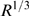 and we mark it by 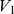 and if 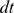 is proportional to 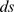, the Euclidian velocity is a constant, 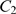, and we mark it by 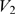. Hence the Euclidian velocities corresponding to these three different choices of time are:
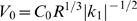
(7a)

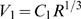
(7b)

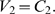
(7c)


We examined the recorded tangential Euclidian velocity 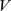 by assuming that the actually realized Euclidian velocity is prescribed according to the following product equation:
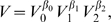
(8)where 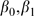 and 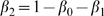 are weight functions defined along the trajectory with values lying within the range [0,1].

The above equation (10) can be rephrased using the following tensorial equation for movement duration:
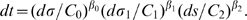
(9)A multiplicative form of the mixtures of velocities is more natural than an additive form because the parameters 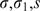 belong to different dimensions. In particular the treatment of the constants 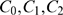 is technically easier when using a multiplicative form. Another reason became apparent during our mathematical analysis: as mentioned in the mathematical preliminaries section, it is possible to bypass inflection points by combining the logarithmic functions of the affine and equi-affine velocities using the weights of 1/4 and 3/4, respectively (see section A.3 in Text S1). Finally, the subjectively perceived velocity is related to the physical one by a nonlinear law, well approximated by a power law [40],[41], so the logarithmic function is able to associate the different velocities with their subjective perception.

Observe that, although affine transformations introduce additional complexities to the computations, the actual hypothesis to be tested is contained in equations (0), (10) above, which are easy to understand. Note that all 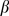 functions used here were determined based on the experimental data, except at inflection points where we have chosen them to be 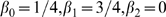 and at parabolic points where we selected 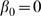. (See sections Experimental tests and Methods for the precise process.) Hence, at the present stage the theory is descriptive rather than predictive.

We have also tested less stringent consequences of using mixtures of invariance. In particular, we tested the possibility that during specific segments one geometry becomes more dominant than the others. A vivid manifestation of the existence of a pure geometric velocity can be obtained by comparing the times 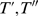 spent on two arcs 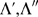 such that there is a planar affine transformation 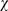 with 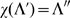:

Suppose that the time spent on a segment of a curve is affine invariant, even if affine velocity is not constant we have
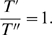
(10)


Suppose now that the law for duration is purely equi-affine. If 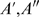 denote the total areas under respective arcs of curves and the corresponding secants (i.e., the total areas between the respective arcs of the curves (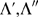) and the corresponding straight segments joining the extremities of those arcs in the plane), then
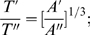
(11)Hence duration varies according to area.

When Euclidian geometry is the dominant kinematic law, if we denote the lengths of the arcs traveled by 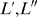, then
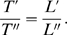
(12)


Now suppose that a curve 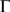 is a union of curved segments (not necessarily connected), 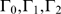, each being dominated by one of the three geometries, where each geometry is marked by one of three indexes (i.e. 0 for affine, 1 for equi-affine and 2 for Euclidian), and suppose that movement velocity varies continuously along 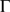. It can easily be shown from the continuity of the speeds at the boundaries between adjacent segments (see section E.1 in Text S1) that the ratios of times spent on different segments, 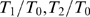, are invariant under any similarity transformation. In other words, they are invariant under the scaling of Euclidian length with a scale factor 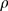. A consequence of this assumption is the following (see section E.1 in Text S1): Suppose 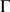 is cut into two parts 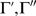, then there are three non-negative constants 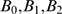, depending only on 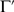 which are invariant under similarity transformations of 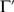, such that 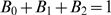, and
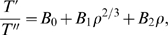
(13)where 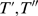 mark the times spent on 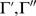, respectively, and 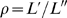 is the ratio of similarity between 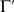 and 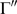.

### Experimental tests

The data used to test our hypotheses were derived from recorded hand movements and locomotion generated along *a priori* prescribed curves. We aimed to test the compatibility of the temporal properties of the movements with respect to two main principles: 1) geometrical invariance determines movement duration, 2) The mixing of different geometries can account for movement segmentation.

Three different tests were conducted. The first, using elliptical hand trajectories, tested whether an alternation between Euclidian and affine geometries better explains the experimentally observed relation between Euclidian curvature and velocity than describing the whole elliptical movement as obeying a single power law with constant exponent. This test also examined the limitations of the validity of the isochrony principle by investigating the relation between total duration and perimeter and the relation between the enclosed area and gain factor. The second test used trajectories generated by human subjects while tracking geometrically prescribed complex figural forms - cloverleaves, lemniscates and limaçons - during both drawing and locomotion. This experiment tested whether the proposed tensorial formulae (8), (9) can successfully account for the experimentally observed movement durations. We also examined whether it is possible to distinguish between movement duration during drawing and locomotion based on the different degrees of influence of the different geometries, i.e. whether both tasks are based on similar principles of invariance but arise from different mixtures of geometrical invariance. The third test, applied to the same data as the second test, examined the validity of equation (13) with respect to the ratios between the durations of the movements along the large versus the small loops of the lemniscates and limaçons. It also aimed at confirming the differences between drawing and locomotion identified by the second test with respect to the influence of the different geometries on the durations of movement along large versus small segments.

#### First test: Elliptical trajectories

Elliptical trajectories with different eccentricities, perimeters and performed under various speed conditions were recorded from three subjects (see section Methods and section B.1 in Text S1). For ellipses, affine and equi-affine parameterizations are proportional to each other; the scaling factor assures that full affine geometry predicts a strict global isochrony, while equi-affine geometry does not.

Full isochrony predicts the following linear relation between the velocity gain factor 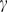 and the area of the ellipse 
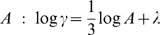
 (see section B.2 in Text S1). Here and elsewhere 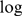 refers to a logarithm on the basis 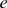. A linear regression conducted separately for each subject and speed condition confirmed a positive correlation between 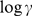 and 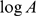 but with a slope constantly smaller than 1/3 for two subjects (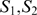) and almost equal to 1/3 for subject 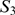 who was always faster than the other two subjects (see Table 1 and Figure S1). Subject *S*_3_'s movements also tended to show full isochrony (see below).

**Table 1:** Statistical analysis of elliptic drawings (1).

Speed	Subject	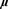	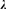	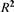
Slow	S1	0.12	−1.56	0.46
	S2	0.17	−1.30	0.91
	S3	0.28	0.29	0.93
Natural	S1	0.14	−0.78	0.45
	S2	0.23	−0.35	0.87
	S3	0.33	1.01	0.96
Fast	S1	0.18	0.17	0.59
	S2	0.21	0.35	0.80
	S3	0.28	1.32	0.95

Log gain factor versus log area.

Linear regression: 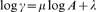, for each of the three speed conditions (Slow, Natural, Fast) and the three subjects (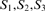). Presented are the best 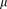 and 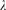, and the values of the coefficient of determination (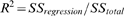) for the different ellipses. Figure S1 shows the data and the regression lines corresponding to table.

**Figure 1:**
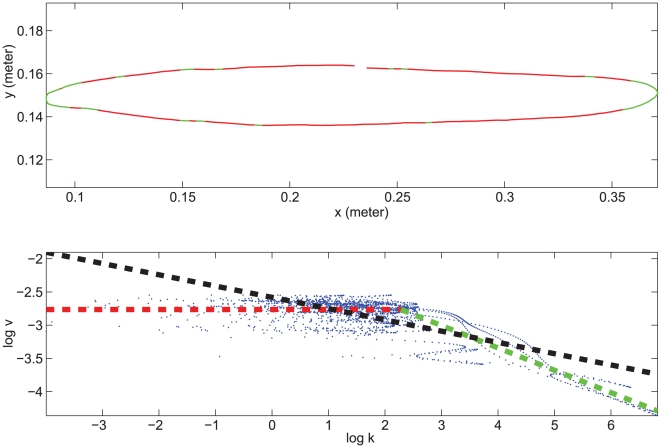
An example of elliptic segmentation: Comparing the piecewise linear law (PLL) of log 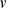 versus log 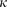, versus the regular Power Law, where 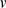 is the velocity and 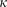 is the Euclidian curvature. Empirical values (blue) of the pairs 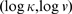 are compared to the piecewise regression lines of the PLL: 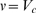 for 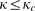 (red line), and 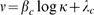 for 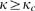 (green line), versus the regression line of the regular Power Law ( black). For all 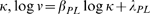. For this trajectory 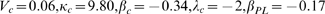, and 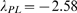.

If 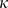 denotes the Euclidian curvature, the prediction based on equi-affine geometry is a linear relation 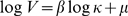, with 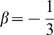. Such a relationship has been repeatedly described in the literature with the slope 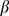 close to −1/3rd [12]. However, when analyzing the experimental pairs 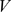 versus 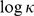, case by case, we did not find such a strictly linear relationship but, instead, a piecewise linear relationship comprising two segments: a horizontal segment for trajectory segments with small curvatures and another segment with a slope of 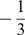 for segments with larger Euclidian curvatures (see Figure 1). Thus it appears that there are critical curvatures 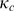 and critical speeds 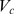, such that the Euclidian velocity is nearly constant for 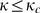 and 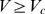. This is demonstrated by comparing the ratios of the sum of square errors (SSE) for the linear regression between 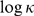 and 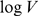 with a description consisting of two segments with slopes 0 and −1/3. We also calculated the probability that the piecewise linear model explains the data better than a single power law (see the legend of Table 2). These results are conclusive (see Table 2): the segmented representation captures the 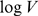 versus 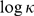 behavior better than a single power law.

**Table 2:** Statistical analysis of elliptic drawings (2): Log Velocity versus log Euclidian curvature, piecewise linear law (PLL) compared to the regular power law.

Eccentricity	Subject	Piecewise- Linear 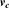	Piecewise- Linear 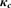	Power- Law 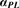	Power- Law 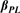	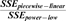	Probability
Small	S1	0.11	6.04	−2.23	−0.23	0.65	0.87
	S2	0.15	5.24	−2.12	−0.28	0.72	0.82
	S3	0.32	6.19	−1.31	−0.26	0.69	0.85
Medium	S1	0.19	16.09	−1.70	−0.14	0.95	0.58
	S2	0.19	13.10	−1.74	−0.17	0.85	0.72
	S3	0.49	9.28	−0.50	−0.28	0.89	0.68
Circle	S1	0.15	24.61	−1.98	−0.07	0.95	0.56
	S2	0.17	27.12	−1.99	−0.05	0.96	0.54
	S3	0.39	17.51	−0.68	−0.23	0.97	0.53

The PLL is: 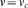 if 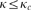, 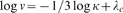 if 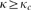; the regular Power Law is 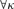, 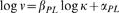.

For each of the three eccentricities and three subjects the table presents the best 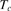 and 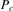 for the PLL and the best 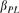 and 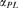 for the regular power law, plus the ratios of the sums of square errors (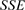) for the two linear regressions for the different ellipses. Also presented are the probabilities that the PLL model is better than the power law model, computed according to the equation: 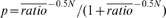 where 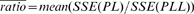 and 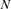 is the number of trials.

Our results thus confirm the existence of heterogeneous geometry and quantify a trajectory segmentation scheme compatible with the presence of separate equi-affine (or affine) and Euclidian segments during the generation of elliptical trajectories.

**Figure 2:**
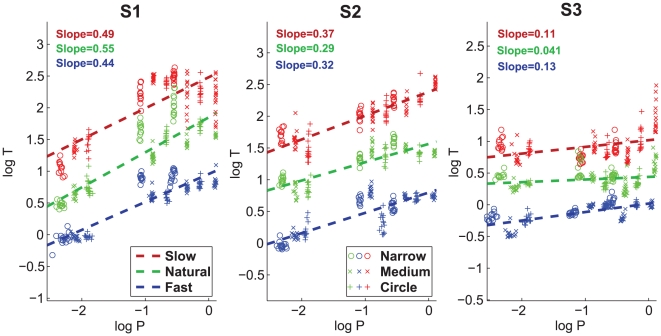
Experimental data of elliptic drawings and regressions. Log movement time (T) is plotted versus log perimeter (P). The regression lines between log T and Log P are shown for each subject 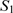, 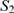 and 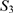 and each average speed condition (slow, natural and fast, red, green and black, respectively). Ellipses with different eccentricities are marked by different symbols (circle, cross and plus for narrow, medium eccentricity and circles, respectively). The regression parameters were calculated for all eccentricities together. The parameters of the regression lines are presented in Table 3.

To further investigate the influence of different geometrical representations on the movements, we used a linear regression analysis of 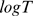 versus 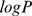 to examine the relationship between total movement time 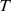 and the total perimeter 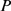 of the elliptical trajectories, (see Figure 2 and Table 3). For subjects 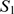 and 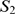, the slope was significantly greater than zero but smaller than 0.55 (for 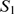, the slope ranged between 0.44 and 0.55 depending on movement speed, and for 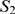 the slope range was 0.29–0.37). These slopes thus showed an imperfect tendency toward isochrony. For subject 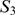 the slope was also positive but close to zero (0.041–0.13), very nearly showing full isochrony. The 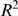 score in this case is problematic because it reports the extent to which the prediction is better than the mean of the data. When the slope is close to zero, the prediction is very close to the mean and the 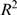 score is low.

**Table 3:** Statistical analysis of elliptic drawings (3): Log time versus log perimeter.

Subject	speed	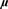	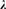	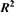
S1	Slow	0.49	2.48	0.62
	Natural	0.55	1.85	0.76
	Fast	0.44	0.96	0.80
S2	Slow	0.37	2.38	0.78
	Natural	0.29	1.56	0.71
	Fast	0.32	0.80	0.70
S3	Slow	0.11	1.02	0.14
	Natural	0.041	0.44	0.049
	Fast	0.13	0.02	0.5

The equation for the linear regression is: 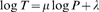. The best 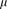 and 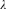 are presented for each of the three subjects and three speeds, as well as the goodness of fit of the data points to the linear regression expressed by: (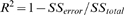). Note that the 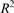 values assess how much the approximation of the data provided by the linear regression is better than the mean value of the data. For subject 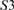 the 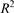 scores are low because the slopes (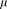) are close to zero. Hence, the linear approximations are not better than the mean values of the data points. For further details see Figure 2.

Figure 2 gives results from sets of repetitions of one experimental condition; total movement duration shows a tendency towards a larger variability than perimeter. However, closer examination of the data showed no correlation between the order of movement repetition and movement speed. The main conclusion we can draw from these data is the existence of a tendency towards isochrony which seems unaffected by speed. This tendency is strongly modulated by the specific strategy of each subject.

In sections B.2 and B.3 in Text S1, the relations between 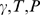 and eccentricity for elliptic trajectories are examined from the affine point of view and compared with the theoretical derivations in the studies by [15],[42] and with the experimental data from Viviani and Schneider [43]. In section C in Text S1 we also describe Viviani's [42] observation that the empirical law for the mean value of the velocity gain factor 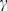 scales with the radius of the frame within which subjects produce continuous scribbling movements. We discuss the compatibility of these observations with affine geometry.

#### Second test: Complex figural forms, velocity predictions

We next examined a series of drawings of lemniscates, limaçons and cloverleaves (from [4]) and of locomotion trajectories along similar curves (from [17]). Thus we could compare the production of planar movements during two different motor tasks, drawing and locomotion. The drawings of cloverleaves were performed at three different speeds (marked by 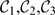 in the order of ascending speed), while in the locomotion experiment no instruction concerning movement speed was given. Subjects followed three lemniscate templates and three limaçon templates in the two experiments. The templates differed in the ratios between the perimeters of the two loops comprising these forms (see Figure S2), but the total length of the different forms was constant. (The lemniscates and limaçons were marked respectively as 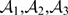 and 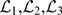 according to the ascending ratio of the sizes of the large versus the small loops). We then computed the Euclidian and equi-affine curvatures and derived the Euclidian velocity profiles corresponding to constant Euclidian, equi-affine and affine velocities, calling them respectively 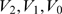 (see equations 7). We also constructed the velocity profile hypothesized by the geometrical mixture model (see below).

Several data sets were used in the computation of the different models and in the statistical tests. For drawing, all the data points were used. The velocity profiles of locomotion displayed oscillations due to the stepping movements. To eliminate these and to derive the subject's transport velocity during locomotion, we disregarded the velocity components due to the presence of steps. Thus, for locomotion we consider two data sets. One data set, the “stepwise sampled data set” (SSDS), consisted of the experimental data corresponding to time instants at which the body position (with respect to a point 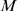 between the shoulders, see Figure S3) attained a local minimal height, corresponding to the end of a step. The second data set included all the position data for the M-point and was called the “complete sampled data set” (CSDS). The SSDS contained between 117 and 183 points, while the CSDS contained between 3310 and 6000 points per trial.

Then, based on the model for the mixture of geometries, hypothesized tangential velocity profiles were constructed using the following procedure (for further details see section Methods and section D.4 in Text S1):

1) By comparing the known experimental velocity and the three computed monotonic velocities (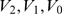, in logarithmic scale), we found segments of time during which a linear (barycenter) combination of the logarithms of the computed monotonic velocities approximated the logarithm of the experimental velocity with a very high degree of accuracy (up to or above 97%). The slopes (in 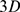) of the lines corresponded to the exponents 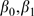 and 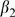 (whose total sum equals 1). Hence, the weights of the different geometries during those segments could be considered constant.

Altogether, seven techniques were used to find segments during which one or all three 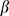 functions were constant. The first technique, i.e., that described above, enabled us to find the segments during which constant 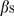
s could successfully approximate the data. Three other techniques assumed that one of the 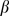
s is zero and searched for trajectory segments during which the other two 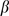
s were constant. Based on these segments and using a cubic spline interpolation for the functions 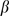
s, a predicted velocity was computed for the entire movement. The last three techniques started from the above interpolated velocities and searched for segments during which the 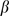 exponent, initially assumed to be zero, was constant. To all these segments we *a priori* added constraints in the vicinity of parabolic points (where we imposed 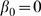) and at inflection points (where we imposed 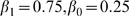 and 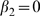). We refer to all the above segments as *special segments*. We emphasize that these segments were used in our derivation of the different 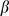 functions and do not necessarily correspond to motion units or segments.

2) For each scenario, we computed a smooth cubic spline interpolation of the 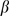 functions between all the special segments, yielding a theoretical velocity for the entire trial.

3) The values for the three remaining constants 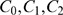 were chosen to be those giving the best match of the predicted trajectories to the experimental data.

4) We chose the best result among those constructed using the above seven scenarios. We call this velocity *the Geometrically Combined Velocity* (or *comb velocity*). For locomotion all the above computations were conducted using only the SSDS samples.

Note that we used the above algorithm, since at present, except for parabolic and inflection points, the model does not predict which geometrical combination should be realized for any given curve. However, we have tested the non-triviality of the model predictions using three different statistical measures: 1) Assessing the significance (or the statistical non-triviality) of the existence of special segments using an F-test (details are given below and in section Methods ); 2) Considering the number of fitted parameters and comparing a penalized score calculated for our models versus the corresponding score calculated for the constrained minimum-jerk model [44]. For this comparison we used the standard Akaike criterion 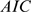 [45]. It was necessary to use such a criterion instead of simply using the 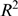 values, since our model uses many more parameters than the minimum-jerk model - around 23 vs. a single parameter corresponding to the total movement duration. 3) We calculated the coefficient of determination, 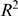, for our model to evaluate how well the model accounts for the data. Recall that our aim here, to examine whether movement timing and velocity are best explained by a combination of geometries, cannot be rejected based on the analysis of the experimental data.

First we refer to the non-triviality of the special segments found by the data analysis. The SSDS data were considered for the locomotion task. For all trials we computed the squared distance 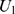 between the model and the experimental velocity, restricted to the union of all 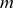 special segments and the total variance 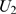 of the experimental velocity. The difference 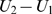 represents the variance of the experimental data during parts of the trajectory outside the special segments plus the residual variance on the special segments (See section Methods).

Considering the respective numbers of statistical degrees of freedom, we applied a Fisher test to the ratio 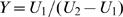. We also verified, as follows, that the variance of the experimental velocity with respect to the prescribed geometrical curves cannot itself explain the presence of special segments. We repeated the above computation replacing the variance of the experimental velocity with the squared distance of this velocity from the best trigonometric (i.e. Fourier) approximation of degree four. That is, we used four harmonics (hence with 9 real arbitrary coefficients for the 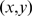 set of coordinates). See section Methods for more details of this procedure and Table 4 for the results. For both types of computation, the results confirmed that most of the special segments could not be explained by the intrinsic variance of the trajectories. For the drawing data, 61 of 78 trials (78%) satisfied the test involving trigonometric approximation. For the locomotion data, 65 of 91 trials (71%) satisfied this test.

**Table 4:** Results of the tests for statistical significance of the presence of intervals.

Exp	Shape	Test 1: % Significance	Test 2: % Significance
Drawing	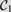	100%	77.78%
	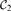	100%	100%
	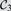	100%	66.67%
	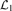	100%	83.33%
	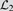	100%	66.67%
	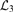	100%	55.56%
	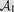	100%	100%
	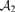	100%	100%
	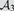	100%	100%
Locomotion	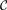	100%	75.86%
	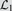	100%	62.07%
	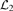	100%	60%
	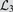	100%	63.33%
	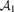	100%	83.33%
	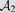	96.67%	96.67%
	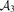	100%	92.59%

Column *Test 1:% significance* shows the percentage of trials for which the existence of special segments was found to be statistically significant. This was determined by comparing the success in matching the empirical velocities either with the model for the combination of velocities or their approximation using the mean velocity values. The analysis was performed in the log velocity space.

Column *Test 2:% significance* shows the percentage of trials for which the existence of special segments was found to be statistically significant. Here the success in matching the empirical velocities achieved by the model of the combination of geometries was compared with that using the trigonometric approximation. The analysis was performed in the log velocity space.

We also calculated the Akaike Information scores 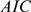 for the four models (combination, minimum-jerk, constant equi-affine and constant affine velocities) for the drawing and locomotion data (cf. section Methods). Because data sets composed of 5 samples were necessary in each elementary computation of velocity, the number 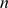 of statistically independent degrees of freedom equalled the total number of points divided by 5. The calculations were performed on both the drawing data and the CSDS of locomotion. We then computed the probability that our combination model was better than the minimum-jerk model (according to the equation 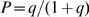, where 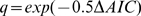, (for more details regarding this equation see [45]). Remember that higher 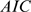 scores correspond to a worse result, thus a higher probability indicates a good model.

For locomotion, we examined the success of the four models by computing two sets of AIC scores for the velocity profiles predicted by these models. One set of scores was derived by applying the models to the SSDS data points (“SSDS-scores”), while the second set of scores was derived using the CSDS (“CSDS-scores”). The main difference between the AIC scores calculated for the two data sets is that for CSDS, the experimental velocity profiles contained velocity components due to stepping that are not accounted for by the combination of geometries nor by the minimum-jerk models. The CSDS data set contained more samples than the SSDS data set. For SSDS, the number 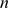 of statistically independent degrees of freedom ranged between 117 and 183, while for the CSDS this number, 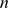, ranged between 662( = 3310/5) and 1200( = 6000/5).

**Figure 3:**
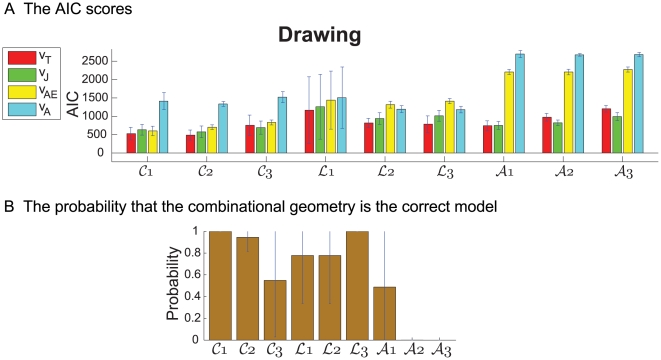
Drawing data: 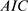 scores for the 4 models and for the different figural forms. (A) Akaike's Information Criterion (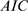) scores averaged across subjects and repetitions for the 4 models for all drawn shapes. Also shown are the standard deviations (SDs)of the AIC scores. The lower the 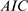 score is, the better is the model. Red bars show the scores of the model of the combination of geometries (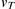); green bars, scores of the constrained minimum jerk model (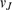); yellow bars, the constant equi-affine speed model (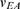); cyan bars, scores of the constant affine velocity model (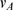). (B) Brown bars show the average probabilities (averaged across subjects and repetitions) that the combined model is better than the minimum-jerk model for the different shapes. Standard deviations are also shown. The probabilities were calculated according to the equation 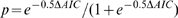, where 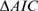 is the differences in scores between the two models. In both figure panels, the cloverleaves are marked by 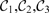 in the order of ascending speed. The limaçon and the lemniscate are marked by 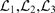 and by 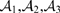, respectively, according to the ascending ratios of perimeters of the large to the small loops.

For the drawing data, the AIC scores for the four different models are shown in Figure 3A. The probability scores that the combined velocity model is better than the minimum jerk model are shown in Figure 3B. For drawing cloverleaves at slow and normal speeds, the combination velocity model had lower AIC scores than the other three models. As shown in Figure 3 it is conclusively better than the minimum-jerk model and its probability of being the correct model was larger than 0.85. When the cloverleaves were drawn at high speeds, the 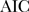 score for the minimum-jerk model was a little lower than for the combined velocity model, the probability for the combined velocity model being the correct model was around 0.5.

The 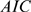 scores for drawing limaçons favored the comb velocity model over all other models, resulting in a probability larger than 0.7 that our model is always better than the minimum-jerk model. The 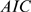 score for the minimum-jerk model for drawing asymmetrical lemniscates was less than the 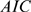 score for the comb velocity model, resulting in a probability of 0.4–0.5 that the comb velocity model is the correct one for 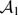 and very small for 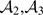.

**Figure 4:**
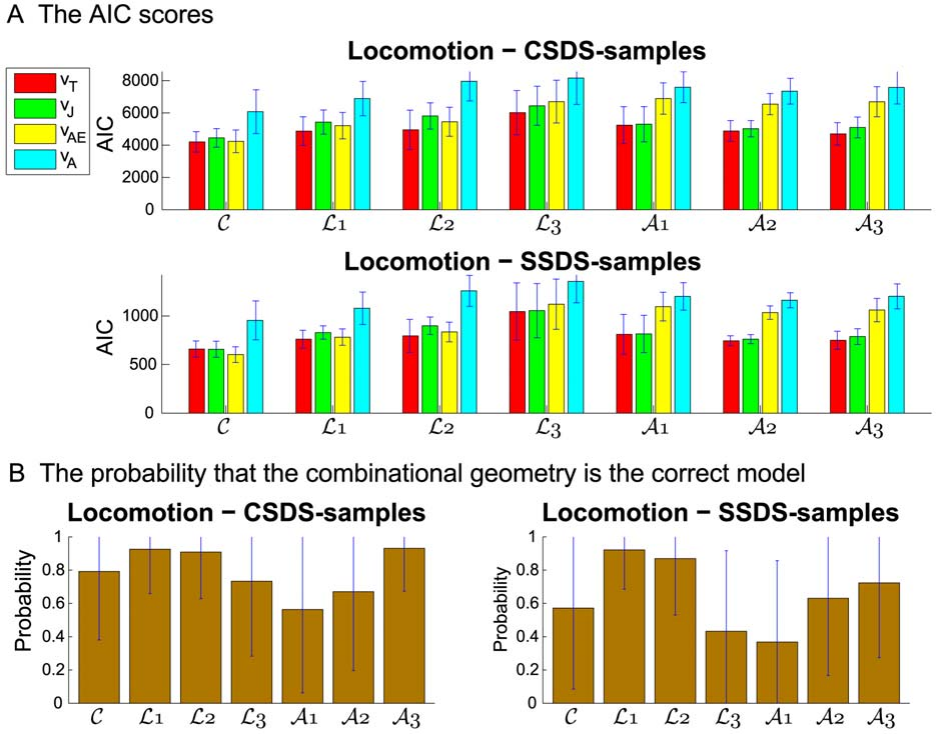
Locomotion data: 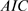 scores for the 4 models and for the different figural forms. (A) Akaike's Information Criterion (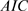) scores, averaged across subjects and repetitions, for the 4 models for all the shapes of the locomotion data. SDs of the 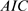 scores are also shown. The figure in the upper row in panel (A) shows the 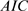 scores for the 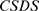 (all data), while the figure in the lower row in panel (A) shows the 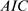 scores for the 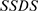 data. The lower the 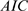 score is, the better is the model. Red bars show the scores of the combination of geometries model (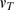); green bars, scores of the constrained minimum-jerk model (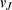); yellow bars, the constant equi-affine speed model (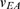); cyan bars, the scores of the constant affine velocity model (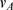). (B) Brown bars show the average probabilities (averaged across subjects and repetitions) that the combined model is better than the minimum-jerk model for the different shapes. SDs are also shown. The probabilities were calculated according to the equation 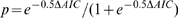, where 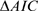 is the differences in scores between the two models. The figure on the left (panel (B)) shows the results for the 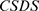, while panel (B) on the right shows the results for the 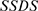. In both figure panels, the cloverleaves are marked by 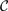. The limaçon and the lemniscate are marked by 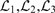 and by 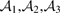, respectively, according to the ascending ratios of perimeters of the large to the small loops.

For the locomotion data (both the SSDS and the CSDS), the AIC scores for the four different models are shown in Figure 4A. The probability scores for both the SSDS and the CSDS that the combined velocity model is better than the minimum jerk model are shown in Figure 4B. For locomotion along the cloverleaf form, the 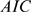 score for the SSDS (SSDS-scores) favored the equi-affine model, and the minimum-jerk model seemed to be better than the combined velocity model. In contrast, using the CSDS data set (CSDS-scores), the 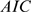 score favored the combined velocity model and the equi-affine model alone was worse than the combined velocity model but better than the minimum-jerk model. For locomotion along limaçons, the AIC score was lower for the combined velocity model than for the equi-affine and affine models. The differences in the AIC scores between the minimum-jerk model and the comb velocity model depended on the size of the small loop (for both SSDS- and CSDS-scores). The AIC scores indicated that the combined velocity model was better than the minimum-jerk model for 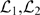 but not for 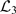. For locomotion along lemniscates, the SSDS-scores indicated that our model was more probable than the minimum-jerk model for 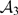, but less probable for 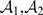. Using the CSDS-scores the combined velocity model was always better but its advantage was clear only for 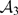.

Taken together, the probability that the combined velocity model is better than the minimum-jerk model for drawing was greater than 0.9 for 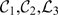, greater than 0.6 for 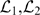 and greater than 0.5 for 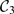. For locomotion, the CSDS-score showed that the probability that the combined velocity model was better than the minimum-jerk model was greater than 0.9 for 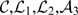, greater than 0.6 for 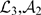 and greater than 0.5 for 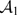.

In conclusion, for most of the drawing as well as locomotion trajectories, the minimum-jerk and the combined velocity models obtained the best 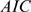 scores. There was a difference, though small, in the 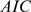 scores for the combined velocity versus the minimum-jerk models. This difference favored the combined velocity model especially for the locomotion data.

We computed the coefficients of determination, 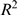, for all the models considered here. The 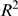 scores indicate how well the variance of the experimental velocity is explained by the theoretical predictions. Below, we use the 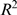 score for the SSDS locomotion data set which ignores the oscillations in position due to stepping.

**Figure 5:**
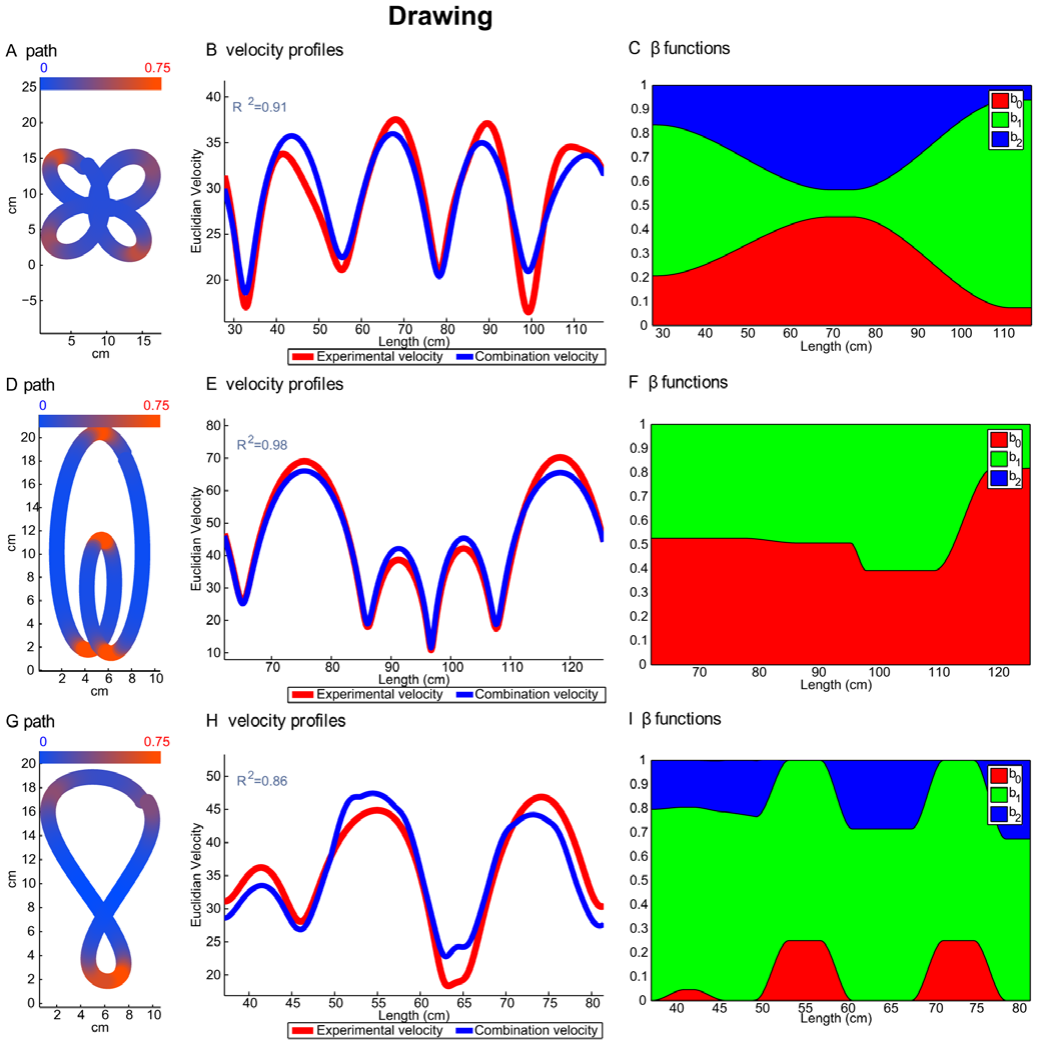
Examples from the drawing experiment. Every row shows an example of the second repetition of a drawing trial. First row, drawing of a cloverleaf; second row, drawing of an oblate limaçon; third row, drawing of an asymmetric lemniscate. Panels (A), (D) and (G) show the paths drawn by the subject. The colors marked on the paths represent the Euclidian curvature. Blue segments have relatively low curvature (∼0), red segments have a higher curvature (∼0.75). Color scale is shown at the top of the panel. Panels (B), (E) and (H) show the velocity profiles of the drawing. Red, experimental velocity profile; blue, velocity profile predicted by the model of the combination of geometries. Panels (C), (F) and (I) show values of the 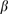 functions. Red area, value of the 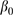 function; green area value of the 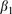 function; blue area, value of the 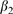 function. The values are aggregated one above the other such that their sum equals 1.

**Figure 6:**
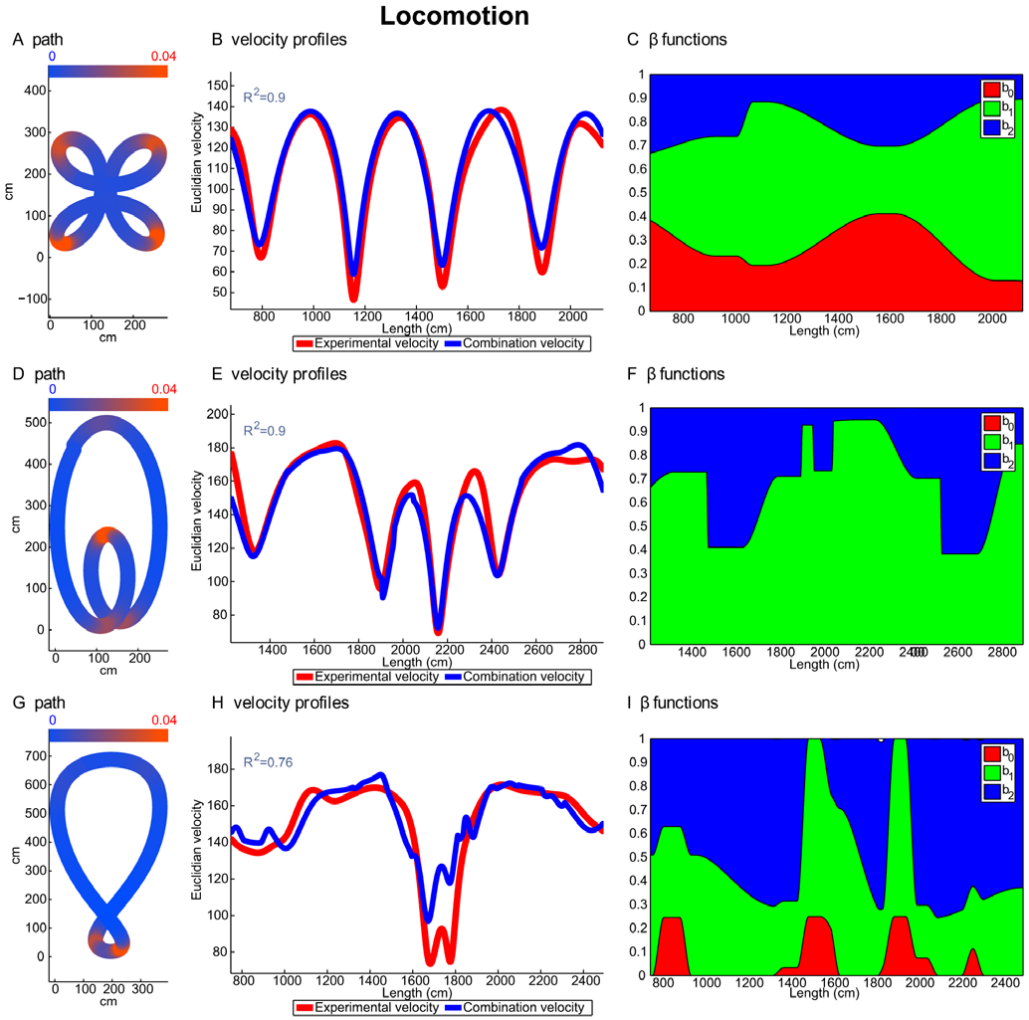
Examples from the locomotion experiments. Every row shows an example of the second repetition of a locomotion trial. First row, walking around a cloverleaf. Second row, walking along an oblate limaçon. Third row, walking around an asymmetric lemniscate. Panels (A), (D) and (G) show the paths drawn by the subject. The colors on the paths represent the Euclidian curvature; Blue, segments with a relatively low curvature (∼0); red, segments with a higher curvature (∼0.75). Color scale is shown in the panel. Panels (B), (E) and (H), the velocity profiles of the drawing. Red, experimental velocity profile; blue, the velocity profile predicted by the model of the combination of geometries. Panels (C), (F) and (I) show values of the 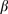 functions. Red area, value of the 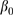 function; green area, value of the 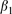 function; blue area, value of the 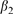 function. The values are aggregated one above the other such that their sum equals 1.

Figures 5 and 6 show comparisons of theoretically predicted versus experimentally recorded velocity profiles for drawing and locomotion. Figures 5A–C and 6A–C show examples of a slowly drawn and a walked cloverleaf, respectively. Examples of drawing and walking along the limaçon form are shown in Figure 5D–F and Figure 6D–F, respectively, while Figure 5G–I and Figure 6G–I show one repetition of the lemniscate form, for drawing and locomotion, respectively. Figures 5A, 5D, 5G, 6A, 6D and 6G display the movement paths and Euclidian curvatures. Segments of the curves marked in red represent parts of the curve with high Euclidian curvatures, while segments in blue represent parts with low Euclidian curvatures (see scales in the top parts of all these figures). Figures 5B, 5E, 5H, 6B, 6E and 6H also show the experimental velocity profiles of the recorded movements (red lines) and the corresponding combined velocity profiles (blue lines) for the three different figural forms. Further information and examples of the experimentally recorded paths and velocity profiles and their comparison to the velocities predicted by the combined velocity model are presented in Text S1 section D and in Figures S4 and S5 for the drawing and locomotion data, respectively.

**Figure 7:**
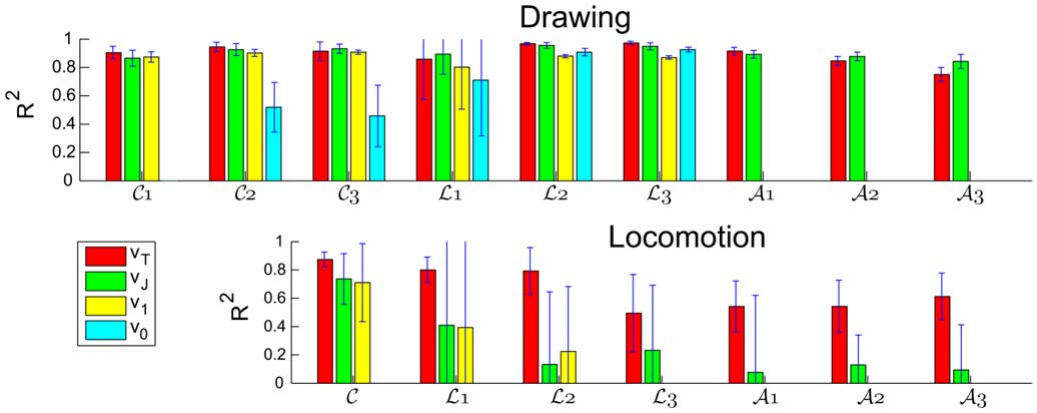
The 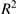 scores of the 4 models for the different figural forms. Summary of the 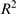 scores for all 4 models for all the figural forms for both drawing and locomotion data. The bars represent mean scores ±SD averaged over all subjects and trials. Red, score obtained for the model of the combination of geometries (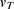); green, score of the constrained minimum-jerk model (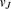); yellow, score for the constant equi-affine velocity model (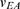); cyan, score for the constant affine velocity model (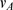). For the marking of the different forms see Figure 3.

The 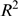 scores derived by comparing the experimentally recorded and theoretically predicted velocity profiles are displayed in the top left of these figures. The 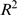 values were calculated for the entire set of movement trials, not only for the one run presented in the figures. Table 5 and Figure 7 give the mean and standard deviation values of the 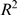 scores of the velocities for each figural form. The 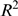 scores for all the different theoretical models are lower for locomotion than for drawing. This can be explained by the higher levels of noise in the locomotion data. However, based on both the 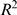 and the 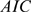 scores, the model that combined the three geometries gave a better approximation for locomotion than all other models.

**Table 5:** The 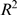 scores of the 4 models for the various figural forms.

Exp	Shape	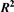 of Combination	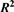 of Min-jerk	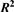 of EA	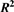 of A
Drawing	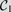	0.90±0.05	0.86±0.06	0.87±0.03	−0.11±0.69
	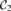	0.94±0.03	0.92±0.05	0.90±0.02	0.52±0.17
	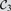	0.91±0.06	0.93±0.03	0.91±0.01	0.46±0.20
	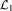	0.91±0.02	0.90±0.02	−2.87±0.36	−12.38±3.01
	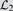	0.84±0.03	0.88±0.02	−2.82±0.70	−11.22±1.45
	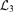	0.75±0.05	0.83±0.04	−3.12±0.85	−10.35±1.92
	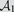	0.86±0.27	0.82±0.32	0.80±0.28	0.71±0.37
	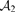	0.97±0.01	0.95±0.02	0.88±0.01	0.91±0.02
	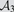	0.97±0.01	0.95±0.02	0.87±0.01	0.93±0.02
Locomotion	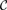	0.86±0.07	0.74±0.18	0.83±0.10	−4.27±8.38
	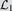	0.79±0.09	0.41±0.60	0.47±0.80	−8.09±13.26
	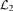	0.79±0.16	0.15±0.51	0.40±0.46	−12.56±11.16
	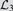	0.48±0.27	0.20±0.47	−0.44±1.22	−7.80±6.95
	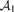	0.51±0.18	0.08±0.53	−8.22±5.15	−20.62±12.90
	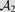	0.54±0.23	0.13±0.21	−7.31±3.22	−21.34±6.55
	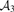	0.60±0.17	0.09±0.31	−6.51±2.23	−21.19±5.01

The means and SDs of the 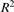 scores for the pure equi-affine and affine geometries, minimum-jerk and the combination of the Euclidian, equi-afine and affine geometries. Remark: the 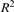 scores of non-linear functions can be negative. In this case we say that the mean value of the data points matches the data better than the values predicted by the tested model.

**Figure 8:**
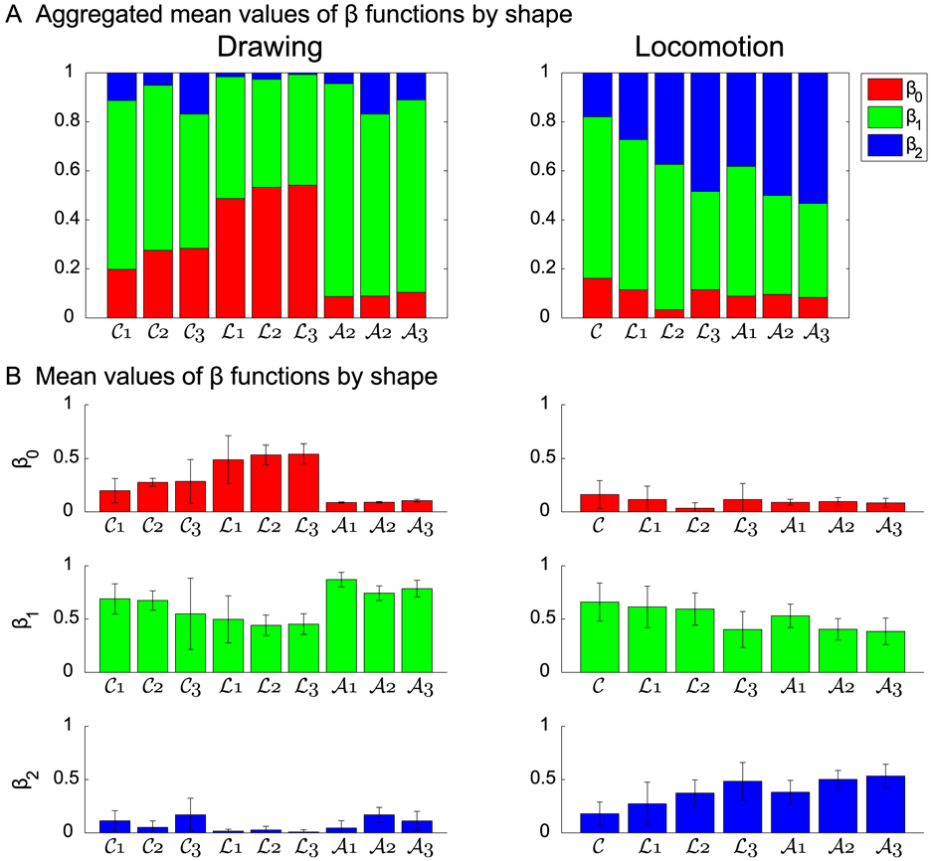
The mean values of the 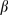 functions for the different figural forms. The mean values of the 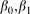 and 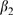 functions averaged over trials and subjects, summarized over the templates of the different figural forms. In panel (A) the values of the 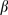 functions are aggregated and in panel (B) they are displayed separately with their corresponding SDs. For the marking of the different forms see Figure 3.

Table 6 and Figure 8 show the mean and standard deviation values of the *β*s for each figural form.

**Table 6:** The mean values of the 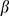 functions for the different figural forms.

Exp	Shape	mean of 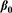	mean of 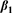	mean of 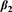
Drawing	cloverleafs	0.25±0.14	0.64±0.22	0.11±0.12
	Limaçons	0.52±0.14	0.46±0.14	0.02±0.03
	Lemniscates	0.09±0.01	0.79±0.09	0.12±0.09
Locomotion	cloverleafs	0.16±0.13	0.66±0.18	0.18±0.11
	Limaçons	0.08±0.11	0.56±0.19	0.36±0.18
	Lemniscates	0.09±0.04	0.43±0.13	0.48±0.12

The mean and SD values of the 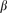 functions of the combinations. The function 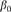 represents the influence of affine geometry in the combination of geometries. The function 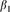 represents the influence of the equi-affine geometry in the combination of geometries. The function 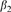 represents the influence of Euclidian geometry in the combination of geometries.

The average values of the 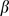 weight functions was correlated with the degree to which constant equi-affine and constant affine velocities alone explain the data.

To investigate movement segmentation we examined the influence of the various geometries on the different parts of the three figural forms. To this end, we inspected the values of the 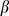 functions along movement repetitions of these curves (for examples, see Figures 5C, 5F, 5I, 6C, 6F and 6I). The red area represents the values of 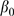, the green area represents the values of 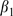, and the blue area represents the values of the 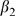. The sum of the 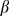 functions is always one. (Note that if, for example, *β*_0_ = 0.2, *β*_1_ = 0.5, and *β*_2_ = 0.3, the red area will appear between the abscissa and the line parallel to it passing through the value of 0.2, the green area will appear between that line and another line parallel to the abscissa through the value of 0.7, while the blue area will appear above the latter line). Figures S6 and S7 show further examples of the velocity profiles of the recorded movements together with values of the 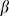 functions for drawing and locomotion trajectories, respectively. Figure S8 presents the color coding for the 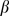 function values shown in Figures S6 and S7.

As can be seen from the drawing example of the limaçon, Euclidian geometry has no influence on the theoretically predicted velocity (there are no blue parts), while in the locomotion example there is no affine influence (no red part). In the lemniscate example, movement trajectories are not segmented at singularity points, as previously suggested in the literature [42]. Instead, the singularity points are embedded within the trajectory as demanded by the principle of the extension of singularities. These examples are typical only for the features we have mentioned. There is still considerable variance among the trials that needs further study.

Examining the different velocity profiles, 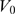, 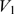 and 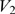 and comparing them to the experimental velocities, we see that most of the time more than one geometry is needed to construct the experimental velocity. In the examples shown in Figures S4B and S4E for the experimentally recorded hand shown in Figure S4A, the experimental velocity (red line) mostly lies between the affine and equi-affine velocities (the solid blue and black lines, respectively). The lemniscate are more complicated because of the presence of inflection and parabolic points. Still, in Figure S4H the experimental velocity follows the equi-affine velocity until it reaches an inflection point, where the net velocity is obtained by combining the affine and equi-affine velocities, thus allowing them to cancel each other out. A similar phenomena was found for the locomotion trajectories (see Figure S5).

We shall now inspect drawing and locomotion of each of these figural forms more closely. Here we mainly use 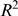 to compare different curves and conditions, rather than comparing the different models. We also display data for *β*s (see Figure 8). This figure shows that the affine contribution to the drawing movements is not negligible and that the limaçons' trajectories are more affine than those of the cloverleaves. These, in turn, are more affine than lemniscates. For walking, the influence (weight) of Euclidian geometry is non-negligible. Its influence is smaller for cloverleaves, but for lemniscates and limaçons its influence co-varies with the ratios of the size of the large versus the small loops.

For cloverleaves the 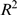 of the *comb velocities* was larger than 0.85 for all drawing trials with mean value 0.92. The equi-affine velocities gave similar scores (0.9±0.03 respectively). The affine velocity score, on the other hand, was low (0.49±0.18). Hence, the experimental velocity cannot be explained by the affine velocity alone. The locomotion data gave a greater difference between the *comb velocity* (0.87±0.05) and the equi-affine velocity (0.83±0.10) and the affine velocity received negative 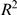 scores for most trials. (Remark: the 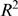 score for non-linear functions can be negative. In this case we say that the mean value of the data points matches the data better than the values predicted by the model for these data points.) These observations are confirmed by the 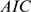 scores as shown in Figures 3 and 4. As can be expected from these results, the equi-affine velocity has a large influence on the combined velocity of the cloverleaves (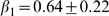 for drawing and 0.66±0.18 for locomotion, see Table 6). Comparing the means of the 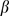 functions of the trials of drawing cloverleaves at medium speed with those of locomotion trials reveals a significant difference for the values of 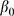 and for the values of 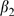. The drawing trials are more affine, while the locomotion trials are more Euclidian and both geometries are needed.

The limaçons gave high 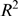 scores of the combined velocity, 0.93±0.16 for drawing and 0.69±0.24 for locomotion. Both the equi-affine and affine geometries had higher 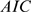 scores than the combined velocity but their absolute 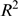 scores were still very high (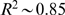) for the drawing trials. Thus, affine geometry is dominant in the combined velocity (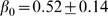). The influence of equi-affine geometry is also strong (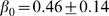), while the Euclidian influence is negligible. In contrast, the locomotion trials yielded negative 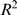 scores for the affine velocity. This is reflected in the small role that the affine geometry plays in the comb velocity (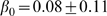) and the significant effect of Euclidian geometry (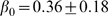).

The 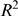 scores for the combined velocity model for lemniscates were 0.82±0.07 for drawing and 0.56±0.18 for locomotion. The influence of affine geometry was very weak for both drawing and locomotion, (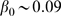). However, affine geometry is still important because it affects the velocity at inflection points preventing the equi-affine velocity from rising to infinity. In drawing lemniscates equi-affine geometry was very dominant (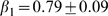), while in locomotion along lemniscates, the Euclidian and the equi-affine geometries had an equal influence.

**Figure 9:**
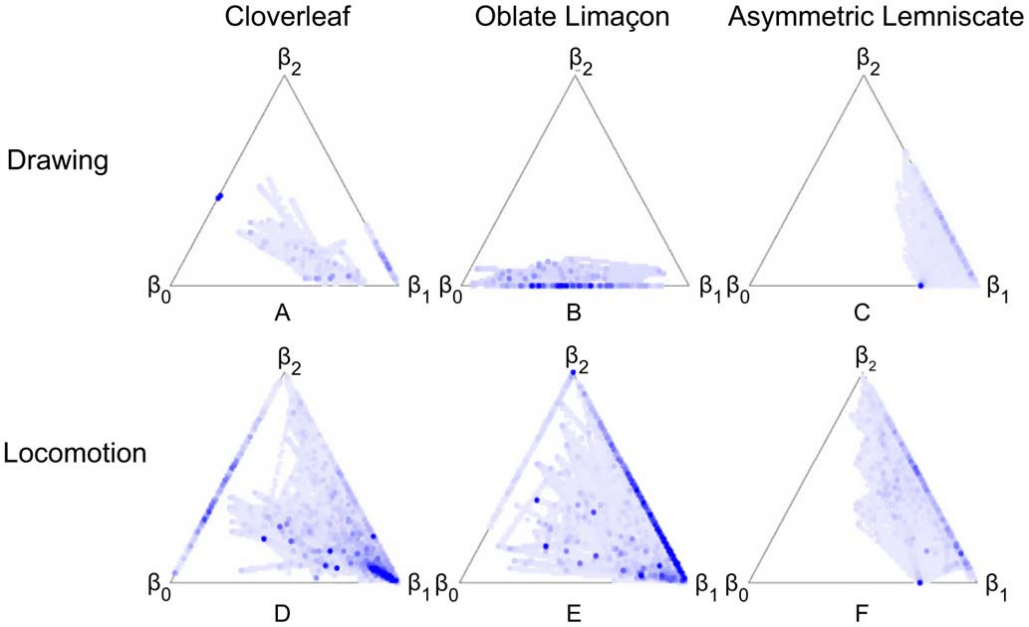
Representation of the values of the three 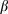 functions during the different trials. The distribution of the 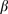 functions aggregated over all trials of the same figural form. A point within the triangle gives the values of the 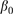, 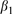 and 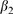 functions where 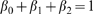. The values of 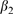 function for such a point are equal to the area delineated by the small triangle created by passing lines between this specific point and the two bottom vertices. The values of 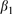 are equal to the area delineated by the small triangle created by passing lines between this specific point and the left bottom and top vertices. The values of 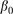 function are equal to the area delineated by the small triangle created be passing lines between this point and the right bottom and top vertices. For example, a point on the triangle's edge marked by 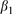 is a point where 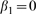. For a point located at the top vertex 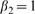 and 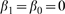. In the center of the triangle 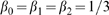. The color of any point within the large triangle indicates the number of times that that specific combination of 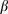 function values was found. A white point shows a combination that did not appear in any of the trials. A dark blue point represents a combination occurring many times. Panel (A) contains all the trials of the drawing of cloverleaves. Panel (B) contains all the trials of the drawing of oblate limaçon. Panel (C) contains all the trials of the drawing of asymmetric lemniscate. Panel (D) contains all the trials of the locomotion of cloverleaves. Panel (E) contains all the trials of the locomotion of oblate limaçon. Panel (F) contains all the trials of the locomotion of asymmetric lemniscate.

The distribution of values of the 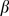 functions for the various trials are better observed using the six triangles shown in Figure 9. Each triangle represents data for all the trials of the same experiment and shape. Every point within the triangles represents a specific combination of 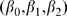. The color of each point indicates how frequently a particular combination appeared in the trials, with a dark point representing a frequent combination and a light point representing a rare combination. Areas within the triangles which contain no points represent combinations that were not used at all (see the figure legend for the correspondence of 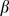 values to any given point within these triangles.)

Figure 9 shows that Euclidian and affine geometries contributed similarly during the cloverleaf trials. During drawing there is a tendency toward affine geometry. During the locomotion trials, as can be expected from the mean values of the 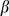 functions, there is a high tendency towards Euclidian geometry. However, in spite of this tendency, there are still many locomotion trials during which affine geometry showed a larger influence than Euclidian geometry. For the lemniscate trials, the influence of affine geometry can be seen mostly around the inflection point. For the locomotion data Euclidian geometry had a larger influence than equi-affine geometry. On the other hand, for the limaçon paths, during which there are only parabolic but no inflection points, drawing and locomotion were quite different. For drawing movements, mainly affine geometry created isochrony and an affine description alone provided a good explanation for the observed durations and velocities. In contrast, during locomotion, Euclidian geometry was twice as influential as affine geometry, but, again, as in the case of locomotion along cloverleaves, we found a large number of segments during which affine geometry played a larger role than Euclidian geometry.

In conclusion, the equi-affine geometry was almost always the dominant geometry. The mean value of 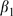 was greater than 0.45 for most of the drawing and locomotion trials. Euclidian geometry played an important role in locomotion for the three kinds of figural forms, whereas affine geometry played a small, though important role. The reverse was true for drawing (except for lemniscates): affine weights were important (i.e., the values of 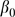 were 0.25, 0.52, 0.09 for cloverleaves, limaçons and lemniscates, respectively) while Euclidian weights (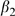) for the same shapes were 0.11, 0.02, 0.12, respectively. For detailed results of the 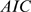 scores see Table S1 in Text S1.

#### Third test: Complex figural forms, drawing and walking

Using the data from [4] and [17], we analyzed the effect of the ratios between the large and small loops in the lemniscates and limaçon forms. The generated paths consisted of segments with different length ratios between a smaller and larger loop. This allowed us to test the validity of the prediction presented at the end of section sec∶quantitative laws for the ratios between the movement durations for the two loops (see section E in Text S1).

**Figure 10:**
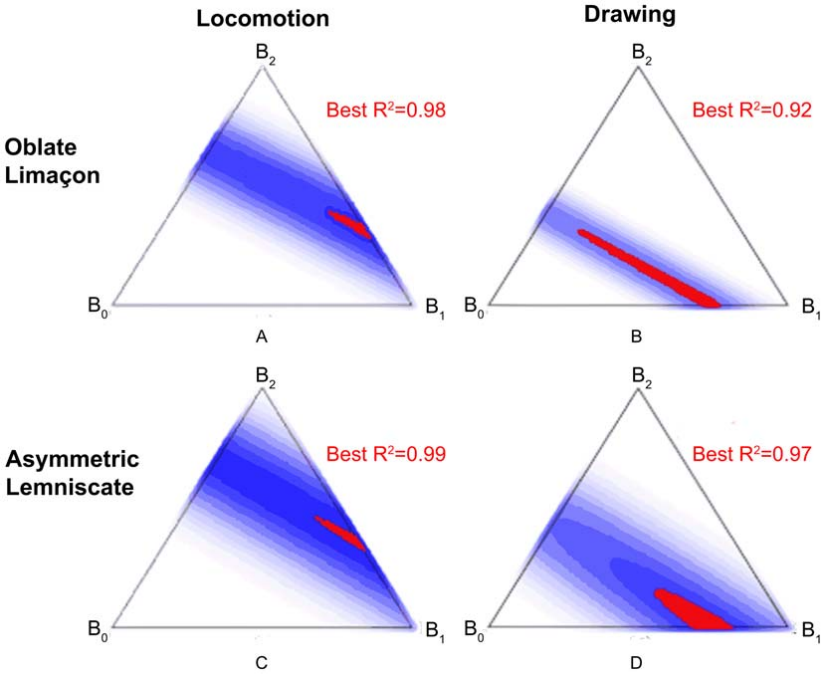
The 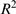 score for all the coefficient combinations for equation 13. The 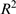 values of all the possible constant coefficients for the equation: 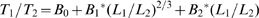. A point within a triangle describes the values of 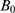, 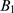 and 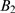 where 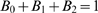. The values of 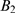 equal the area delineated by the small triangle created by passing lines between this specific point and the two bottom vertices, where the area of the large triangle is equal to 1. The values of 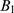 are equal to the area delineated by the small triangle created by passing lines between this specific point to the left bottom and top vertices. The values of 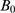 are equal to the area delineated by the small triangle created by passing lines between the point to the right bottom and top vertices. For example, at a point on the edge of the triangle marked by 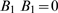. For a point located on the top vertex 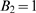 and 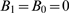. In the center of the triangle 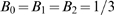. The color of a point represents the value of the 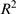 score for the corresponding combination of the 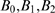 values; the darker the color, the higher the value of the 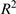 scores. The red points are those with the highest 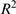 score. This value is given in red beside each triangle. Panel (A) contain the data of the locomotion of oblate limaçon. Panel (B) contain the data of the drawing of oblate limaçon. Panel (C) contain the data of the locomotion of asymmetric lemniscate. Panel (D) contain the data of the drawing of asymmetric lemniscate.

Both for drawing (3 subjects) and for locomotion (10 subjects), three different ratios 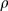 between the Euclidian lengths of the small and large loops were derived from the recorded movement data. To test equation (13), the triangle of barycenter positive coordinates 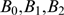, satisfying 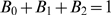, was divided into a lattice with a mesh size of 0.01. For each point in the lattice we calculated the value of the 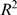 score assessing the success of the predicted ratios of durations to capture the experimental data (see Figure 10).

There were always regions within the triangle where 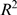 was high (more than 0.92), but these regions mostly consisted of parallel strips. Consequently, only a fixed linear combination of 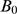 and 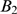 (generally close to *B*_2_−*B*_0_ = some constant) gave high 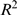 scores. The red part in Figure 10 represents the area with the highest 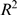 values. Only these areas corresponded to specific (large) values of 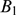 (except for drawing limaçons, for which the region always remains a strip, as in the triangle in Figure 10A). The main result is a clear difference between drawing and locomotion: for drawing 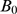 is larger than 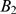. That is, the strip of high 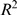 lies under the altitude through the 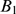 vertex. The reverse holds for locomotion, where 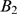 is larger than 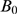. This again demonstrates that, during drawing, affine geometry has a stronger influence on the ratios between the time spent moving along the two loops. That is, from equation (13), the larger 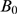 is, the closer we come to full isochrony. During locomotion the ratios between the durations is similar to the ratios between the perimeters of the large versus the small loops.

A second type of statistical analysis was performed over the same three constants 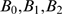 and the exponent 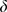 such that, for each 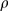,
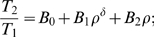
(14)The purpose of this analysis was to find the values of 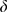 for which the highest 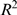 could be obtained. The results demonstrated that the interval of *δ*'s giving the highest 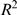 scores contained the value 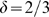. The differences in the degree of influence of the various geometries between drawing and locomotion persisted for different values of 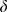.

**Figure 11:**
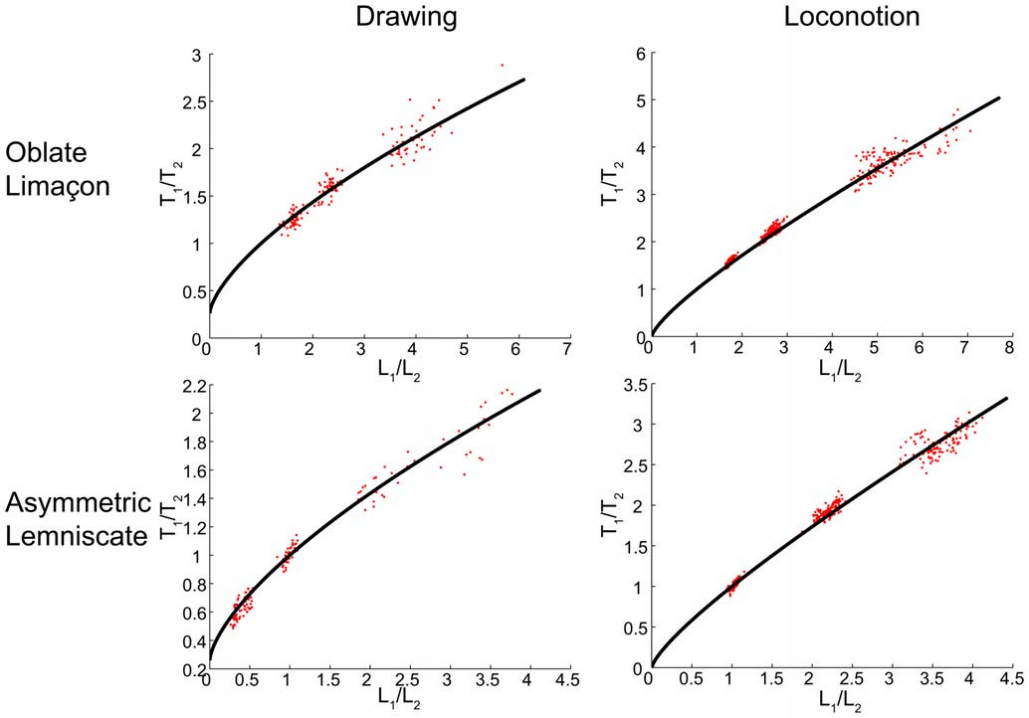
Examples of the experimentally measured ratios of movement durations versus the experimentally measured ratios of Euclidian lengths. The red dots represent the experimentally measured ratios of movement durations 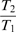 versus experimentally measured ratios of movement lengths 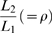. The black line represents the function 
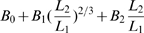
 for one of the set of constant *B*-s in the region of high 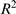 scores.

Figure 11 compares the experimentally measured ratios of movement durations with those predicted by equation (13). It also gives 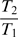 versus 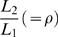 for one of the set of 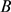 constants in the region of high 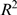 scores for both locomotion and drawing.

## Discussion

### A new theory: the timing of voluntary movement trajectories arises from a combination of several geometries

We present a new theory explaining how the timing of voluntary movements changes according to path geometry. Our model proposes that the velocity along the path is a specific composition of different canonical velocities, a composition that may vary among different segments of the same movement. Both geometrical invariance and movement segmentation are consequences of this principle. The canonical velocities to be combined depend only on path geometry and are defined within three major 2D-spaces: equi-affine, Euclidean and affine spaces. Equi-affine geometry is associated with a measure of area, Euclidian geometry with a measure of distance and affine geometry with the notion of parallelism.

The above notions are illustrated in our analysis of elliptical hand drawings. The trajectories contained two types of curved segments, each displaying different relationships between instantaneous velocity and Euclidian curvature and corresponding either to affine or Euclidian geometries. The Euclidian segments were those portions of the trajectory during which the Euclidian curvature was rather low - below some threshold - while the affine segments corresponded to the more curved portions. Such a description of segments accounts for the observed behavior better than a description based on a single power law (the probability of providing a better model than a single power law model was always higher than 0.64 and up to 0.97 for small ellipses). The observed behavior is a compromise between constant ordinary Euclidian speed and the isochrony principle, which reflects the effect of full affine geometry on motor timing.

For drawing the three figural forms studied here (cloverleaves, lemniscates and limaçons), the comparison of the three canonical velocities with the corresponding experimentally recorded ones strongly supports our new theory. The predicted purely equi-affine and the experimentally recorded velocities were very close for 70% of the time. The disagreement during the remaining 30%, could be systematically explained: here the velocity departed from entirely equi-affine and varied in a direction indicated by full affine or Euclidian velocities, as shown by the velocity profiles (see Figures S4 and S5). For instance, for drawing limaçons the difference between the actual and equi-affine velocities showed a tendency towards the full affine velocity, while during locomotion along similar curves, the discrepancy between the actual and equi-affine velocities suggested an influence of the constant Euclidian velocity (see Figures S4E and S5E).

To quantify these relations, we analyzed the experimentally recorded trajectories of human drawing or walking along the prescribed figural forms mentioned above. More than 90% of the velocity variance of drawing movements and 60% of the velocity variance for walking (based on the 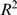 measure) was explained by the combination of several geometries. We also showed that for locomotion our model provides more information on motor timing than the constrained minimum-jerk model. For drawing, our model is only slightly better than the minimum-jerk model and both models are excellent. Given that the minimum-jerk model has no adapted parameters and that, in contrast, our model of geometrical mixture involves the selection of up to 30 parameters for each trajectory, it was necessary to compare these models using a standard penalty score such as the AIC. Our model generally remained more successful in accounting for the data than the minimum-jerk model (see Figures 3 and 4).

At first sight, the flexibility offered by three different geometries seems so large that one could imagine that such a model would produce a good fit for any possible movement data set. It was therefore important for us to ask in what way are our results non-trivial? Firstly, the prediction that speed is a weighted product of all three canonical velocities is non-trivial since *a priori* the observed speed could have exceeded the envelope corresponding to a linear combination of the logarithmic functions of these velocities, obtained while the total sum of their weights is precisely equal to 1.0. Secondly, we verified that the existence of several segments, during which constant combinations of the canonical velocities could account for the observed velocity, is unlikely due only to chance. Thirdly, we have shown that considering the number of free parameters and accordingly using the AIC scores, which appropriately quantify adapted measures of goodness of fit, our model successfully accounted for the observed data. Hence, based on these arguments, our principal result can be formulated as follows. The tensorial combination of canonical invariant parameters gives rise to statistically non-trivial predictions which were not rejected by the data against which they were examined. Moreover, this is a relatively simple model, which is grounded on a general point of view about the brain's mode of functioning.

The new point of view provided by the geometrical combination of velocities permits us to demonstrate several characteristics of motor timing.

First, we demonstrated that the global shape and size of the trajectory essentially influence motion timing (Figure 8). For instance, when drawing the three limaçons, subjects used affine geometry (responsible for isochrony) more than when drawing cloverleaves and even more so than when drawing lemniscates. However, on average, the influence of the ratios of the sizes of the large versus the small loops on the full affine weight was negligible. In contrast, during locomotion, it is remarkable that the Euclidian weight grew linearly with the ratios of the size of the large versus the small loops. Since the total perimeter remained constant, a decrease in the size of the smaller loop resulted in increasing the size of the larger loop. Thus, a possible explanation of the growth of 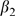 is that low Euclidian curvature without a change in convexity makes Euclidian geometry dominant for locomotion (as we verified directly for ellipses during drawing). Note that for lemniscates, based on the theoretical study of singularities, we imposed 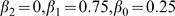 exactly at the inflection points. The good agreement achieved with the experimental data confirms this hypothesis.

Second, we discovered that the main difference between drawing and locomotion was the opposite degree of influence of full affine versus Euclidian geometries. For drawing, 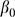 was important and varied between 0.1 and 0.6, while for locomotion it was 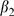 that varied between 0.2 and 0.6 and hence was more important than 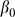. Possible reasons for these differences are differences in the control strategies used, or the existence of different biomechanical constraints.

We also applied a more restricted idea of segmentation by studying the effect of alternating between different dominant geometries. As a first approximation we assumed that segments with a constant velocity only within one specified geometry can successfully account for the observed ratios of time spent moving along the large versus the small loops of the complex figural forms as a function of their respective sizes. The observed ratios of movement durations have also corroborated and provided further evidence for our finding that the net balance between Euclidian and affine geometries is totally reversed for drawing versus locomotion.

All these results confirmed our expectation that affine geometry is significant in a theory of movement timing. The canonical velocity of full affine geometry yields the same total time spent on a curve and on the curve obtained through any dilatation. That is, the dominance of affine geometry here corresponds to isochrony, even though it is imperfect. However, we found experimentally that the pure affine geometrical arc-length is generally only a secondary component in determining movement timing, although it is always present during drawing movements. The notable exception is hand drawing of limaçons, where full affine canonical velocity alone explains the entire timing pattern very well. These results point to the important role of equi-affine geometry in motor timing.

### The importance of equi-affine geometry

The equivalence of the 2/3rd power to moving at a constant equi-affine speed [8] expresses the dominance of equi-affine geometry in trajectory planning. It has recently been found that 3D human drawing movements also tend to be generated at a constant equi-affine speed [46],[47]. How does this dominance of equi-affine geometry arise?

A first possibility is that the equi-affine invariant parameter may be computationally simpler. This invariant parameter is of third order, i.e., the order of the variation of acceleration, namely jerk. It could be coded by proprioceptive or vestibular information especially during locomotion [48],[49]. In contrast, full affine invariant parametrization is of the fifth order. Moreover, the equi-affine subgroup of transformations is uniquely defined by the full affine group, even without fixing a unit of area. Thus, affine invariance can be canonically broken into equi-affine invariance, thus simplifying the necessary computations.

A second possibility is the probable importance of area perception during motion, and we know that equi-affine transformations preserve areas. The amplitude of a piece of motion can be judged from the area enclosed by the corresponding segment of the trajectory and by the straight segment joining its initial and final positions. As we have seen in sec∶quantitative laws (see also E in Text S1), for monotonic equi-affine trajectories, the ratio of the total times spent along trajectory segments is a function of the ratios between the above enclosed areas.

A third explanation may correspond to the link between equi-affine invariance, the optimization of smoothness and the minimum jerk principle (cf. [4],[50]) or the minimum variance principle, both giving similar results. Todorov and Jordan [44] observed that the 2/3 law is equivalent to nullifying the normal component of the instantaneous jerk. Related to this is the attractive power of parabolic points and parabolic segments (see [51],[52]), because parabolic segments, for which equi-affine geometry is the only possibility, minimize jerk, obey the 2/3 power law and are invariant under equi-affine transformations. This link between the 2/3 power law and the minimum-jerk model may also be the root for the agreement between these principles in explaining motion timing from path geometry.

The fourth explanation for the dominance of equi-affine geometry is based on dimensional analysis which provides a completely different direction of support for the 2/3 law. Let us suppose that during motion, the total variation of energy 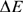 over each unit of time 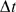 and mass 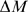 is equal to a constant 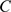 for all the segments of the trajectory. This constant has the dimension 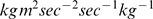, where 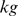, 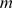 and 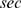 mark the units of mass (kg), length (meters) and time (seconds), respectively. From the constancy of 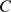, the dimension of time 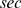 then becomes equivalent to 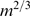 and the dimension of the resulting velocity is 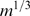. For turbulent fluid flows this is the well-known relation of Kolmogorov and Oboukhov between the length scale and the mean variation of velocity (see [53],[54]). Note that the 2/3 law for movement duration only refers to the radius of curvature, similarly to the radius of a vortex in the Kolmogorov-Oboukhov law.

All the above explanations for the dominance of equi-affine geometry in movement timing arise from some sort of invariance. However, in the framework of our theory, it is natural to propose that the main reason for the dominance of the equi-affine geometry (and consequently the 2/3 law) is that it offers an excellent compromise between full affine invariance and the reduction of computational complexity.

### Timing and the unity of action

We propose that movement duration is determined by invariance and computation. In the present framework, movement duration is related to space. This agrees well with Piaget's [55] suggestions about the development of children's conception of time: “The psychological interpretation of temporal notions … is that time forms a coordination of movements of different speeds.” The production of time jointly with geometrical form also agrees well with hypotheses on the neural basis of temporal processing, see e.g. Mauk and Buanomano [56], stating that “… given the intricate link between temporal and spatial information in most sensory and motor tasks, timing and spatial processing are intrinsic properties of neural function, and specialized mechanisms are not required. Rather temporal processing may rely on state-dependent changes in the network dynamics.”

Our suggestions also fit those of Bernstein [57]: “There exist in the higher levels of the central nervous system projections of space, and not projections of joints and muscles.” The present study should be understood as presenting a new repertoire of organizing principles that operate at higher levels of the motor system and may be considered as a possible source for the definition of kinematic primitives.

### Comparison with other studies and possible extensions of the present study

Several other recent studies have also reported strong departures from the two-thirds power law [58],[59]; for locomotion, see also [16],[17]. These studies either employed different experimental paradigms from those used here or offered alternative explanations for the observed violations of the power law.

Schaal and Sternad [58] studied the movement patterns of a human arm (with seven degrees of freedom) during the generation of 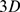 elliptical trajectories. The magnitude of the deviations from the power law depended on the perimeters of the trajectories and on their orientations with respect to the subject's body. To account for these observations, Schaal and Sternad suggested that subjects realize the required elliptical patterns by employing smooth oscillatory pattern generators at the joint level and that the values of the exponent in the power law depends on the geometrical transformations from joint to hand coordinates.

We suggest that the geometric combinations we show are also affected by the geometrical transformations from joint to hand trajectories. Hence, our model, though considering movement duration only from the point of view of hand trajectories, must be further developed to consider Schaal and Sternad's approach and results. We believe that the motor system has evolved to make simplifications in motion planning compatible with the biomechanical characteristics of the musculoskeletal system.

Examining the generation of different patterns of complex figural forms in various tasks and conditions (tracking, drawing from memory, tracing) Flanders et al. [59] also showed significant differences in the values of the exponent of the power law depending on the size and orientation of the trajectory. In particular the authors suggested that strategic or cognitive factors affect the relation between hand velocity and curvature.

This points to many possible extensions of our study. In fact, even if a combination of geometries accounts for the link between geometry and movement duration, we suspect that the rules dictating the mixture of geometrical timing parameters chosen by the brain may depend on external or cognitive factors. It will be particularly interesting to examine in what ways cognitive factors and learning [60] contribute to the effects that global shapes of trajectories have on their local kinematics.

Our present study is limited to 2D motion. Future work should deal with 3D Motion, as well as with movements performed in different orientations, as in [58],[59], and at different depths with respect to the body. This will certainly require considering additional geometries and may reveal certain failures of invariance due to the influence of biomechanical factors.

One limitation of the present study is that we only tested periodic arm movements and locomotion trajectories. However, as suggested above, our principles may also be applied to discrete movements that start and end at rest, or to trajectories containing reversals of movement direction. In such cases all the different geometries are expected to be combined and one may need models that no longer assume constant canonical velocities. Thus new kinds of segments are expected to emerge which depend on the particular velocities combined and on the values of the different geometrical curvatures associated with these velocities.

The principal limitation of the present study is that the tests of the theory have not dealt with the question of which geometrical paths the trajectories should follow. We have only dealt with the question of which velocity is chosen along a prescribed path, as a function of the geometrical form being followed. It is probably not difficult to propose which special paths should be selected, depending on the degree of geometric invariance and symmetry they offer. For instance, as suggested in [51],[52], parabolic arcs are selected in affine geometry, because they remain invariant under several families of transformation. Thus their group of symmetry is rather large.

### Compatibility with optimization theories

Some optimization principles predict the complete actual trajectories. Using only via points and end-points, the minimum jerk principle successfully accounted for both the trajectory path and velocity of curved and drawing movements [4],[61]. Similarly, when the path is fully prescribed, the constrained minimum jerk model successfully predicts the velocities along such paths [44],[50]. The minimum variance principle [62], the optimal feedback control model [63], and the minimum time principle in the presence of signal-dependent noise [64] do predict movement duration, but so far only for point-to-point movements.

The minimum variance principle is grounded on Fitts [65] and Schmidt's laws [66] based on the dependence of average movement duration 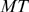 on movement amplitude 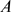 and error tolerance 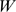, achieved through either a logarithmic function of the ratio 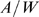 (Fitt's law) or a linear relationship (Schmidt's law) of this ratio. This ratio is invariant under affine transformations, since only ratios between lengths of parallel segments and not absolute values of length appear in these laws. Fitt's and Schmidt's law are therefore *a priori* compatible with affine geometry. In many cases, the predictions of the minimum jerk or minimum variance principles are almost compatible with geometrical invariance.

The use of invariance or mixtures of invariance as proposed here is only a constraint. To realize the actual movements, subjects must apply tools other than, but compatible with, invariance. For example, the geometrical invariance principles can be used together with optimization principles to solve redundancy problems at the task level. Even more importantly, the anticipation of singular points before and during movement generation can help particularly in determining where the motion should be segmented or the precise combination of the canonical geometrical parameterizations to be used. More generally, geometry may indicate in what parametric space or coordinate frames motor commands for movement generation should be planned. Our suggestion does not contradict the need for online optimization of ongoing movements. When needing to anticipate or to respond optimally to trajectory perturbations, optimal feedback, control theory can complement our formulation of invariance principles [63]. This combination of the planning of geometrically invariant trajectories with the anticipation of both geometrical singularities and expected perturbations could allow control of the optimal selection of the relevant parameters.

We emphasize that our model relates naturally to the neural encoding of movement because it suggests the possibility that different neural populations represent movement kinematics in terms of the different geometries or combined geometrical representations:

### The neural basis for several geometries

Evidence has accumulated for the use of different “reference frames” in movement planning ([67]–[71]). For instance, the parietal cortex codes movement in head- or gaze-centered coordinate frames [72],[73], the putamen in a body reference frame [74] and the hippocampus [75],[76] in an environmental reference frame, etc. Furthermore, there is ample evidence that different or even “mixed” coordinate frames are used within the posterior parietal cortex which may be well addressed by the concept of the mixture of geometries suggested here. Target and hand locations during arm movements are represented in terms of eye-centered coordinates, while the motor error between target and hand positions are represented with respect to a hand-based coordinate frame (for review see [77]). In relation to the theory presented here, it may be suggested that the target and the initial hand position are coded in terms of an absolute eye-centered or visually based Euclidian coordinate frames while an evolving coordinate frame, using a motor error between the instantaneous current and the immediate next hand positions, is better characterized as an affine moving frame.

The notion of moving frames (as in section Mathematical preliminaries), particularly the affine geometrical representation, may throw new light on the currently available neurophysiological observations and on the roles of different cell populations in representing movement. Schwartz and colleagues [78],[79] have reported observations consistent with the notion that arm trajectories are well encoded by motor cortical activity in monkeys. A key finding was that the endpoint velocities (including the speed and movement direction) are well represented by single cells and by neuronal populations. This is an instantaneous, relative representation and the magnitude of the population vector was shown to obey the 2/3 power law, while the instantaneous movement direction matched the direction of the population vector [78],[79].

In a recent study Polyakov et al. [52] analyzed the kinematic properties of monkey scribbling movements and the related neural activities of motor cortical units. The scribbling movements were found to be composed of parabolic segments. Using the partial cross-correlation method developed by Stark et al. [80], Polyakov et al. [52] showed that equi-affine velocity was represented more strongly than the Euclidian speed in the activity of several recorded units and the segmentation of the neural activities predicted parabolic segments. Therefore parabolic segments constitute geometrically defined motion primitives which subserve the construction of scribbling movements. This study has also provided the first direct evidence that equi-affine geometry may be used in the neural coding of arm movements.

Consistent with the theory presented here we speculate that there must be many dynamically interconnected neuronal populations, either within one area or more probably within different areas, which use different geometrical representations. These assemblies would be selective for parameters intrinsic to a particular geometry. Some supporting evidence has been obtained in a recent fMRI study [81]. A large number of brain areas responded more strongly to a dot moving along elliptical trajectories with velocities consistent with the 2/3 power law; activity was seen particularly in motor areas (M1, PMd, cerebellum and the basal ganglia) as well as in frontal, cingulate and parietal regions. Brain regions responding more strongly to a dot moving in elliptical trajectories with constant Euclidian were found in the occipital visual areas, the fusiform gyrus and the right parahippocampal gyrus [81]. Neural assemblies within these areas may therefore generate different possible combinations of geometries which may influence movement timing.

### The development of multiple geometries in ontogeny

Analyzing how children draw simple ellipses Viviani and Schneider [43] have shown that both the 2/3 law and the isochrony principle are qualitatively present at 5 years of age and evolve further until age 12. Variability, and geometrical and kinematic distortions of the drawn trajectories diminish greatly between ages 5 and 7 and continue to diminish thereafter. The exponent 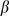 of the radius of curvature in the formula 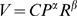, increased from about 0.25 at age 5 to 0.33 at age 12. The exponent 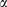 for the perimeter decreased from 0.4 at age 5 to 0.2 at age 12. These findings suggest that Euclidian geometry develops first, followed by equi-affine or affine geometries.

Piaget and Inhelder [82] suggested that the chronological sequence of development of geometric intuition in children is: 1) topology for the most elementary stages of perception, for which only continuity within the spatial field is important; 2) projective geometry, subserving the coordination between prehension and vision through operations that depend on and integrate different points of view; 3) Euclidian geometry utilizing proportions and distances, for perception and storage in memory of places and distances, e.g., in navigation. Piaget and Inhelder suggested that progress is made through the use of concrete operations associated with these different geometries. In their view, affine geometry would be used during the intermediate phases between those associated with projective and Euclidian geometries, e.g., at the beginning of coordination between gaze direction and the direction of body motion during active exploration.

Our data on voluntary movements suggest a different order of development of the different geometries. Implicit motions, unlike explicit or iconic descriptions, seem to be acquired initially using more Euclidian reference frames. This is suggested by the exponent 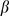 initially being closer to zero in young subjects. Thereafter these exponents move closer to 1/3 [43],[83],[84] possibly reflecting the use of equi-affine or affine representations.

### Final remarks

Every action is a specific solution to a problem. What is *a priori* undetermined by this solution before it is selected is partly encoded by a particular set of symmetries of space and time, a set permitting possible actions at a given level of computation. Any given decision confines the original symmetry group to a specific subgroup, and an action is ultimately chosen when the symmetry is further reduced to the identity. Similarly to perception, geometrical invariance gives motor actions a structure. The most familiar instance of a particular invariance is the global isochrony principle that we interpret as being a manifestation of the use of full affine geometry. Another instance is the 2/3 power law for parabolic segments. However, to be compatible with the strong Euclidian constraint of the physical world and with the restrictions on the computational capacity of the system, full affine invariance is only achieved through the mixing of invariant canonical durations specified by several geometries, such as the equi-affine and Euclidian geometries.

In full affine geometry, time is a pure number (e.g., going around any ellipse takes 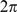). In equi-affine geometry time corresponds to area (the period of an ellipse is proportional to the cubic root of its area), while in Euclidian geometry time corresponds to length. We propose that movement time is continuously selected by the brain based on the combination of these geometrical measures along curves. Still, each individual trajectory is different from all others, since it is associated with a different combination of geometries. Sensation, intention, and cognition can generate particular combinations.

The principle of invariance is also compatible with different optimization principles such as the minimum-jerk [50],[61] or the minimum variance principles [62], and with optimal feedback control [63],[85]. It can even offer a framework within which such principles can be formulated. Invariance, information, feedback and optimality must work together in the selection and adaptation of any movement through evolution and development, but we suggest that by constructing the appropriate spaces at each instant of time along the trajectory, geometric invariance is the main component for determining movement timing.

## Methods

In experimental test no. 1 three young adult men were instructed to draw 10 types of ellipses at 3 different speeds, slow, natural and fast. The ellipses, prescribed in advance, had 3 different eccentricities, 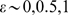, and 3 different sizes, small, medium and large, plus one ellipse which was “as large as possible” called *huge*. Within each session, each ellipse was drawn 10 times and statistical analysis was performed based on 8 repetitions, ignoring the first and last drawings. (For further details, see section B.1 in Text S1.)

In experimental tests no's 2 and 3, we analyzed a series of drawings and locomotion trajectories of cloverleaves, limaçons and lemniscates, taken from the studies of Viviani and Flash [4] for drawing movements and from Hicheur et al. [17] for locomotion.

For drawing, the trajectories were those of the stylus position along the tablet. For locomotion the trajectories measured were those of the orthogonal projection 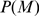 on the ground of a point 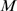 corresponding to the mid-point between the subject's shoulders. To verify the stability of the geometrical models, the trajectory of a point marked as the *R*-point was also considered. The *R*-point is the intersection on the floor of the line connecting the *M*-point with the mid-point between the sensors positioned on the subject's chest and back (see Figure S3. For the results of the *R*-point see section D.4 in Text S1, and related figures. In particular, Figure S9 shows the results we obtained for the locomotion data using the R-Point. Figure S10 shows for both drawing and locomotion (M-Point and R-Point), the mean values of the 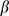 functions for the different figural forms and for different subjects. Figure S11 shows the mean values of the 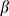 functions, separately for the small and big loops of the limaçons and lemniscates, for drawing and locomotion (both for the M-Point and R-Point).

In both analyses we started with a collection of point coordinates 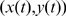, registered at time intervals of 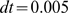 for drawing and of 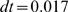 for locomotion (200 Hz and 60 Hz respectively). This gives N points. From the total sample set, a smooth geometric trajectory was constructed, without considering the actual timing. This was achieved by separately approximating the position data for the 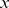 and 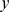 coordinates using two Fourier series 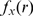 and 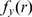 with 8 harmonics (each with 17 independent real coefficients), 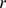 being the parametrization used for the Fourier series. From the smooth path 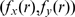 we derived the various curvatures (Euclidian, equi-affine, affine) and deduced the 3 monotonic velocities (see equation set 7). In addition we computed the velocity predicted by the constrained minimum-jerk model [44]. For both types of calculation we selected the corresponding time parametrization which is independent of the actual experimental one. We now needed to find the correspondence between the experimental time series of position coordinates 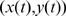 and the position on the smooth path obtained from the Fourier approximation. Hence, we calculated 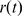 by projecting each point of the experimental trajectory on the Fourier approximated path 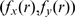. We then used the new parametrization 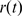 to derive velocities for the smoothed experimental data (for more details see section D.2 in Text S1).

These calculations were conducted on the entire N samples obtained from the drawing and locomotion data. For locomotion we called this data set the complete sampled data set (CSDS). For locomotion, we also extracted the data corresponding to positions where the point 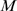 attained a local minimum altitude above the ground, giving 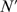 points. We called this a stepwise sampled data set (SSDS). To compare the different velocity profiles we needed to compare velocities occurring at the same points along the same curve. To do this, we found a set of 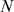 points 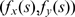 located at an equal Euclidian distance 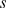 from each other. For all models, the velocities at these points were calculated using a standard cubic spline interpolation. Note that the number of independent raw data points used for calculating each value of experimental velocity profile was 5 or less, so the number of “statistically independent degrees of freedom” used below was estimated as 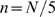 for drawing and for CSDS for locomotion. For SSDS for locomotion all 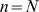 were used.

The velocity 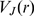 derived according to the constrained minimum-jerk model depends only on one parameter corresponding to the total time. The other theoretical velocities 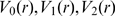, termed affine, equi-affine and Euclidian “uniform velocities” were computed based only on the path coordinates.

To choose the mixture of these uniform velocities which results in the predicted combined velocity, we looked for segments of the experimental velocity during which we could set at least one of the weight function *β*'s as a constant. We then used a cubic spline interpolation for computing the remaining functions' 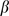 between these segments. (For locomotion we used the experimental step velocity, based on the SSDS samples). Seven different algorithms were used for this calculation. The geometrical combination chosen was that giving the best theoretical velocity profile compared with the experimental velocity and which involved the lowest number of parameters.

In the first algorithm we used a linear regression in logarithms of velocity and found segments between points where we could determine 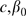 and 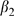, such that the experimental velocities could be well approximated by:
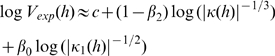
(15)representing straight lines (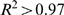) with a length of at least 30 points. Here 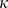 and 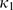 mark the absolute values of the Euclidian and equi-affine curvatures, respectively. This equation is based on equations (7), (8) and the fact that 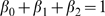.

For the second algorithm we *a priori* imposed 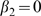. The new equation we obtained from equation (15) is:
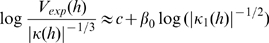
(16)As in the first algorithm we found segments during which the equation represents a straight line (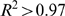). We then used spline interpolation to set the values of the 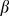 weight function between those segments.

In the third and fourth algorithms we considered the combination of affine and Euclidian geometries and the equi-affine and Euclidian velocities respectively. The equations used were, respectively,
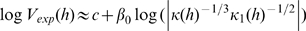
(17)and
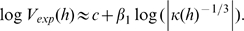
(18)As in the second case, we constructed the theoretical velocity from the segments of straight lines.

The velocities constructed in cases two, three and four were marked as 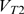,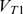 and 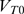, respectively. we explicitly used these velocities for the last three algorithms. By dropping the assumption that 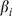 equals zero we obtained 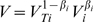. Hence, for 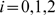 we looked for segments of straight lines in the new equation
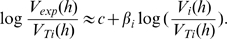
(19)


All these algorithms were based on the following arguments. First, we expected to find segments during which a constant combination of geometries appears the primary source for movement segmentation. Second, we had no reason to believe that constant combinations of all three geometries would appear at the same time, so we looked for two-by-two constant combinations. However, to reduce the number of parameters, based on the data we limited the algorithm to the same pair of geometries all along the trajectory.

This procedure required verification that these segments (primary and secondary) were statistically non-trivial. We therefore used a Fisher's test (see below), as explained in section Experimental tests. Our modeling approach also required verification that the success of the model was not only a consequence of our using a large number of fitted parameters. For this purpose we used the Akaike criterion (AIC) as explained in section Experimental tests.

The F-test: The data used were those of the logarithms of the velocities. For each curve and for each of the seven computational scenarios, let 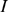 denote the union of the 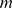 special intervals of total length 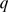 and 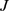 the complementary part of the curve, of total length 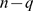. Recall that on 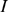 the model logarithmic velocity 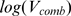 was directly extracted using the values of 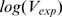 and a linear combination of two of the calculated 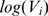. The number 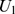 is the residual sum of squares, 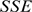, on 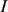:
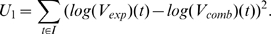
(20)The quantity 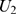 is the total sum of squares, 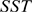, for the entire curve:
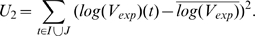
(21)where 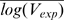 is the mean value of the experimental velocity. As usual we can write 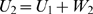 where 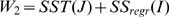, and we hypothesize that the random variables within 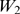 are independent of the variables within 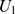. With this hypothesis the scaled ratio 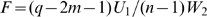 follows a Fisher law 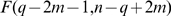 (see [86]). Note that the number of degrees of freedom (df) for 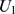 is 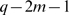 because 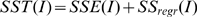. We fixed two independent parameters for each connected special segment The df is 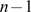 as usual for 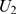, so we obtain 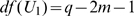 and 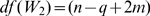. The second formula comes from the following decomposition
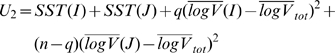
We then repeated the above computation by replacing the mean value of the experimental velocity by its approximation using a trigonometric approximation up to the fourth order. When using the trigonometric polynomials of degree 4, we still have 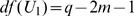, 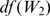 changes from 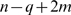 to 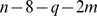, because a trigonometric polynomial of degree 4 depends on 9 real numbers, the constant being the mean of the function.

For drawing, 61 of 78 trials (78%) showed a *P*-value of significance equal to 0.005 in the *F*-test. For locomotion 65 of 91 trials (71%) satisfied the test. All the results are shown in Table 4. This gives the probability that the variance with respect to the mean or, respectively, with respect to the trigonometric approximation of degree four, is sufficient to explain the presence of the detected segments. We verify that this probability is very small according to the standard linear F-test. The results confirm the non-triviality of the existence of segments.

The Akaike test [45],[87]: if 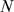 is the number of data samples, 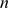 is the number of independent data samples, and 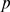 is the number of parameters adapted from the data and used by the tested model plus one, we used the following expression:
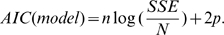
(22)


## Supporting Information

Figure S1 Log γ vs. Log A for each subject. All the repetitions for each ellipse size and speed condition are grouped into a single dot, the y-axis the log γ values. The diamond shape plotted around the mean value ±1 displays the standard deviation for both axes. The results for each subject are shown in different figures. In all figures, blue represents slow drawing speed; green, the natural speed; red, fast drawing speed. The dashed lines gives the regression lines separately for each speed. The parameters of the lines are given in table 1 in the main paper.
(1.56 MB EPS)

Figure S2 The geometrical shapes used in the second and third tests. The analytical shapes and the parametric equations of the asymmetrical lemniscate, the oblate limaçon and the cloverleaf used in the drawing and locomotion experiments analyzed by the second and third tests.
(0.96 MB EPS)

Figure S3 The reference points used in the locomotion experiments. The M and R reference points marked on the subject's body for the locomotion experiments.
(1.74 MB EPS)

Figure S4 Examples of the experimental data and the geometrically based predicted velocities for the drawing movements. Every row is an example of the second repetition of a trial. First row, typical example of drawing a cloverleaf; second row, drawing an oblate limaçon; third row, drawing an asymmetric lemniscate. Panels (A),(D) and (G) show the paths drawn by the subject, colors represent the Euclidian curvatures along the curves: blue, low curvature; red, high curvature. Panels (B), (E) and (H) show the velocity profiles of the movements: red, experimental velocity used by the subject; green, the velocity profile under Euclidian parameterization; black, the velocity profile under equi-affine parameterization, blue, the velocity profile under affine parameterization. Panels (C), (F) and (I) show the curvatures of the curve; green, Euclidian curvature; black the equi-affine curvature; blue, the affine curvature.
(1.52 MB EPS)

Figure S5 Examples of the experimental data and the geometrically based predicted velocities for the locomotion experiment using the M-point. Every row gives an example of the second repetition of a trial; first row, a cloverleaf; second row, an oblate limaçon; third row, an asymmetric lemniscate. Panels (A),(D) and (G) show the gait paths generated by the subject. The colors used in plotting the paths represent the Euclidian curvature along the path; blue, low curvature values; red; high curvature values. Panels (B), (E) and (H) show the velocity profiles of the movements. Red, the velocity used by the subject; green, the velocity profile under Euclidian parameterization; black, the velocity profile under equi-affine parameterization; blue, the velocity profile under affine parameterization. Panels (C), (F) and (I) show the curvatures of the curve; green, Euclidian curvature; black, the equi-affine curvature; blue, the affine curvature.
(1.86 MB EPS)

Figure S6 Examples of the β functions and the combined velocities for the drawing experiment. Every row gives an example for the second repetition of a trial. The first row, cloverleaf; second row, an oblate limaçon; third row, an asymmetric lemniscate. Panels (A),(D) and (G) are the paths drawn by the subject. The colors marked on the paths represent the β functions: blue, a part that is more Euclidian; green, a part that is more equi-affine; red, a part that is more affine. The full range of colors and their relation to the values of β_0_, β_1_ and β_2_ can be seen in Figure S8. Panels (B), (E) and (H) display the velocity profiles for these movements: red, the experimental velocity used by the subject; blue, the theoretical combined velocity profile. Panels (C), (F) and (I) display the values of the β functions. Red area, the values of the β_0_ function; green area, the values of the β_1_ function; blue area, the values of the β_2_ function.
(1.52 MB EPS)

Figure S7 Examples of the β functions and the combination velocity of the locomotion. Every row gives an example of the second repetition of a trial. The first row, a cloverleaf; second row, an oblate limaçon; third row, an asymmetric lemniscate. Panels (A),(D) and (G) show the path generated by the subject. The colors on the paths represent the β functions: blue, a part that is more Euclidian; green, a part that is more equi-affine; red, a part that is more affine. The full range of colors and their relation to the values of β_0_, β_1_ and β_2_ can be seen in Figure S8. Panels (B), (E) and (H) show the velocity profiles of the curve: red, the experimental velocity used by the subject; blue, the theoretical combination velocity profile. Panels (C), (F) and (I) display the values of the β functions: red area, the values of the β_0_ function; green area, the values of the β_1_ function; blue area, the values of the β_2_ function.
(4.10 MB EPS)

Figure S8 Color map for the values of the βs. Every point in the triangle represents a specific relation between β_0_, β_1_ and β_2_ values shown in the color corresponding to this combination.
(0.61 MB EPS)

Figure S9 Results in the locomotion experiments using the R-point. Panels (A) and (B) represent the R^2^ and AIC scores for the CSDS (all data) of the locomotion R-point, respectively, for the combined velocity (red bars), minimum-jerk velocity (green bars), constant equi-affine velocity (yellow bars) and constant affine velocity (cyan bars). The probability of the combined velocity being a better model than the minimum-jerk model for the different figural forms is shown in Panel (C). Panels (D) and (E) represent the values of the functions β_0_, β_1_ and β_2_ averaged over all trials. The results are presented for the R-point at the level of the figural form. The cloverleaf form is marked by *C*. The marking *L*_1_,*L*_2_,*L*_3_ and *A*_1_,*A*_2_,*A*_3_ represent the limaçon and the lemniscate templates, respectively, according to the ascending ratio of the large to the small loops.
(1.17 MB EPS)

Figure S10 The mean values of the β functions for the different figural forms and different subjects. The mean values of the functions β_0_, β_1_ and β_2_ averaged over each trial, summarized over the subjects and the templates of the different figural forms. Every β is displayed in a separate figure. Every color represents a different subject. Every group of bars represents a different figural form. The cloverleaves are marked by *C*_1_,*C*_2_,*C*_3_ in the order of ascending speed for drawing and by *C* for locomotion. The marking *L*_1_,*L*_2_,*L*_3_ and *A*_1_,*A*_2_,*A*_3_ represent the limaçon and the lemniscate templates, respectively, according to the ascending ratio of the large to the small loops.
(1.18 MB EPS)

Figure S11 The mean values of the β functions averaged over loops and figural forms. The mean values of the functions β_0_, β_1_ and β_2_ over loops within a trial, summarized over the templates of the figural forms. Every β is displayed in a separate figure. Blue bars, the small loops; red bars, the large loops. Every group of bars represents a different figural form, for notations see Figure S10 and section D.1 in Text S1.
(0.98 MB EPS)

Text S1 Supporting Information. Mathematical background, data processing and additional results.
(0.25 MB PDF)
