# Supplementary material for: Movement Timing and Invariance Arise from Several Geometries
Source: PLoS Comput Biol. 2009 Jul 10;5(7):e1000426. doi: 10.1371/journal.pcbi.1000426 (PMC2702097; doi:10.1371/journal.pcbi.1000426)
Supplement: Text S1 — Supporting Information. Mathematical background, data processing and additional results. (0.25 MB PDF) [file pcbi.1000426.s012.pdf]

Supporting Information (Text S1)

Mathematical background, data processing and additional results

Daniel Bennequin, Ronit Fuchs, Alain Berthoz, Tamar Flash

Contents

A Mathematical background **2**

A.1 Curves, jets, groups and moving frames 2

A.2 Three examples of Cartan frames 7

A.3 Degeneracies 21

B First test: elliptical trajectories **23**

B.1 Data recording and processing 23

B.2 Theoretical and experimental testing of the law of area 23

B.3 Limits of isochrony 25

C Global scaling **27**

D Second test: complex forms: velocity prediction **27**

D.1 Data recording and approximation 27

D.2 Curvature and velocity computations 28

D.3 Geometrically combined velocity 29

D.4 Expanded results 32

E Third test: complex forms: timing **36**

E.1 Proportion and segmentation 36

E.2 Data recording and processing 40

A Mathematical background

A.1 Curves, jets, groups and moving frames

Suppose that certain transformations act on certain objects, then a theory of invariance is a theory controlling how geometrical characteristics of objects do or do not change under these transformations. For example, an invariant quantity is a function of the considered objects which stays unchanged after all transformations considered. Invariance is better understood through the mathematical concept of group theory. Recall that a set of transformations is a *group* when these transformations can be composed and inverted. All the groups we discuss below are formed by transformations of space or of the plane, and the objects they transform will consist of trajectories or of elements of trajectories. The largest group we will consider is called the *affine group*, or full affine group; it contains translations, rotations, dilatations, stretching, and shearing.

Mathematicians have applied two approaches to define the notion of affine geometry: one is intrinsic and axiomatic (applied by Euclid, followed by Hilbert and then Artin). This approach begins with abstract notions of points, lines and parallelism. The other approach is computational and concrete and uses coordinates and vectors. A 2-dimensional vector space $\mathbb{E}$, which can be easily identified as the set $\mathbb{R}^2$ of pairs of real numbers, operates on the affine plane $\mathbb{A}$, such that any pair of points (A, B) in $\mathbb{A}$ determines a unique vector $\vec{v} = \overrightarrow{AB}$ which sends A to B, and this is expressed by $B = A + \vec{v}$. By choosing a point O this identifies the plane $\mathbb{A}$ with the vector space $\mathbb{E}$ (by sending any point M to the vector $\overrightarrow{OM}$), and then by choosing coordinates on $\mathbb{E}$, we identify $\mathbb{A}$ with the numerical space $\mathbb{R}^2$.

We mostly disregard Euclidian distance and retain only invariant affine concepts. The main hypothesis of the current paper is that affine invariance is used for motion planning and execution, but we also explore the hypothesis that this invariance is incomplete and must be corrected by measures of distance and area, involving, respectively, Euclidian and equi-affine invariance.

In any geometry, the end-effector position is represented by translation. In Euclidian geometry the tangent direction of any given motion can be represented by a rotation. In affine geometry, however, the tangent direction can also be represented by stretching or by some other transformation which depends

on the rigidity of the moving frame. What requires no special choice is the set of all frames whereby the first vector has a fixed direction. At a higher order of description, several measures can be used to describe the departure of the trajectory from motion in a straight line: the most usual measure is the Euclidian curvature but we will see that this is not an invariant measure from an affine point of view. Consequently, in affine geometry we have a totally different notion of turning, represented by special sets of frames. There is evidence to believe that in addition to the usual measure of turning, this latter additional measure of turning is used to control movement duration. This evidence is provided by the known tendency towards isochrony, i.e. the tendency of separate segments to have nearly equal movement durations when corresponding to each other through dilatation. We will show how, when moving along any curve, affine invariance offers convenient concepts for controlling the successive higher orders of time derivatives of position, and how it is possible to construct stable mixtures of invariance through the use of several geometries.

In what follows, a *frame* is simply a coordinate frame in the plane formed by a point and by two vectors attached to it. Suppose a transformation group G of a given space or of the plane is given, the mathematical theory of moving frames consists of the construction of special sets of frames, such that the action of G on these sets of frames faithfully reflects the action on differential elements of curves. We will show how this theory provides a representation of the differential elements of a trajectory by using invariant functions, invariant parameterizations of the trajectory, and subgroups of G that measure indetermination on the frames. (In the next paragraph we define precisely the notion of differential elements of a geometric curve at any finite order of contact.)

For simplicity we limit our investigation to a fixed plane $\mathbb{A}$. We consider trajectories $M(t)$ of a point M when moving in this plane as a function of time t. But we wish to describe an infinitesimal piece of a curve independently of any parametrization. For computational purposes it is frequently useful to choose coordinates (x, y) on $\mathbb{A}$. With the help of these coordinates it is easy to define the differential elements at order n of a curve, they are called the *n-jets of curves* or the *contact elements* of order n. We denote the corresponding set as $V_{(n)}$. Concretely, an element of $V_{(0)}$ is a point $M_0 = (x_0, y_0)$, an element of $V_{(1)}$ is a straight line Δ_0 with the point M_0 located on it. The line Δ_0 is the tangent to the curve through M_0, which can be described by the equation $y - y_0 = a_0(x - x_0)$, so a point in $V_{(1)}$ is described by three

numbers x_0, y_0, a_0 and so on. Similarly, a point in $V_{(n)}$ is described by $n+2$ real numbers. When a curve Γ is expressed through the equation $y = f(x)$ and $M_0 = (x_0, y_0)$ is a point on Γ, the corresponding contact element in $V_{(n)}$ is represented by the collection of numbers $(x_0, y_0, f'(x_0), f''(x_0), \ldots, f^{(n)}(x_0))$, where f' is the derivative of f, f'' is its second derivative, and so on, $f^{(n)}$ being the $n-th$ derivative of f. A change of coordinates induces a change in the numbers describing the jets, but it respects the order n. For instance, intrinsically $V_{(1)}$ is the tangent line, without a unit of length being defined along it. $V_{(2)}$ is more difficult to visualize, it does not correspond to a unit length along this tangent. In fact, two regular curves Γ_1,Γ_2 give the same element in $V_{(2)}$ at a common point P when they have the same tangent T and if, for any affine coordinates chosen in the plane with the x-axis pointing along T and the y-axis being transverse to T, the difference of the coordinates $|y_1 - y_2|$ between the two curves, decreases at least as the power $|x|^3$ when x goes to 0.

The main point of the Moving Frame Method (developed by Darboux and Cartan) is to associate infinitesimal properties of trajectories with algebraic properties of a certain group of symmetries acting on the plane. More precisely, we first select a set G of transformations of $\mathbb{A}$ onto itself, this set being closed under both inversion of transformations and composition of transformations. The group must also be sufficiently large to insure that every point and direction in the plane can be sent to any other point and direction. This defines what is called a geometry by Klein and Poincare. The operation of G on the points in the plane produces the operation of G on the set $V_{(n)}$ of jets of curves of order n and, following Cartan, we try to understand this operation on n-jets by a model inside the group G itself, a model given by translation of a certain subgroup. As we will see, this automatically gives a canonical means for transporting a coordinate frame along the curve, producing a consistent way of representing any point in the plane depending on the movement being generated.

We construct a sequence of groups $G_{(0)}, G_{(1)}, ..., G_{(n)}, ...$, where each group is included within the previous one (i.e., $G_{(n+1)} \subset G_{(n)}$). For any curve Γ in the plane, and for any point P on Γ, a subset $g_n G_{(n)}$ in G and a sequence of numbers $(u_0, ..., u_{k_n})$ will be associated one-to-one with a jet of order n. This is done so that the natural restriction of jets from order $n+1$ to the jets of order n corresponds to the restriction of the invariant functions and to the inclusion of the subgroup $G_{(n+1)}$ within $G_{(n)}$.

A fixed *affine frame* F_0 for the affine plane $\mathbb{A}$ is chosen for our analysis consisting of one point O as the origin and two vectors e_0, f_0. (However, note that at the end we obtain results which are independent of the choice of F_0). The element g_n generates a frame g_nF_0, denoted by F_n when $n \geq 1$ and by $F_{(0)}$ when $n = 0$, which is called a *frame of order n*. Observe that this element g_n is only determined modulo $G_{(n)}$, meaning that one is permitted to replace g_n by g_nh for any element h of $G_{(n)}$ and then to replace g_nF_0 by $g_nhF_0 = (g_nhg_n^{-1})(g_nF_0)$. Hence, and this is an important feature in the moving frame theory, frames of order n form a set which is in correspondence with equivalence classes of elements in G modulo the subgroup $G_{(n)}$. We call $G_{(n)}$ the *ambiguity subgroup* at order n, because it measures the residual ambiguity on the frame at the order n of the curve elements.

Here we make a conceptual remark: The subgroup $H_{(n)}=g_nG_{(n)}g_n^{-1}$ of G depends only on the considered jet but is independent of the choice of g_n. Thus, this group properly counts the residual indeterminacy at the order n on the frame. The difference between $H_{(n)}$ and $G_{(n)}$ is: $G_{(n)}$ is universal and measures the geometrical indeterminacy in the ambient space (or, as here, in the plane) in the considered geometry given any jet of order n of curves, but $H_{(n)}$ varies with the given jet of order n. In the analysis all the matrices presented are belonging to the subgroups $G_{(n)}$.

The numbers $u_0, ..., u_{k_n}$ are called the *invariants* of Γ up to order n. Their meaning is as follows: two curves have the same invariants up to order n if and only if there is a transformation in G which makes one of the curves equivalent to the other curve up to the order n.

Note that in our main application only invariant quantities and invariant parameterizations appear, while the constructed canonical frames are not explicit. However, we think that these canonical frames are the main concept underlying the invariant parametrization, because they naturally generate such parametrization and because they permit description of the entire plane starting only with a given curve.

When n becomes larger, we examine a given curve segment with respect to higher and higher geometric orders, a point of order zero, a tangent of order one and so on. The subsets $g_nG_{(n)}$ are included one within the other in a decreasing manner. Finally, one obtains a set of one and only one element in G, which gives the Canonical Moving Frame belonging to the geometry G. The miracle that justifies the

application of this theory to the problem of selecting the timing of motion along a given trajectory is that obtaining the unique canonical frame is sufficient for selecting a parametrization for the curve and this parametrization can then be considered as a candidate for prescribing movement kinematics along the curve. That is, duration results from the geometry. However, different geometries can be considered and, consequently, different timing parameterizations can be applied. Hence, we must understand how these different parameterizations can be chosen and combined.

Depending on the curves considered, the invariant quantities derived after the frames are computed are the curvature in G and its successive derivatives. For example we encounter the full affine and the equi-affine curvatures.

Assuming that G is a continuous proper sub-group of the group of differentiable isomorphisms of the plane, the possible choices for G were classified by Lie and Klein [1–3] These groups all depend on the choice of a particular affine structure in the plane.

Let us choose affine coordinates on $\mathbb{A}$. The full affine group G is the group of all affinities:

$$\begin{aligned} X &= ax + by + u \\ Y &= cx + dy + v \end{aligned} \tag{S1}$$

where a, b, c, d, u, v are specified real numbers such that $ad - bc \neq 0$, and where (x, y) are the coordinates of some general point in the plane and (X, Y) the coordinates of its image obtained by the affine transformation.

Within the affine group we obtain the equi-affine subgroup G^1, expressed by exactly the same formulae but for which $ad - bc = \pm 1$ is additionally imposed. To simplify our presentation, below we consider only the sub-group SG defined by $ad - bc = 1$, which is called the *special affine* group.

Inside G^1 (resp. SG) there are many subgroups defining more rigid geometries, and all these subgroups are associated with an Euclidian structure, that is, with a metric which is a distance function

compatible with the affine structure. The multiplicity of choices for these metrics is resolved by choosing particular coordinates, because in $\mathbb{R}^2$ we naturally first have the ordinary metric (given by the square root of the sum of difference of coordinates). However, we must understand that another choice of coordinates would have produced a completely different metric. Only rotations and translations, possibly composed with orthogonal symmetries, have no effect on the distance metric. In fact, a little thought convinces us that these metrics correspond to the oriented ellipses centered on a given point.

When one metric and the orthogonal coordinates associated with it are chosen, the Euclidian group of affine isometries SGE is described inside SG by the equations

$$
\begin{aligned}
X &= x\cos\theta - y\sin\theta + u \\
Y &= x\sin\theta + y\cos\theta + v
\end{aligned}
\tag{S2}
$$

To obtain the complete Euclidian group GE of this metric, we must add all reflections (i.e. symmetries with respect to straight lines), which are described by the above formulae except for the modification, whereby Y must be changed into $-Y$ in the second equation.

In the following section we give a complete exposition of the moving frame method for the case of Euclidian, equi-affine and affine geometries of the plane. The full affine case has not yet been described in detail in the literature (cf. [4–6]).

A.2 Three examples of Cartan frames

In this section we describe the computational principles of the moving frame method in order to gain understanding of geometric canonical parameters and of canonical frames. We particularly hope to see how canonical parameters emerge from the inspection of higher order contact elements of the curve, order after order. For those who are not geometricians, this study is not so simple, so we first very quickly summarize the main results, without mentioning the method itself.

In Euclidian geometry, the parameter considered is the Euclidian arc-length, and the moving frame

is formed by an origin "the location at which you presently are", the first unit vector pointing towards "the location at which you will immediately be", that is, the tangent vector, and a second vector describing all the points within the plane represented by the values of the coordinates along the axis which is "perpendicular to the tangent vector", that is the normal vector. The only subtlety is with respect to orientation, whether you are moving towards the front or towards the back, respectively, towards the left or the right.

In equi-affine geometry (which can be seen as a geometry with a given unit of area, or as a relative affine geometry where "area preservation" is important), things become more complicated. The canonical parameter generates the 2/3 power law. There is no invariant measure of turning that plays the same role as Euclidian curvature, but there is an invariantly defined normal equi-affine direction. The canonical equi-affine computation establishes the normal direction by a "parabolic approximation". That is, the parabola nearest to any given curve has a "conjugate direction", this direction being that which bisects any secant segment parallel to the tangent direction. The first invariant appearing is the equi-affine curvature k_1, which can be understood as the "size of the area lying between the conic closest to the curve and the oscillating parabola". This area is negative if this conic is an ellipse, positive if it is a hyperbola and 0 if it is a parabola. The equi-affine curvature is an invariant of order 4, where there is no invariant of the order 3. For motion planning this implies that no equi-affine invariant appears at the third order, which is the order of jerk.

In affine geometry, things are much more unusual. No invariant appears even at order 4, because only the *relative* change in the size of the area between the oscillating parabola and the closest conic is important in full affine geometry. The canonical parameter σ generates no power laws, except for moving along very special curves, such as conics where affine geometry gives the same 2/3 power law as when using the equi-affine parameter σ_1. The concrete interpretation of σ is achieved through a very basic scaling law: when using σ as parameter, the transverse distance (along the affine normal) scales as the square of the tangential distance (along the tangent). Note that, apart from this latter property, the direction of the normal affine axis is the same as in equi-affine geometry and is obtained by finding the conjugate of the nearest parabola. The main novelty of using affine parametrization is that, when time is only a function of σ, full isochrony is achieved. Hence, if two pieces of curves are transformed one into the other by an

affine transformation, these curves are traversed with the same total duration. It is this phenomenon which can be called "localization of isochrony". The simplest example is again obtained when examining conics: you "observe a circle" and then you move yourself along it. What you have planned is a constant Euclidian velocity, but when superimposed on what you really see, which is an ellipse, your actual velocity fits the 2/3 power law of motion. Of course, measurement of area is not relevant here and the motion plan is affine invariant.

Now let us begin with our mathematical derivation:

First, for illustration and to give the flavor of the Cartan method, using a very simple case, we apply this method to Euclidian geometry. Let us fix an Euclidian metric on the affine plane and denote by GE the group of isometries (Euclidian transformations). We also fix an Euclidian reference frame F_0 consisting in this case of an origin O and of two unit orthogonal vectors e_0, f_0. The subgroup of isometries fixing the point of origin O is the so-called orthogonal group $O_2(\mathbb{R})$, it is the convenient inertia group $GE_{(0)}$ for classifying curves at the order 0. At this order only the point O is important. Every point M in $\mathbb{A}$ can be obtained from O through a translation, but it can also be obtained through a rotation around O which can be followed by another translation. Thus the ambiguity of the action is described by $O_2(\mathbb{R})$, and for the order zero, nothing else need to be said.

At the order one, the inertia group of order 1, $GE_{(1)}$ has to globally respect the direction of the first vector (axis) of the frame F_0. This inertia group has four elements and it is traditionally called the Klein's group. By choosing an orientation of the plane $\mathbb{A}$, the inertia of degree 0 is reduced to the special-orthogonal transformation $SO_2(\mathbb{R})$, and by additionally choosing an orientation along the examined curve Γ, we reduce the inertia of degree 1 to the identity group Id. Hence, a canonical frame appears at the order 1 and it is the ordinary Frenet-Serret frame. Because the unit vector along the tangent is established, this frame is accompanied by a canonical parameterization, which is well defined up to an additive constant at order one: the Euclidian arc-length s.

The invariant appearing at the order two is the Euclidian curvature κ. The radius of curvature is the inverse of the Euclidian curvature $R = 1/|\kappa|$ in $[0, \infty]$. The canonical infinitesimal equations in this case

are due to Frenet-Serret, and they describe the motion of the canonical Euclidian frame as

$$\begin{aligned} \frac{dM}{ds} &= J_1 \\ \frac{dJ_1}{ds} &= \kappa J_2 \\ \frac{dJ_2}{ds} &= -\kappa J_1 \end{aligned} \tag{S3}$$

In this sense, any smooth curve (with a continuous tangent) in Euclidian geometry determines the plane, once a direction (back or front) and a normal direction (right or left) are chosen.

Now let us apply the same moving frame method to equi-affine geometry (as Explained, for instance, in [4]). For simplicity we restrict ourselves (as Cartan did) to the subgroup SG of G^1 which is defined by the preceding equations (S1) by imposing, in addition, the special condition $ad - bc = +1$. The reference frame F_0 is fixed once and for all as before, and it particularly determines a well defined unit of area in the affine plane by assuming that the parallelogram with sides e_0, f_0 has an area equal to 1.

At the order 0, as in the Euclidian case, the element g_0 has only to transform O into M, and the ambiguity of degree zero is described by the subgroup $SG_{(0)}$ of SG defined by $u = v = 0$ when we use the notation of (S1); it is traditionally named $SL_2(\mathbb{R})$:

$$SG_{(0)} = SL_2(\mathbb{R}) = \left\{ \begin{pmatrix} a & b \\ c & d \end{pmatrix} \middle| ad - bc = +1 \right\}. \tag{S4}$$

In the second step, to obtain a moving frame of order 1, F_1, since we have already fixed the point M, we only have to fix the first vector in the direction of the tangent vector. Note that we do not yet know the size of that vector. To find the subgroup $SG_{(1)}$, let $R = (M, I_1, I_2)$ and $\overline{R} = (M, J_1, J_2)$ be two smooth fields of coordinate frames centered along the curve Γ, both depending on an arbitrary parametrization t. The two frames are transformed one into other by an element of $G_{(0)}$:

$$I_1 = aJ_1 + cJ_2. \tag{S5}$$

$$I_2 = bJ_1 + dJ_2. \tag{S6}$$

As the vectors I_1 and J_1 are maintained in the direction of the tangent vector to the curve, we get that $c = 0$. In addition $ad - bc = 1$, hence $a = d^{-1}$. For convenience let us set $a = \lambda$ and $b = \mu\lambda^{-1}$.

The analog of $GE_{(1)}$ is now the sub-group of SG formed by matrices respecting the first axis of the coordinate frame (which corresponds to the tangent in the moving frame). It is the group of upper triangular matrices of determinant one, denoted by $SG_{(1)}$:

$$SG_{(1)} = \left\{ \begin{pmatrix} \lambda & \mu\lambda^{-1} \\ 0 & \lambda^{-1} \end{pmatrix} \middle| \lambda \neq 0 \right\}. \tag{S7}$$

and there is no invariant of order 1.

To find the set of moving frames F_2 and the subgroup $SG_{(2)}$ of order 2, let us look again at the frame coordinates $R = (M, I_1, I_2)$. Then the movement of the frame R along the curve, described at the first order of approximation, is defined by the following general infinitesimal *moving frame* set of equations:

$$\begin{aligned} dM &= \omega_1 I_1 \\ dI_1 &= \omega_{11} I_1 + \omega_{12} I_2 \\ dI_2 &= \omega_{21} I_1 + \omega_{22} I_2 \end{aligned} \tag{S8}$$

In these formulae, the symbol ω_1 denotes the differential form dt of the given parameterization. The other coefficients are also differential forms. It can be easily shown that the equi-affine restriction of the frames to SG implies $\omega_{11} + \omega_{22} = 0$. [It is because the preceding system (S8) represents the first order in the variation of the frame: the coordinates of the vectors $I_1(t_0 + \varepsilon) = ..., I_2(t_0 + \varepsilon) = ...$ expressed as linear combinations of $I_1(t_0), I_2(t_0)$ become $a(t_0 + \varepsilon) = a(t_0) + \varepsilon\omega_{11} + ...$, and so on. Moreover $a(t_0) = d(t_0) = 1, b(t_0) = c(t_0) = 0$, so when we write that the equality $ad - bc = 1$ holds true for each value of $t = t_0 + \varepsilon$, by equating the coefficient of ε to zero, we obtain $\omega_{11} + \omega_{22} = 0$.]

We now obtain a convenient *measure of turning* in the equi-affine sense by the following definition: $b_1 = \omega_{12}/\omega_1$. Note that the value of b_1 depends on the chosen family of frames R because ω_1 and ω_{12}/ω_1 depend on it. The case of $b_1 = 0$ is exceptional; the tangent is stationary, meaning that the curve Γ

has an inflection at the point studied. Except for the case $b_1 = 0$, we show the existence of special smooth families of frames along the curve, such that b_1 takes the value 1. Let us look again at a path of transformations from $R = (M, I_1, I_2)$ to $\overline{R} = (M, J_1, J_2)$ such that this time the transformations belong to $SG_{(1)}$, we get:

$$\begin{aligned} I_1 &= \lambda J_1 \\ I_2 &= \lambda^{-1}(J_2 + \mu J_1) \end{aligned} \tag{S9}$$

Let us write the moving frame equations for $\overline{R}$:

$$\begin{aligned} dM &= \overline{\omega_1} J_1 \\ dJ_1 &= \overline{\omega_{11}} J_1 + \overline{\omega_{12}} J_2 \\ dJ_2 &= \overline{\omega_{21}} J_1 + \overline{\omega_{22}} J_2 \end{aligned} \tag{S10}$$

We then have

$$dM = \omega_1 I_1 = \overline{\omega_1} J_1,$$

Consequently

$$\overline{\omega}_1 = \lambda \omega_1.$$

Moreover, if we derive the first equation in equation set (S9) and use equation set (S10) we get

$$\begin{aligned} dI_1 &= \lambda dJ_1 + d\lambda J_1 = \\ &= \lambda(\overline{\omega_{11}} J_1 + \overline{\omega_{12}} J_2) + d\lambda J_1. \end{aligned}$$

Now we can replace J_1 and J_2 by using equation set (S9) and get an equation that contains only dI_1, I_1 and I_2:

$$dI_1 = (\lambda \overline{\omega_{11}} + d\lambda)\lambda^{-1} I_1 + \lambda \overline{\omega_{12}}(\lambda I_2 - \mu \lambda^{-1} I_1).$$

Now, by comparing this equation with the second equation in equation set (S8), we get that $\omega_{12} = \lambda^2 \overline{\omega}_{12}$. Then, by using the definitions $b_1 = \omega_{12}/\omega_1$ and $\overline{b}_1 = \overline{\omega}_{12}/\overline{\omega}_1$, we obtain the formula

$$b_1 = \lambda^3 \overline{b}_1.$$

So, except for the case that $b_1 = 0$, i.e., the case corresponding to an inflection point, there is only one real value of λ such that $\overline{b_1} = 1$. Note that the right λ for obtaining $\overline{b_1} = 1$ is computed using b_1 which depends only on the 2-jet of Γ. The consequence is the possibility of choosing a frame that depends on the 2-jet of the curve so that the quantity of turning is equal to 1. Following this, the condition $\lambda = 1$ is able to fix the frame of order 2. Accordingly the group of ambiguity at order two is the group $SG_{(2)}$ of upper triangular real 2 by 2 matrices with a diagonal $(1, 1)$:

$$SG_{(2)} = \left\{ \begin{pmatrix} 1 & \mu \\ 0 & 1 \end{pmatrix} \right\}. \tag{S11}$$

Now, to construct the set of moving frames of order 3, let us consider a field of frames R, where all along the curve segement we have guaranteed $b_1 = 1$. As differential calculus easily shows, the infinitesimal equation, near the identity matrix for the subgroup $SG_{(2)}$ inside $SG_{(1)}$, is $\omega_{11} = 0$. So let us define the new quantity $c_2 = \omega_{11}/\omega_1$. We can interpret c_2 as the "third order quantity of turning" along Γ. We will show that there exist frames $\overline{R}$ determined by the 3-jet of Γ such that $b_1 = 1$ and also $c_2 = 0$. Let us look, as before, at the change of frames along Γ from $R = (I_1, I_2)$ to $\overline{R} = (J_1, J_2)$. Now we have more constraints:

$$\begin{aligned} I_1 &= J_1 \\ I_2 &= J_2 + \mu J_1 \end{aligned} \tag{S12}$$

With the same notations as in (S8) and (S10) and the same procedure we used in order 2 we find:

$$\begin{aligned} \omega_1 &= \overline{\omega}_1 \\ \omega_{11} &= \overline{\omega}_{11} - \mu \overline{\omega}_{12}. \end{aligned} \tag{S13}$$

From this and the constraint of order one, i.e. $\overline{\omega}_{12} = \overline{\omega}_1$, it follows that:

$$c_2 = \overline{c}_2 - \mu$$

Remark: the right μ to obtain $\overline{c_2} = 0$ depends on the value of c_2 which was computed from third order contacts on Γ.

Thus, it is always possible to obtain $\overline{c}_2 = 0$ by adapting the "shearing" coefficient μ. Accordingly, a frame of order 3 will be a frame of order 2 such that $c_2 = 0$. The group $SG_{(3)}$ measuring the ambiguity of the frame at the order 3 is reduced to the Identity. Thus, the resulting frame of order 3 is unique, it is called the equi-affine Frenet-Serret frame.

Note that already at order 2, the form ω_1 is well defined and gives the equi-affine parameter σ_1, up to an additive constant through $\omega_1 = d\sigma_1$. In addition, $b_1 = \omega_{12}/\omega_1 = 1$, hence, $\omega_{12} = d\sigma_1$. From the condition of order 3 we get that $c_2 = \omega_{11}/\omega_1 = 0$, hence, $\omega_{11} = 0$. From the condition $\omega_{11} + \omega_{22} = 0$ we get that $\omega_{22} = 0$. In conclusion:

$$\begin{aligned} dM &= I_1 d\sigma_1 \\ dI_1 &= I_2 d\sigma_1 \\ dI_2 &= I_1 \omega_{21} \end{aligned} \tag{S14}$$

The first invariant appears at order 4. It is defined as $k_1 = \omega_{21}/\omega_1$ and is named the equi-affine curvature. All higher order invariants are obtained though the differentiation of k_1.

From equation set (S14) the infinitesimal variation of the equi-affine Frenet frame is given by

$$\begin{aligned} \frac{dM}{d\sigma_1} &= I_1 \\ \frac{dI_1}{d\sigma_1} &= I_2 \\ \frac{dI_2}{d\sigma_1} &= k_1 I_1. \end{aligned} \tag{S15}$$

Note that the sign of k_1 is independent of the orientation of Γ: if σ_1 is replaced by $-\sigma_1$, the tangent vector I_1 is replaced by $-I_1$, the acceleration vector I_2 is unchanged and so the value of k_1 does not change.

If $k_1 < 0$ the osculating conic is an ellipse, if $k_1 > 0$ it is a hyperbola, if $k_1 = 0$ it is a parabola.

Remark: as described by Blaschke [7], the parameter σ_1 is the one for which the parallelogram generated by the velocity and acceleration vectors has an area of 1. It can be recovered from any other parameterization by taking the integral of the third root of the absolute value of $x'y'' - y'x''$, the Euclidian curvature.

To compute σ_1, let us start with an Euclidian Serret-Frenet frame (J_1, J_2), where $\overline{b_1} = \kappa = R^{-1}$.

According to what we have already seen, to obtain a frame $I_1 = \lambda J_1, I_2 = \lambda^{-1}(I_1 + \mu J_2)$ with $b_1 = 1$, it is necessary and sufficient to verify that $\lambda^3 \overline{b_1} = 1$, then $\lambda = \sqrt[3]{R}$ is a good choice. This gives the 2/3 power law:

$$\frac{ds}{d\sigma_1} = R^{1/3}$$

It is not difficult to compute k_1, the equi-affine curvature, from the Euclidian frame:

$$k_1 = R^{-1/3}\left[\frac{1}{3}\frac{d^2R}{ds^2} - \frac{1}{R}\left[1 + \frac{1}{9}(\frac{dR}{ds})^2\right]\right]$$

For an ellipse k_1 is a negative constant and the total area enclosed by the ellipse equals $\pi(-k_1)^{-3/2}$. This can easily be proved by transforming the ellipse into a circle $R = constant$ by an area-preserving transformation.

If we start with an ordinary Cartesian frame $\overline{R}$ where the curve Γ is written as $y = f(x)$, the privileged point being O, such that $f(O) = 0$, $f'(O) = 0$ and $f"(O) > 0$ we have:

$$\overline{\omega_1} = dx, \overline{\omega}_{11} = \overline{\omega}_{22} = 0, \overline{\omega}_{12} = f''(O)dx, \overline{\omega}_{21} = 0.$$

At the order 4, we find (cf. Cartan):

$$k_1 = \frac{1}{2}(f''^{-2/3})''(O).$$

And then the reduced equation for any curve in the equi-affine Frenet frame is:

$$y_1 = \frac{1}{2}x_1^2 - \frac{1}{8}k_1 x_1^4 - \frac{1}{40}\frac{dk_1}{d\sigma_1}x_1^5 + ... \tag{S16}$$

Now let us study the main group G of all affine transformations. Recall that, when a system of affine coordinates (x, y) is given, an affine transformation is written as:

$$\begin{aligned} X &= ax + by + u \\ Y &= cx + dy + v \end{aligned} \tag{S17}$$

where a, b, c, d, u, v are specified real numbers such that $ad - bc \neq 0$. As for the Euclidian and equi-affine

groups, at order 0 we fix the moving frame $F_0 = (O, e_0, f_0)$. Once F_0 is fixed, a good choice for the first ambiguity subgroup $G_{(0)}$ is again the stabilizer of O. By fixing the origin it satisfies the condition $u = v = 0$. The group $G_{(0)}$ is the full real linear group $GL_2(\mathbb{R})$:

$$G_{(0)} = GL_2(\mathbb{R}) = \left\{ \begin{pmatrix} a & b \\ c & d \end{pmatrix} \middle| ad - bc \neq 0 \right\}. \tag{S18}$$

Clearly there is no invariant of order 0.

At order 1, as in the equi-affine case, $G_{(1)}$ is chosen as the stabilizer of the axis in F_0 sent to the tangent direction. As in the equi-affine case we obtain $c = 0$. For convenience let us set $a = \lambda, d = \tau^{-1}$ and $b = \mu\tau^{-1}$:

$$G_{(1)} = \left\{ \begin{pmatrix} \lambda & \mu/\tau \\ 0 & 1/\tau \end{pmatrix} \middle| \lambda\tau \neq 0 \right\}. \tag{S19}$$

Between two frames of first order (M, I_1, I_2), (M, J_1, J_2) attached at M_0 to a given curve Γ, there is a transformation in $G_{(1)}$:

$$\begin{aligned} I_1 &= \lambda J_1 \\ I_2 &= \frac{1}{\tau}(J_2 + \mu J_1) \end{aligned} \tag{S20}$$

Interesting things begin at the second order. Suppose we have a smooth field $R = (M, I_1, I_2)$ of coordinate frames along Γ, all being of first order; that is, for each point of Γ near the point M_0, the vector I_1 is along the tangent axis. As we have seen in the equi-affine case, the movement of R at the first order is defined by the *moving frame equations* (S8). As above in the equi-affine case, we denote by b_1 the function on Γ which is equal to ω_{12}/ω_1. It will also serve here as the measure of turning in the chosen field of frames.

Let $\overline{R}$ be another field of frames of order 1 for the same Γ, $\overline{R} = (M, J_1, J_2)$, which gives the forms $\overline{\omega}_1, \overline{\omega}_{11}, ..., \overline{\omega}_{22}$. Repeating the calculations of the second order in the equi-affine case, with $d = 1/\tau$ instead of $1/\lambda$, we obtain in the affine case:

$$b_1 = \lambda^2 \tau \bar{b}_1. \tag{S21}$$

Then, if M is not an inflection point, i.e. if $b_1 \neq 0$, it is always possible to choose λ and τ such that $\overline{b}_1 = 1$. We define $G_{(2)}$ in $G_{(1)}$ by the condition: $\lambda^2 \tau = 1$. Hence $G_{(2)}$ is the group of 2×2 matrices of the form

$$\begin{pmatrix} \lambda & \mu\lambda^2 \\ 0 & \lambda^2 \end{pmatrix}.$$

The group $G_{(2)}$ is richer than in the equi-affine case, being isomorphic to the affine group on a line. It measures the ambiguity at a second order on the pure affine frame. There is no invariant of order 2, and $G_{(2)}$ is the inertia group at the order 2.

For order 3 we repeat the operation with paths of frames $R = (I_1, I_2), \overline{R} = (J_1, J_2)$ of second order, that is along Γ we assume that the equations: $\omega_{12} = \omega_1$ and $\overline{\omega}_{12} = \overline{\omega}_1$ are satisfied. Let us define a new quantity b_2:

$$b_2 = \frac{\omega_{11} - \frac{1}{2}\omega_{22}}{\omega_1}.$$

This quantity describes the residual velocity of the frame R modulo the action of $G_{(2)}$.
Between R and $\overline{R}$, another easy calculation gives

$$\begin{aligned} \lambda\omega_1 &= \overline{\omega}_1, \\ \omega_{11} &= \overline{\omega}_{11} + \frac{d\lambda}{\lambda} - \overline{\omega}_{12}\mu, \\ \omega_{22} &= \overline{\omega}_{22} + 2\frac{d\lambda}{\lambda} + \overline{\omega}_{12}\mu. \end{aligned}$$

Hence,

$$b_2 = \lambda\overline{b_2} - \frac{3}{2}\lambda\mu. \tag{S22}$$

Here, as in the equi-affine case, no restriction is necessary; it is always possible to choose the value of μ such that $\overline{b_2} = 0$. Then we define a frame of order three by the condition $b_2 = 0$. Any transformation between two such frames must satisfy $\mu = 0$; hence we define $G_{(3)}$ in $G_{(2)}$ by the equation $\mu = 0$:

$$G_{(3)} = \left\{ \begin{pmatrix} \lambda & 0 \\ 0 & \lambda^2 \end{pmatrix} \right\}.$$

Such transformations are isomorphic to the group $\mathbb{R}^*$. However, observe that the operation on the plane

is not through ordinary dilatation. The tangential and normal affine directions are not submitted to the same scaling law: the distance in the normal direction scales as the square root of the distance in the tangent direction.

There is no invariant of order 3. We have two conditions on the infinitesimal motions of frames of order 3: $\omega_{11} = \frac{1}{2}\omega_{22}$ and $\omega_{12} = \omega_1$.

At order 4 we define the last quantity of change as

$$b_3 = \frac{\omega_{21}}{\omega_1}.$$

Under an element of $G_{(3)}$ as transformation from R to $\overline{R}$ we get $\omega_{21} = \lambda\overline{\omega}_{21}$. Hence:

$$b_3 = \lambda^2\overline{b_3}. \tag{S23}$$

Then there are three possibilities for b_3:

1. $b_3 > 0$, we can adapt λ such that $\overline{b_3} = +1$; we call this case a hyperbolic point.
2. $b_3 < 0$, we adapt λ such that $\overline{b_3} = -1$; this is the case of an elliptic point.
3. $b_3 = 0$, this is the degenerate case of a parabolic point.

In a frame of order 4 we impose $b_3 = \varepsilon$, with $\varepsilon = -1, +1$ or 0.

The two cases $b_3 = -1$ and $b_3 = +1$ are intrinsically different from the geometrical point of view: in the elliptic case the osculating conic is an ellipse and in the hyperbolic case it is an hyperbola. Naturally for $b_3 = 0$ it is a parabola, but here λ continues to be arbitrary. Such a special point must be separately analyzed.

For $\varepsilon = \pm 1$ the group $G_{(4)}$ reduced to $(\lambda = \pm 1, \tau = 1)$ is the true inertia of order 4:

$$G_{(4)} = \left\{ \begin{pmatrix} \pm 1 & 0 \\ 0 & 1 \end{pmatrix} \right\}.$$

The main conclusion is that for each point on an oriented curve there is a canonical Frenet frame of order 4. In particular, the vector I_1 is imposed, and ω_1 is also fixed. This automatically gives a local privileged parameter - the purely affine arc-length along Γ. We denote it by σ. The condition for this numerical function on Γ is $\omega_1 = d\sigma$, so σ is well defined only up to an additive constant.

All frames of order 5 and more coincide with this frame but, starting with order 5, invariants start to exist. The first invariant is of order 5 and is called the *affine curvature*:

$$K = \frac{\omega_{11}}{\omega_1} \tag{S24}$$

At order 6 or higher, all local invariants of Γ modulo G are deducible from the derivatives of the function $K(\sigma)$ and from the type of b_3, i.e., being elliptic or hyperbolic. Then the equations of the purely affine moving frame are:

$$\begin{aligned} \frac{dM}{d\sigma} &= I_1 \\ \frac{dI_1}{d\sigma} &= KI_1 + I_2 \\ \frac{dI_2}{d\sigma} &= \varepsilon I_1 + 2KI_2. \end{aligned} \tag{S25}$$

The link between affine and equi-affine Frenet frames is very simple:
the affine basis vectors I_1, I_2 have the same directions as the equi-affine basis vectors J_1, J_2, and the ratios $I_1/J_1 = \gamma$, $I_2/J_2 = \nu$ satisfy the fundamental relations:

$$\nu = \gamma^2, \tag{S26}$$

$$\frac{1}{\gamma}\frac{d\gamma}{d\sigma} = K. \tag{S27}$$

Such a function γ, determined up to a multiplicative constant, can be called an *affine gain factor.* An easy identification of frames shows that γ^{-2} is a constant multiple of $|k_1|$.
For the canonical parameter σ, this implies the modified 2/3 power law:

$$\frac{d\sigma}{ds} = R^{-1/3}|k_1|^{1/2}. \tag{S28}$$

This gives the following relations between K and k_1 :

$$K = -\frac{1}{2}\frac{1}{k_1}\frac{dk_1}{d\sigma} = \frac{d}{d\sigma_1}(\frac{1}{\sqrt{k_1}}).$$

Let a point O be specified on the oriented curve Γ and consider the coordinates (x, y) for $\mathbb{A}$ within the canonical frame $(I_1(0), I_2(0))$. Let us choose the parameter σ with $\sigma = 0$ at O. Then we easily deduce from the system (S25), the parameterized equation of Γ in the neighborhood of O:

$$\begin{aligned} x(\sigma) &= \sigma + \frac{1}{6}(\varepsilon + K^{'} + K^2)\sigma^3 + \frac{1}{24}(4\varepsilon K - 3KK^{'} + K^{''} + K^3)\sigma^4 + \dots \\ y(\sigma) &= \frac{1}{2}\sigma^2 + \frac{1}{2}K\sigma^3 + \frac{1}{24}(4K^{'} + 7K^2 + \varepsilon)\sigma^4 + \dots, \end{aligned} \tag{S29}$$

which gives the following implicit equation up to order 5 in the affine canonical frame:

$$y = \frac{1}{2}x^2 + \frac{1}{2}Kx^3 + \frac{1}{8}(K^2 - \varepsilon)x^4 + \dots \tag{S30}$$

These equations are not the simplest possible ones in an affine frame, because the reduced equi-affine form (S16) is apparently simpler. The affine arc-length σ is more natural than σ_1 because a curve and its image through an affine transform g do not have the same parameter σ_1, the latter being true only when $det(g) = \pm 1$. In general, σ_1 is transformed by a one dimensional affine change $a\sigma_1 + b$. If we privilege the equi-affine subgroup, without choosing a unit of area, all that we do has a pure affine significance but σ_1 is only defined up to an affine function, as in $t = a\sigma_1 + b$. Once a unit of area is chosen, the constant $1/a$ may be called an *equi-affine gain factor*, because in the case of a Euclidian plane the parameter t gives a Euclidian velocity of size $a^{-1}R^{1/3}$.

Instead of restricting the allowed transformations we can extend them by invoking the projective group PGL_3. this group does not act linearly but through homographic functions of affine coordinates (so, to properly define its action we must add a circle of points at infinity to the affine plane):

$$\begin{aligned} X &= \frac{ax + by + u}{ex + fy + w}, \\ Y &= \frac{cx + dy + v}{ex + fy + w}. \end{aligned} \tag{S31}$$

Working with this larger geometry, it is possible to obtain an even simpler reduced equation (valid for non-sextatic points). Here the Halphen' invariant of order 7, denoted by k, which is also called projective curvature, appears and:

$$Y = \frac{1}{2}X^2 - \frac{1}{20}X^5 + \frac{k}{280}X^7 + \dots$$

A.3 Degeneracies

As we shall see, near inflection points the affine arc-length σ tends to infinity like the logarithm of s. Thus, if we persist with the choice of time being proportional to σ, i.e. a constant speed from the affine point of view, an infinite time is necessary to reach the singular point (as if Zeno were right in his paradox, and Achilles, who naturally is very affine, could have never attained the turtle). Near parabolic points we see that the inverse happens; σ tends to zero as $s^{3/2}$. That is, if we persist with the choice that time is proportional to σ, the speed will tend to infinity (like $s^{-1/3}$) when approaching the parabolic point (and even Achilles cannot reach such a speed, so Zeno would have again been right).

Regarding the equi-affine parametrization σ_1, we see that the situation is totally different. At inflection points the speed tends to infinity like a constant time $s^{-1/3}$, and at parabolic points, of course, there is no singularity at all. At these points equi-affine geometry applies regularly.

Near a non-degenerate inflection point the radius of curvature R admits an asymptotic expansion:

$$R = Cs^{-1} + C_0 + C_1 s + C_2 s^2 + ...$$

From the expression of k_1 as a function of R and its successive derivatives, we easily compute the asymptotic development:

$$k_1 = \frac{5}{9}C^{2/3}s^{-8/3}[1 - \frac{2}{15}\frac{C_0}{C}s + (\frac{4}{15}\frac{C_1}{C} - \frac{2}{45}\frac{C_0^2}{C^2})s^2 + ...].$$

Note that the constant C is strictly positive, then $k_1 > 0$ is also strictly positive, and thus, near an inflection point we are automatically inside the hyperbolic regime.

From the formula of the canonical affine velocity, $\frac{ds}{d\sigma} = R^{1/3}|k_1|^{-1/2}$, we obtain the asymptotic expansion:

$$\frac{ds}{d\sigma} = \frac{3s}{\sqrt{5}}[1 + \frac{2}{5}\frac{C_0}{C}s + (\frac{1}{5}\frac{C_1}{C} - \frac{3}{50}\frac{C_0^2}{C^2})s^2 + ...].$$

Here we see that the parameter σ has a logarithmic divergence: $\sigma \sim \frac{\sqrt{5}}{3}\log(\frac{1}{s})$ and the following asymptotic of the full affine curvature K holds:

$$K = \frac{4}{\sqrt{5}}(1 + \frac{9}{20}\frac{C_0}{C}s + ...)$$

Suppose now that we are working with the equi-affine parametrization of Γ with units of area coming from the metric. Then, as we approach the inflection point, the speed satisfies:

$$\frac{ds}{d\sigma_1} = R^{1/3} \sim C^{1/3}s^{-1/3}$$

We see that contrary to affine velocity, the equi-affine velocity tends to infinity at an inflection point.

We observe that the equation

$$dt = (d\sigma)^{1/4}(d\sigma_1)^{3/4}$$

gives

$$\begin{aligned}\frac{ds}{dt} &= (\frac{ds}{d\sigma})^{1/4}(\frac{ds}{d\sigma_1})^{3/4} \\ &\sim (\frac{3s}{\sqrt{5}})^{1/4}(C^{1/3}s^{-1/3})^{3/4} \sim (\frac{3C}{\sqrt{5}})^{1/4}.\end{aligned}$$

Consequently $\frac{ds}{dt}$ is a regular function. We see that, when we choose $dt = d\sigma^{1/4}d\sigma_1^{3/4}$ we obtain a parametrization of inflection points without singularity. Moreover, it is a well defined equi-affine invariant parameterization.

B First test: elliptical trajectories

B.1 Data recording and processing

Three subjects (two right-handed, one left-handed, all males aged 28-31) volunteered for this experiment. None reported any previous hand injuries and all gave their informed consent prior to their inclusion in the study. They were instructed to continuously trace ten different elliptical paths. The shapes of these ellipses were; (a,b) = (49,46), (49,27), (49,5), (166,156), (166,90), (166, 17) (283,267), (283,154), (283,28) and (408,287), where 'a' and 'b' mark the minor and major half-axes of ellipse, respectively, and the units are in mm. The ellipses to be traced were drawn on white sheets placed on the table in front of the subjects. Each ellipse was traced at three different speeds: slow, natural and fast. Each elliptical trajectory was traced 13 times and the movements recorded using a WACOM Intuos A3-size graphics table (model GD-1218-R) with a spatial accuracy of 0.25 mm and a temporal resolution of 200 Hz.

The 'x' and 'y' coordinates of the subjects' tracings were separately approximated by a Fourier series containing the coefficients of up to 4.5 Hz, where 95% of the power spectrum of the data was below 4.5 Hz. To obtain fully repetitive movements (i.e., to fully complete the elliptical curves), a second order polynomial was added to each approximation.

These approximations were then analytically differentiated to obtain the speed, curvature and any other desired kinematic variable.

B.2 Theoretical and experimental testing of the law of area

Both the 2/3 power law and the isochrony principle have been thoroughly tested for handwriting and drawing movements [8–13]. However, it is not clear whether these two descriptions have a similar origin, since the 2/3 power law is a local kinematic constraint while the isochrony principle refers to a more global property.

In testing the 2/3 power law, one examines the relationship between local speed V and local radius of curvature R:

$$\log V = \log \gamma + \beta \log R. \quad \text{(S32)}$$

On the other hand, to assess the tendency towards isochrony, the link between the mean gain factor γ

and the global Euclidian perimeter P is usually examined:

$$\log \gamma = C + \alpha \log P. \tag{S33}$$

For ellipses, γ was claimed to be nearly constant during an entire period, β was nearly 1/3 and perfect isochrony corresponded to $\alpha = 2/3$ (cf. [12]).

Even after associating the empirical 2/3 power law and equi-affine geometry [14–16], the connection between the 2/3 power law and global isochrony was still not understood. *A priori* the equi-affine hypothesis predicts only that γ is constant along an ellipse but states nothing about its magnitude. This is precisely where the full affine treatment makes a new contribution: for ellipses it imposes the values of both α and β at the same time. For any curve it stipulates the dependence of the gain factor on equi-affine curvature, namely: $\gamma = C_0|k_1|^{-1/2}$. On the other hand, if an ellipse encloses a total area A, and since for ellipses $k_1 = CA^{-2/3}$, the full affine model and consequently global isochrony imply that:

$$\gamma = C' A^{1/3}.$$

Since the area A enclosed by the ellipse is proportional to P^2, where P is the ellipse's perimeter, we obtain (S33) with $\alpha = 2/3$. Equation (S33) matches the empirical observations of Lacquaniti et al. [8] for elliptical drawings. Furthermore, Viviani and Cenzato [12] successfully accounted for (S33) by joining the 2/3rd power law with perfect isochrony and showing that for an ellipse $\alpha = 2/3$. But the main advantage of our affine treatment is in liberating us from the Euclidian quantity P. Now, the simplest law that we examine is better expressed by:

$$\log \gamma = \log C' + \frac{1}{3} \log A,$$

where A is the total area enclosed by the ellipse.

In Figure S1 we show the results of $Log\gamma$ versus $LogA$.

Interpreting Figure S1 and table 1 in the main paper we observe the following:

a) The slopes of the regression lines ranged from 0.12 to 0.18 for S1 and from 0.17 to 0.23 for S2. For the 3rd subject the value of the slope was closer to 0.33. This subject traced the ellipses significantly faster

for all prescribed drawing speeds.
b) The intercepts, λ, for S1 and S2 were also very close to each other for all speeds and increased monotonically with speed.
c) The coefficient of determination, R^2 remained nearly the same over changes in speed for all the subjects, being higher for S2 and S3 than for S1.

All these results provide a direct test of the affine invariance and allow us to specifically state what is global isochrony. Let us remark that *Isochrony* does not mean that different curves must be drawn taking the same total movement duration. Such a behavior is only expected for curves which fully transform, one into the other, through affine transformations. While any ellipse is the affine transform of any other ellipse, *a priori* when the subjects deformed the curves they deformed them similarly across different speeds and this is therefore consistent with global isochrony.

B.3 Limits of isochrony

Following the above, we also looked for direct relationships between the total movement durations T for the execution of the ellipses, their total perimeters marked by P and their eccentricities marked by ϵ. For an ellipse of perimeter P and eccentricity ϵ, and taking into account the 2/3 power law plus global isochrony, it was analytically shown [12] that:

$$\log \gamma = C + \frac{2}{3} \log P + f(\epsilon). \tag{S34}$$

Since the function $f(\epsilon)$ is a mildly varying function of ϵ, the ellipse's eccentricity, this is approximated by equation S33 above with $\alpha = 2/3$ (see [11]).

Consider an ellipse

$$\frac{x^2}{a^2} + \frac{y^2}{b^2} = 1,$$

and write $x = a \cos \varphi$, $y = b \sin \varphi$; if s denotes the curvilinear Euclidian distance, we have

$$\frac{ds^2}{d\varphi^2} = a^2 \cos^2 \varphi + b^2 \sin^2 \varphi = a^2(1 - \epsilon^2 \sin^2 \varphi)$$

where ϵ is the eccentricity and φ is the angle.

Then the corresponding arc-length for the elliptical section between $\varphi = 0$ and φ is:

$$\Delta s = a \int_0^{\varphi} \sqrt{1 - \epsilon^2 \sin^2 \varphi} d\varphi.$$

Now, dividing Δs by a gives the definition of the Legendre integral of second species: $E(\varphi;\epsilon)$. When the superior limit of the integral is taken as $\pi/2$, it is called the complete integral of the second kind and is denoted by $E(\epsilon)$. It measures a quarter of the perimeter P divided by the semi-axis a.

Using Wallis formulae [17], it is easy to expand E in the powers of ϵ:

$$E = \frac{\pi}{2}\left[1 - \sum_{n=1}^{n=\infty}\left(\frac{1\cdot 3\cdot 5\cdot \ldots \cdot (2n-1)}{2\cdot 4\cdot 6\cdot \ldots \cdot 2n}^2\right)\frac{\epsilon^{2n}}{2n-1}\right].$$

The area A of the ellipse is equal to $\pi ab = \pi a^2\sqrt{1-\epsilon^2}$, and the gain factor γ given by affine geometry equals $CA^{1/3}$, so

$$\begin{aligned}\log\gamma &= \log(C') + \frac{2}{3}\log(a) + \frac{1}{2}\log(1-\epsilon^2)\\ &= \log(C") + \frac{2}{3}\log(P) - \log(E/\sqrt{1-\epsilon^2}).\end{aligned}$$

This provides an exact expression for the function f in the formula of Viviani and Cenzato (see equation (S34) above). And from the expansion of E we obtain a testable expansion and estimation of f. However, what Viviani and Schneider really found was that when P increases, the empirically based expression for the dependence of movement duration T on the perimeter of the ellipse P is:

$$\log T = C + \eta \log P, \tag{S35}$$

with the mean value $\eta = 0.4$. This indicates a real departure from perfect isochrony, namely from $\eta = 0$.

Here we propose a different formula relating $\log T$ to $\log P$. This is a non-linear formula, expressing that when P grows too much, an Euclidian behavior competes with the affine one and plays a progressively increasing role. This is consistent with several ellipses we examined by looking at $\log v$ versus $\log\kappa$ along ellipses (Figure 1 in the main paper).

C Global scaling

While we mostly examined elliptical and more complex drawing movements, here we wish to briefly examine hand scribbling movements for which complete preplanning is unlikely. For such motions the following empirical law for the mean value of gain factor $\bar{\gamma}$ and the radius r of the frame within which the subject was producing the continuous scribbling was described [11]:

$$\log \bar{\gamma} = 0.701 + 0.634 \log r. \tag{S36}$$

This observation agrees completely with the pure affine rule, since affine invariance predicts that γ scales as $R^{2/3}$, where R denotes the local radius of curvature. Now it is reasonable to expect that R will scale as the frame radius r under dilatation. This, however, does not mean at all that there are no equi-affine (or parabolic) or straight segments among such scribbling motions. To the contrary, their presence is expected, but as is explained in section E.1 through continuity plus monotonicity the speeds for such segments can scale similarly to the speed of affine segments. Thus, the fact that we have full-affine segments is expected to affect the behavior in all segments.

D Second test: complex forms: velocity prediction

D.1 Data recording and approximation

In the drawing experiment (from [18]) three subjects were asked to repeatedly draw each figural form (see Figure S2) for 10 seconds on a digitizing tablet (Numonics Corporation, Montgomeryville, PA; Model 2200-0.60TL.F). Each subject performed three such 10 sec trials.

The locomotion data were recorded using an optoelectronic video motion capture device (Vicon V8, Oxford Metrics Ltd.) consisting of 13 cameras. A set of light-reflective markers were placed on the subjects' bodies. The data were recorded at a sampling rate of 60Hz. That is, the time interval between any two adjacent data samples was $dt = 0.01667$ seconds. Two reference points, the M-point and the R-point, represented the location of the subject in the 2D plane (see Figure S3).

D.2 Curvature and velocity computations

To compare different velocity profiles along the same path, we compared the tangential velocities at the same position on the curve. For this purpose, we calculated the velocities at points along the trajectory $\gamma(r) = \big(f_x(r), f_y(r)\big)$ which were situated at a constant Euclidian distance from each other. We marked these points by $S = [0 : ds_m : S_m]$, where S_m is the total Euclidian arc-length and ds_m is the constant distance between consecutive points along S for a given trial m. We calculated the time stamps between points in S in the experimental parameterization by interpolation. This does not change the parameterization. The only difference between the points $s \in S$ and $t \in T$ is the samples that we use. The derivative of the function, dr/dt, is calculated on the points $s \in S$ by spline interpolation.

The experimental velocity is calculated as

$$v(s) = \frac{dr}{dt}(s) \cdot \sqrt{\Big(\frac{df_x}{dr}\Big|_{r=r(s)}\Big)^2 + \Big(\frac{df_y}{dr}\Big|_{r=r(s)}\Big)^2}$$

where $s \in S$.

The absolute Euclidian and equi-affine curvatures are calculated at points along S using the standard equations:

$$R^{-1}(s) = \kappa(s) = \left| \frac{\frac{df_x}{dr} \cdot \frac{d^2 f_y}{dr^2} - \frac{d^2 f_x}{dr^2} \cdot \frac{df_y}{dr}}{\Big(\big(\frac{df_x}{dr}\big)^2 + \big(\frac{df_y}{dr}\big)^2\Big)^{3/2}} \right|$$

and

$$\kappa_1(s) = \left| \frac{4\Big(\frac{d^2 f_x}{dr^2} \cdot \frac{d^3 f_y}{dr^3} - \frac{d^3 f_x}{dr^3} \cdot \frac{d^2 f_y}{dr^2}\Big) + \Big(\frac{df_x}{dr} \cdot \frac{d^4 f_y}{dr^4} - \frac{d^4 f_x}{dr^4} \cdot \frac{df_y}{dr}\Big)}{3\Big(\frac{df_x}{dr} \cdot \frac{d^2 f_y}{dr^2} - \frac{d^2 f_x}{dr^2} \cdot \frac{df_y}{dr}\Big)^{5/3}} - \frac{5\Big(\frac{df_x}{dr} \cdot \frac{d^3 f_y}{dr^3} \; \frac{d^3 f_x}{dr^3} \cdot \frac{df_y}{dr}\Big)^2}{9\Big(\frac{df_x}{dr} \cdot \frac{d^2 f_y}{dr^2} - \frac{d^2 f_x}{dr^2} \cdot \frac{df_y}{dr}\Big)^{8/3}} \right|$$

where all the derivations are taken at the point $r(s)$.

The Euclidian, equi-affine and affine velocities at the S points are calculated according to equation 7 in the main paper. For the calculation of the minimum-jerk velocity we used the Matlab function "lsqnonlin". As input the function receives any parameterization and a function calculating the jerk for that parameterization. The output of this function is the parameterization with the smallest jerk at the points of S.

D.3 Geometrically combined velocity

Using equations 7 in the main paper, the theoretical velocity is defined by the function

$$\begin{aligned} v_T(s) &= v_0(s)^{\beta_0(s)} \cdot v_1(s)^{\beta_1(s)} \cdot v_2(s)^{\beta_2(s)} = \\ &= {C_0}^{\beta_0(s)} \cdot {C_1}^{\beta_1(s)} \cdot {C_2}^{\beta_2(s)} \cdot \kappa(s)^{-\frac{\beta_0(h)+\beta_1(s)}{3}} \cdot \kappa_1(s)^{-\frac{\beta_0(s)}{2}} \end{aligned} \tag{S37}$$

where β_0, β_1 and β_2 are continuous functions, such that $\beta_0(s) + \beta_1(s) + \beta_2(s) = 1$ for all s and C_0, C_1 and C_2 are the constant affine, equi-affine and Euclidian velocities, respectively.

Finding the β functions

Here we describe in greater detail the method we have developed to calculate the values of the different β functions.

Equation (S37) can be represented in the logarithmic space as:

$$\log v_T = c + (1 - \beta_2)\log(\kappa^{-1/3}) + \beta_0 \log(\kappa_1^{-1/2}) \tag{S38}$$

where $c = \log({C_0}^{\beta_0} \cdot {C_1}^{\beta_1} \cdot {C_2}^{\beta_2})$.

Then, the functions $\beta_0(s)$, $\beta_1(s)$ and $\beta_2(s)$ are constant along a segment L of the curve $\gamma(s)$ if and only if the curve
$\delta(s) = \big(\log v_e(s), \log(\kappa(s)^{-1/3}), \log(\kappa_1(s)^{-1/2})\big)$ on L represents a straight line in $\mathbb{R}^3$ with slopes $1 + \beta_2$ and β_0, where v_e is the experimental velocity.

We can construct the β functions by finding the values of the functions on all the segments for which the β-s are constant and when two nearby segments are connected using a smooth interpolation. We call this technique of constructing the β functions "AvEAvE".

However, in many cases there are only a very small number of segments for which all the β-s are simultaneously constant. We estimate the values of the β-s only by connecting two constant segments that may be far apart from each other. This leaves us with a large number of curve segments for which we do not have any information.

Another option is to assume that one of the β-s equals zero on the entire curve and to look for segments where the other two β-s are constant.

Using $\beta_2 = 0$ in equation (S38), we get

$$\log \frac{v_{T_2}}{\kappa^{-1/3}} = c_2 + \beta_0 \log(\kappa_1^{-1/2})$$

where $c_2 = \log({C_0}^{\beta_0} \cdot {C_1}^{\beta_1})$.

Hence, we can find all the segments, L_2, where the curve $\delta_2(s) = \big(\log(\frac{v_e(s)}{\kappa(s)^{-1/3}}), \log(\kappa_1(s)^{-1/2})\big)$ represents a straight line in $\mathbb{R}^2$. The slope of δ_2 is the value of β_0 within the segment. Again, we estimate the value of β_0 on the rest of the curve by spline interpolation and $\beta_1 = 1 - \beta_0$. We call this technique of constructing the β functions "AvEA".

Using $\beta_1 = 0$ in equation (S38), we get

$$\log v_{T_1} = c_1 + \beta_0 \log(\kappa^{-1/3} \cdot \kappa_1^{-1/2})$$

where $c_1 = \log({C_0}^{\beta_0} \cdot {C_2}^{\beta_2})$. Hence, we can find all the segments, L_1, where the curve $\delta_1(s) = \big(\log(v_e(s)), \log(\kappa(s)^{-1/3}\kappa_1(s)^{-1/2})\big)$ represents a straight line in $\mathbb{R}^2$. The slope of δ_1 is the value of β_0 within the segment. Again, we estimate the value of β_0 on the rest of the curve by spline interpolation and $\beta_2 = 1 - \beta_0$. This technique of constructing the β functions we call "AvE".

By assuming that $\beta_0 = 0$ we obtain from equation (S38) the equation:

$$\log v_{T_0} = c_0 + \beta_1 \log(\kappa^{-1/3})$$

where $c_0 = \log({C_1}^{\beta_1} \cdot {C_2}^{\beta_2})$. Hence, we can find all the segments, L_0, where the curve $\delta_0(s) = \big(\log(v_e(s)), \log(\kappa(s)^{-1/3})\big)$ represents a straight line in $\mathbb{R}^2$. The slope of δ_0 is the value of β_1 within the segment. Again, we estimate the value of β_1 on the rest of the curve by spline interpolation and $\beta_2 = 1 - \beta_1$. This technique of constructing the β functions is called "EAvE".

The last technique uses the results from the combination of the two velocities to estimate the remaining β function. Equation (S37) can be written as

$$v_T(s) = v_{Ti}(s)^{1-\beta_i(s)} \cdot v_i(s)^{\beta_i(s)}$$

where v_{Ti} is the theoretical velocities calculated by the "AvEA" technique for $i = 2$, "AvE' technique for $i = 1$ and 'EAvE' technique for $i = 0$. To estimate the function $\beta_i(s)$ we look for segments of the curve $\delta_{Ci}(s) = \big(\log(\frac{v_e(s)}{v_{Ti}(s)}), \log(\frac{v_i(s)}{v_{Ti}(s)})\big)$ that represent a straight line in $\mathbb{R}^2$. The velocity profiles we obtain using this method we call 'Ev(AvEA)' when i=2, 'EAv(AvE)' when i=1 and 'Av(EAvE)' when i=0.

The constant velocities, C_0, C_1 and C_2 are considered constant throughout an entire trial. We choose those constants which give the best AIC with respect to the experimental velocity.

Finding segments of straight lines

Let $\delta(s) = \big(x(s), y(s)\big)$ be a curve in $\mathbb{R}^2$. Let L be the set of the segments in δ that represent straight lines.

As the first step for each point, $b \in \delta$, we calculate the best β values for the point within an interval of 25 points; that is by using a sliding window and associating the point b with 25 different intervals, each of a size 25 points. We choose β as the slope of the straight line which achieves the best R^2 score among these intervals when using a linear regression.

In order for a segment l to belong to L, all the points in l need to have a higher R^2 score than a threshold value of 0.97, and must be associated with a unique straight line, that is, have the same slope. The slope, $\beta(s)$, is considered the same for all the points belonging to l if $\frac{d\beta}{ds}$ for $s \in l$ is smaller than some threshold. The threshold for the sensitivity in the variation in β that we have employed was 0.05 for locomotion and 1.0 for drawing. Moreover, because the slope represents β_0, β_1 and β_2, it must lie within the interval $[0, 1]$. In this procedure we have only considered long enough segments l within L. A segment is considered long enough if it includes at least 30 points. In addition, two segments $l_1, l_2 \in L$ are replaced with one large segment, l, which is composed of both of them, if they are close enough to each other and of the difference between their slopes is small.

Handling singularities

A singularity is a point where the Euclidean curvature equals zero, that is an *inflection point* or a point where the equi-affine curvature equals zero, that is a *parabolic point.* Near an inflection point the equi-affine velocity tends to infinity and the affine velocity tends to zero. In this case, equation (S37) tends to zero or infinity in most of the combinations of the $\beta - s$. There are two cases where this does

not happen: when $\beta_2 = 1$ and $\beta_0 = \beta_1 = 0$ and when the affine and equi-affine velocities cancel each other out, i.e. when $\beta_0 = 0.25$ $\beta_1 = 0.75$ and $\beta_2 = 0$ (See section A.3). Near parabolic points the affine velocity tends to infinity and equation (S37) tends to infinity unless β_0 equals zero.

Considering that $\beta - s$ values are chosen only for parts of the curves and we interpolate their values in the rest of the curve, the values of the $\beta - s$ on singularities may be chosen depending on the segments around them. To prevent the theoretical velocity of equation (S37) from going to infinity or zero around singularities, the values of the $\beta - s$ near the inflection points are set $\beta_0 = 0.25$, $\beta_1 = 0.75$ and $\beta_2 = 0$, and near parabolic points we set β_0 to equal zero.

D.4 Expanded results

Figures S4 and S5 show comparisons between experimentally recorded and theoretically predicted trajectories for drawing and locomotion, respectively. Comparing the experimental velocity to the constant equi-affine velocity and to the constant affine velocity shows that, for parts of the curves, the experimental velocity follows the constant equi-affine velocity more closely, while in other parts it is more influenced by the constant affine velocity (see Figures S4 and S5, for examples). In addition, it can be seen that both of these velocities are necessary to describe the experimental velocity.

From equation (S37) we can see that the β functions describe the extent to which each geometry influences the combination velocity, v_T, at every point on the curve. Figures S6 and S7 display examples of the best combination velocity compared with the experimental velocity, i.e., the β functions giving the best AIC score for comparing the combination velocity and the experimental velocity using the methods described in section D.3 to construct the β functions. The colors on the path in Figures S6A, D, and G and S6A, D, and G represent the weights of the different geometries representing the changes in dominance of the various geometries along the different paths. The full range of colors and their relation to the values of β_0, β_1 and β_2 can be seen in Figure S8. Figure S6 gives the combination velocity and the β functions for one repetition of the different curves for the drawing data. Figure S7 shows the combination velocity and the β functions for one repetition of the different figural forms for the M-point of the locomotion data. The R^2 score for the combination velocity, calculated over the entire trial and not only for the displayed repetition, is marked on the velocity figures.

Exp	Shape	AIC of EA	AIC of A	AIC of Combination	AIC of Min-jerk	Probability Comb v Min-jerk
Drawing	$\mathcal{C}_1$	601±129	1414±231	528±171	634±145	1±0
	$\mathcal{C}_2$	703±64	1336±68	488±138	577±160	0.95±0.13
	$\mathcal{C}_3$	831±68	1521±150	755±280	692±179	0.55±0.52
	$\mathcal{L}_1$	1441±791	1507±836	1168±908	1259±882	0.78±0.44
	$\mathcal{L}_2$	1317±95	1195±94	819±124	940±160	0.78±0.44
	$\mathcal{L}_3$	1414±70	1182±89	787±224	1014±146	1.00±0.00
	$\mathcal{A}_1$	2206±70	2695±96	742±134	751±108	0.49±0.53
	$\mathcal{A}_2$	2206±74	2674±36	976±104	823±77	0.00±0.00
	$\mathcal{A}_3$	2274±70	2682±57	1205±88	992±110	0.00±0.00
Locomotion M-point	$\mathcal{C}$	4238±698	6083±1358	4203±632	4452±577	0.79±0.41
	$\mathcal{L}_1$	5211±821	6891±1065	4870±893	5434±750	0.93±0.27
	$\mathcal{L}_2$	5452±901	7969±1220	4950±1224	5813±815	0.91±0.28
	$\mathcal{L}_3$	6703±1326	8165±1637	6015±1373	6445±1209	0.73±0.45
	$\mathcal{A}_1$	6895±968	7596±962	5240±1151	5302±1093	0.56±0.50
	$\mathcal{A}_2$	6546±657	7350±797	4881±643	5024±505	0.67±0.47
	$\mathcal{A}_3$	6696±934	7582±1022	4706±694	5102±647	0.93±0.26
Locomotion R-point	$\mathcal{C}$	3959±493	5085±1175	3891±473	3906±477	0.70±0.48
	$\mathcal{L}_1$	4816±1464	5746±1251	4861±1311	5035±1176	0.89±0.33
	$\mathcal{L}_2$	5114±828	6863±1171	4718±674	5375±697	0.93±0.27
	$\mathcal{L}_3$	6282±1693	7586±1178	5313±1605	5710±1387	0.83±0.39
	$\mathcal{A}_1$	6485±1278	7163±1102	5126±1929	5382±1752	0.73±0.46
	$\mathcal{A}_2$	6148±618	7112±630	4200±876	4640±741	0.95±0.22
	$\mathcal{A}_3$	6398±1456	7016±1285	5382±2090	5476±1805	0.62±0.51

Table S1. *The AIC scores of the different models for the different shapes.*
The means and SD values of the AIC scores of the pure equi-affine and affine geometries, the minimum-jerk model and the combination of Euclidian, equi-afine and affine geometries model. The AIC score is based on the level of the error, hence, the lower the AIC score, the better the model. The probabilities for every figural form that the combined velocity model is better than the minimum-jerk model is calculated according to the equation: $p = e^{-0.5\Delta AIC}/(1 + e^{-0.5\Delta AIC})$.

Table S1 contains the AIC scores of the different models and shapes for the drawing data and for the locomotion data for both the M and R points. The AIC results and the mean of the different β functions for the R-point are shown in Figure S9. These results show no significant difference from the results obtained with the M-point.

We calculated the mean of the functions β_0, β_1 and β_2 for trials with good approximation of the recorded velocity by the combination velocity, i.e., $R^2 \geq 0.6$ (see Figures S9 here and 8 in the main paper). Comparing the means of β_2 for the drawing data versus the locomotion data for both the R and

	$\mathcal{C}$	$\mathcal{L}_1$	$\mathcal{L}_2$	$\mathcal{L}_3$	$\mathcal{A}_1$	$\mathcal{A}_2$	$\mathcal{A}_3$
β_0	*	*	*	*			
β_1			*	*	*	*	*
β_2	*	*	*	*	*	*	*

Table S2. *Significant differences between the β functions of drawing and locomotion.*
Stars represent the figural forms and β functions for which there was a significant difference between the mean values of the β functions for drawing versus locomotion (using the M-point). Every column represents a different figural form, where the cloverleaf is marked by $\mathcal{C}$. The marking $\mathcal{L}_1, \mathcal{L}_2, \mathcal{L}_3$ and $\mathcal{A}_1, \mathcal{A}_2, \mathcal{A}_3$ represent the limaçon and the lemniscate templates, respectively, according to the ascending ratio of the large to the small loops.

M points (using Wilcoxon rank sum test) reveals that locomotion data for all the figural forms are more Euclidian than the drawing data for these forms ($p < 0.005$), except for the cloverleaf using the R-point (see table S2).

The oblate limaçons are more affine in drawing than in locomotion, but the mean value of β_0 for the asymmetrical lemniscate is close to zero for both drawing and locomotion. For the cloverleaf we compared the locomotion data to the $\mathcal{C}_2$ shape, that is, the cloverleaf performed at medium speed.

The mixed ANOVA test on the drawing data shows significant differences among the means of the β functions of the different shapes ($p < 0.0001$ for β_0, $p = 0.0035$ for β_1 and $p = 0.0004$ for β_2), whereas among the subjects there are no statistically significant differences ($p > 0.7$ for all the βs).

Figure S10, first column, gives the means of the β functions for the drawing data of all the subjects for the different figural forms. Every subject is represented by a different color.

Compare the loops

For the oblate limaçons and asymmetric lemniscates we compared the means of the β functions for the different loops using Wilcoxon rank sum test. As in the analysis of the β functions for the different shapes, we considered only trials with $R^2 \geq 0.6$. See Figure S11.

We found no statistically significant difference among the values of the β functions on the small vs. large loops of these curves for all the different types of drawn oblate limaçons. On the other hand, con-

sidering the M-point in locomotion, we found that in the case of $\mathcal{L}_2$ the large loops were more Euclidian, less equi-affine and less affine than the small loops. The mean value of β_2, $\overline{\beta_2}$, was 0.43 on the large loops and 0.22 on the small loops. For the M-point the mean value of β_1, $\overline{\beta_1}$, varied between 0.54 on the large loops to 0.7 on the small loops and the mean value of β_0, $\overline{\beta_0}$, increased from 0.02 for the large loops to 0.07 for the small loops. The same kind of change was found for the R-point, but with less statistical significance. In the case of $\mathcal{L}_1$ there is only a difference for $\overline{\beta_2}$. As with $\mathcal{L}_2$ the outer loops were more Euclidian than the inner loops (this difference is not significant with the R-point).

Remark: For $\mathcal{L}_3$ of locomotion, some subjects generated very small inner loops which were impossible to separate appropriately from the large loops. Hence, we did not calculate the means of the β functions on the different loops of $\mathcal{L}_3$.

With asymmetric lemniscates, for $\mathcal{A}_1$, there is no difference between the values of the means of the β functions for drawing and locomotion, as expected. In the case of $\mathcal{A}_1$, the size of the upper loops equaled that of the lower loops.
The values of $\overline{\beta_0}$ and $\overline{\beta_2}$ are directly related to the size of the loops in locomotion for $\mathcal{A}_2$ and $\mathcal{A}_3$. The influence of Euclidian geometry, $\overline{\beta_2}$, changed from $0.38\% - 0.39\%$ on the small loops to $0.54\% - 0.56\%$ on the large loops (M-point). The changes in the influence of affine geometry as expressed by the values of $\overline{\beta_0}$ were smaller but still statistically significant, changing from 0.07 on the small loops to 0.1 on the large loops for the $\mathcal{A}_2$ shape and from 0.06 on the small loops to 0.09 on the large loops for the $\mathcal{A}_3$ shape. On the other hand, the influence of equi-affine geometry deceased from $0.54 - 0.55$ on the small loops to $0.35 - 0.36$ on the large loops (M-points). The same tendency can be seen with the R-points.

In drawing, as in locomotion, $\overline{\beta_0}$ increases from the small loops to the large loops in $\mathcal{A}_2$ and $\mathcal{A}_3$. However, unlike locomotion, in drawing the values of β_2 expressing the Euclidian influence did not change for $\mathcal{A}_2$ ($\overline{\beta_2} = 0.13$) and decreased from 0.2 on the small loops to 0.16 on the large loops in $\mathcal{A}_3$. A small statistically significant difference can be seen between $\overline{\beta_1}$ of 0.79 on the small loops and 0.75 on the large loops for $\mathcal{A}_2$. There were no differences in the values of $\overline{\beta_1}$ for $\mathcal{A}_3$.

E Third test: complex forms: timing

E.1 Proportion and segmentation

Our goal now is to indirectly test geometric segmentation of trajectories.

Let us consider a regularly connected trajectory Γ, which is closed and is sufficiently well practiced to insure a typical behavior during its generation. We suppose that the velocity on Γ is never zero and that it is continuous. We also assume the following hypothesis which we call mixed monotony:
Γ is composed of three parts, Γ_0, Γ_1 and Γ_2. On Γ_0 time is proportional to the affine parameter σ, on Γ_1 it is proportional to an equi-affine parameter σ_1 and on Γ_2 it is proportional to an Euclidian arc-length s. This is mathematically expressed by: $\Delta t_0 = C_0 \Delta\sigma, \Delta t_1 = C_1 \Delta\sigma_1, \Delta t_2 = C_2 \Delta s$, where C_0, C_1, C_2 are globally constant on each piece Γ_0, Γ_1 and Γ_2, respectively.

Roughly put, Γ_0 is the affine part of Γ, Γ_1 is its equi-affine part and Γ_2 is its Euclidian part. However, we do not suppose that Γ_2 consists of segments of straight lines nor that Γ_1 consists of parabolic arcs. But in order to define σ on Γ_0 and σ_1 on Γ_1, we must assume that Γ_0 does not contain inflection or parabolic points and that Γ_1 does not contain inflection points.

Let $\Lambda', \Lambda"$ be two sub-parts of Γ such that there is a planar affine transformation χ with $\chi(\Lambda') = \Lambda"$. Let us denote by T'_0, T'_1, T'_2 and $T"_0, T"_1, T"_2$ the time spent on the respective intersections $\Lambda'_0, \Lambda'_1, \Lambda'_2$ and $\Lambda"_0, \Lambda"_1, \Lambda"_2$ of Λ' with the three pieces of the partition $\Gamma_0, \Gamma_1, \Gamma_2$ of Γ. Also let us denote by $L'_2, L"_2$ the lengths of Λ'_2 and $\Lambda"_2$ and by $A'_1, A"_1$ the total sum of areas enveloped by Λ'_1, Λ''_1 and their chords. Then, from the invariance properties of the canonical parameters shown in the mathematical appendix, our hypothesis of local monotony implies the following scaling behaviors:

$$\frac{T"_0}{T'_0} = 1, \frac{T"_1}{T'_1} = [\frac{A"_1}{A'_1}]^{1/3}, \frac{T"_2}{T'_2} = \frac{L"_2}{L'_2}. \tag{S39}$$

Remark: by using the infinitesimal form of these conditions we can reciprocally deduce the hypothesis of mixed monotony.

Now consider a global affine transformation α. Let us apply it to the entire curve Γ, and let us assume that the image $\Gamma' = \alpha(\Gamma)$ is accordingly segmented and also satisfies *local monotony.* This implies precisely that on the three trajectories $\Gamma'_0 = \alpha(\Gamma_0), \Gamma'_1 = \alpha(\Gamma_1), \Gamma'_2 = \alpha(\Gamma_2)$ respectively, the motion should be affine, equi-affine and Euclidian monotonic. Then, under this hypothesis, we have the following lemma concerning the ratios of the times spent along the different parts of Γ and Γ':

Lemma 1.

Γ_0 is non-empty. Suppose that α is a translation or a similarity transformation, i.e., it transforms all lengths by the same ratio. Then, by denoting by T_0, T_1, T_2 and by T'_0, T'_1, T'_2 the times spent on corresponding parts in Γ and Γ', we have: $T'_2/T_2 = T'_1/T_1 = T'_0/T_0$. In other words, the ratios of the total times dedicated to the three different geometries are invariant to scaling by size.

Proof: In what follows, the index α is generally used to mark items on the transformed curve. For simplicity, we denote the norms $\|\overrightarrow{V}\|$ of velocities $\overrightarrow{V}$ by the letter V.
If λ_α is the dilatation factor of the transformation α, for any point M on Γ we have $\|\alpha(\overrightarrow{V(M)})\| = \lambda_\alpha V(M)$.

We first prove that if Λ is a connected component of Γ_0, Γ_1 or Γ_2 and if P is one of the extremities of Λ, then the ratio T_α/T of the times spent respectively on $\alpha(\Lambda)$ and Λ is equal to the ratio $\lambda_\alpha V(P)/V(\alpha(P))$.

Let us denote by D_α this ratio $\lambda_\alpha V(P)/V(\alpha(P))$. Let Λ_2 be an Euclidian connected part inside Γ_2 with extremities P, Q. We know that V is constant along Λ_2 and V_α is constant on $\alpha(\Lambda_2)$. Then by continuity of the velocities, for any point M on Λ_2 we have $\lambda_\alpha V(M) = D_\alpha V_\alpha(M)$.
Let us denote the lengths of Λ_2 and $\alpha(\Lambda_2)$ by L and L_α respectively, and the total times spent along them by T and T_α. We obtain $L_\alpha = \lambda_\alpha L$ and $\lambda_\alpha T_\alpha/L_\alpha = D_\alpha T/L$, thus $T_\alpha = D_\alpha T$.

The argument on an equi-affine segment Λ_1 inside Γ_1 meeting Λ_0 or Λ_2 in one of its extremities is analogous, with the enveloped areas replacing the generated distances:
Let P be the extremity where we already know that $\lambda_\alpha V(P) = D_\alpha V_\alpha(\alpha(P))$. Denote by μ_α the absolute value of the determinant of α. Let σ_1 be the standard equi-affine parameter on Λ_1 at P. Then, on Λ_1

we have $\Delta t = C_1 \Delta\sigma_1$ for some positive constant C_1. We know that the standard equi-affine parameter on $\alpha(\Lambda_1)$ is given by

$$\sigma_{1,\alpha}(\alpha(M)) = \mu_\alpha^{-1/3}\sigma_1(M), \tag{S40}$$

at every point M of Λ_1.

We obtain

$$\frac{d\sigma_{1,\alpha}}{dt_\alpha} = \frac{\mu^{-1/3}}{D_\alpha}\frac{d\sigma_1}{dt}. \tag{S41}$$

In another respect we also have $\Delta t' = C_1' \Delta\sigma_{1,\alpha}$ for some positive constant C_1'. We deduce

$$C_1' = \mu^{1/3} D_\alpha C_1. \tag{S42}$$

Now we are able to compute ratios of times T and T' spent on Λ_1 and its image $\alpha(\Lambda_1)$ respectively:

$$\begin{aligned} T' &= C_1' \Delta\sigma_{1,\alpha} \\ &= C_1' \mu^{-1/3} \Delta\sigma_1 \\ &= C_1' \mu^{-1/3} C_1^{-1} T \\ &= D_\alpha T. \end{aligned} \tag{S43}$$

If Λ is a piece of Γ_0, where it is known that on an extremity P we have $\lambda_\alpha V(P) = D_\alpha V_\alpha(\alpha(P))$, then on this segment $\Delta t_\alpha = D_\alpha \Delta t$, and it follows that $T'/T = D_\alpha$ too.

Now the proof can be ended by recurrence. As before we demonstrate piece-by-piece that the ratio T'/T of times on corresponding segments Λ and Λ' in Γ and Γ' equals the same constant D_α.

Finally, by summation, we obtain the desired relationships, namely:

$$T_2'/T_2 = T_1'/T_1 = T_0'/T_0. \tag{S44}$$

(Here we used the fact that $a/b = c/d$ implies $(a+c)/(b+d) = a/b$ too.) Note the consequence: without assuming the same values for the constants C_0 on the different components of Γ_0 we have obtained that D_α is the same for all of them.

The spirit of the demonstration is simple: the initial condition at P allows removing the ambiguity regarding constants over Γ_2, Γ_1 and Γ_0.

The following result opens the way to a very testable law:

Theorem

Suppose we have a closed monotonic connected trajectory Γ as before, and that Γ is a reunion of two closed parts Γ', Γ"; then there exist 3 non-negative constants B_0, B_1, B_2, that depend only on Γ' and are invariant under similarity transformation of Γ', such that $B_0 + B_1 + B_2 = 1$, and

$$\frac{T"}{T'} = B_0 + B_1[\frac{A"_1}{A'_1}]^{1/3} + B_2\frac{L"_2}{L'_2}, \tag{S45}$$

where $T", T'$ mark the times spent on $\Gamma", \Gamma'$, also $A"_1, A'_1$ mark the sum of areas enveloped by the equi-affine parts $\Gamma"_1, \Gamma'_1$ and $L"_2, L'_2$ mark the lengths of the Euclidian parts $\Gamma"_2, \Gamma'_2$.

Proof: Let us represent the segmentation as follows: $\Gamma' = \Gamma'_0 + \Gamma'_1 + \Gamma'_2$ and $\Gamma" = \Gamma"_0 + \Gamma"_1 + \Gamma"_2$, we have an evident repartition of time:

$$\frac{T"}{T'} = \frac{T"_0 + T"_1 + T"_2}{T'_0 + T'_1 + T'_2}, \tag{S46}$$

and we deduce by monotony:

$$\frac{T"}{T'} = \frac{T'_0 + T'_1[\frac{A"_1}{A'_1}]^{1/3} + T'_2\frac{L"_2}{L'_2}}{T'_0 + T'_1 + T'_2}, \tag{S47}$$

which gives:

$$\frac{T"}{T'} = \frac{T'_0}{T'} + \frac{T'_1}{T'}[\frac{A"_1}{A'_1}]^{1/3} + \frac{T'_2}{T'}[\frac{L"_2}{L'_2}]. \tag{S48}$$

Now the theorem follows from the preceding lemma because it asserts that the fractions T'_0/T', T'_1/T', T'_2/T' are independent of the scale of the curve Γ'.

Corollary

When there exists a similarity transformation φ of ratio ρ such that $\varphi(\Gamma') = \Gamma"$ that respects the

segmentation, the following formula is true:

$$\frac{T"}{T'} = B_0 + B_1\rho^{2/3} + B_2\rho. \tag{S49}$$

Proof: We obtain this result because φ sends $\Gamma'_0, \Gamma'_1, \Gamma'_2$ onto $\Gamma"_0, \Gamma"_1, \Gamma"_2$ respectively, and the factor of dilatation for areas is the square of the factor for dilating the lengths.

Note that if we consider a sequence of curves $\Gamma_{(n)}, n = 1, 2, ..., N$, decomposed as above into two parts $\Gamma'_{(n)}, \Gamma"_{(n)}$, which comply with the assumptions of the corollary with ratios $\rho_{(n)}, n = 1, 2, ..., N$, and such that all the $\Gamma'_{(n)}, n = 1, 2, ..., N$ are similar to a fixed Γ', we get for $n = 1, 2, ..., N$:

$$\frac{T"_{(n)}}{T'_{(n)}} = B_0 + B_1[\rho_{(n)}]^{2/3} + B_2\rho_{(n)}, \tag{S50}$$

with constants B_0, B_1, B_2 which are independent of n satisfying $B_0 + B_1 + B_2 = 1$.

This is the expected simple law that segmentation gives for the proportion of movement durations.

E.2 Data recording and processing

The data for testing our predictions regarding the durations of the different segments are those used in the section examining the different models of velocity prediction (see section D.1).

To obtain the lengths of the different loops in the asymmetric lemniscate (Figure S2A) or the oblate limaçon (Figure S2B) for both the drawing and locomotion data, points P1 and P2 in Figures S2A and S2B, respectively, were manually detected. The sampling indices when entering and leaving the small loop of the oblate limaçon or the intersection point in the asymmetric lemniscate were derived. Based on these values the Euclidian lengths and movement durations of the two loops of these shapes were accordingly calculated. To achieve greater accuracy, for locomotion this analysis was applied to the raw position data before smoothing.

References

[1] Klein F (1926) Vorlesungen ber die Entwicklung der Mathematik im 19. Berlin: Jahrhundert.

[2] Lie S, Engel F (1930) Theorie der Transformationsgruppen, zweiter Abschnitt. Teubner BG.

[3] Olver PJ (1995) Equivalence, Invariants, and Symmetry. Cambridge: Cambridge University Press.

[4] Cartan E (1937) La Théorie des Groupes Finis et Continus et la Géométrie Différentielle Traitées par la Méthode du Repère Mobile. Gauthier-Villars, Paris: Cahiers Scientifiques, Vol. 18.

[5] Olver PJ (2005) A survey of moving frames. In: Li H, Olver PJ, Sommer G, editors, Computer Algebra and Geometric Algebra with Applications, Lecture Notes in Computer Science, New york: Springer-Verlag, volume 3519. pp. 105–138.

[6] Faugeras O (1994) Cartan's moving frame method and its application to the geometry and evolution of curves in the euclidean, affine and projective planes. In: Mundy JL, Zisserman A, Forsyth D, editors, Applications of Invariance in Computer Vision, New York: Springer-Verlag, volume 825. pp. 11-46.

[7] Blaschke WJE (1921) Vorlesungen ober Differentialgeometrie. Springer coll. Grundlehren der mathematischen Wissenschaften.

[8] Lacquaniti F, Terzuolo C, Viviani P (1983) The law relating the kinematic and figural aspects of drawing movements. Acta Psychologica 53: 115–130.

[9] Viviani P, Terzuolo C (1982) Trajectory determines movement dynamics. Neuroscience 7: 431–437.

[10] Viviani P, McMollum G (1983) The relation between linear extent and velocity in drawing movements. Neuroscience 10: 211–218.

[11] Viviani P (1986) Do units of motor action really exist. In: Heuer H, Fromm C, editors, Generation and modulation of action patterns, Berlin: Springer, volume 15. pp. 201–216.

[12] Viviani P, Cenzato M (1985) Segmentation and coupling in complex movements. J Environ Psychol 11: 828–845.

[13] Viviani P, Schneider R (1991) A developmental study of the relationship between geometry and kinematics in drawing movements. J Exp Psychol Hum Percept Perform 17: 198–218.

[14] Pollick FE, Sapiro G (1997) Constant affine velocity predicts the 1/3 power law of planar motion perception and generation. Vision Research 37: 347–353.

[15] Flash T, Handzel AA (1996) Affine differential geometry of human arm trajectories. Soc Neurosci Abstr 22: 1635.

[16] Flash T, Handzel AA (2007) Affine differential geometry analysis of human arm movements. Biol Cybern 96: 577-601.

[17] Greenhill AG (1959) The Applications of Elliptic Functions. New York: Dover.

[18] Viviani P, Flash T (1995) Minimum-jerk, two-thirds power law, and isochrony: Converging approaches to movementnext term planning. J Exp Psychol Hum Percept Perform 21: 32–53.
